# Targeting the GPX4–FUNDC1 Interaction with Magnesium Lithospermate B Attenuates Sepsis‐Associated Lung Injury

**DOI:** 10.1002/advs.202516488

**Published:** 2026-01-30

**Authors:** Zhixi Li, Chang Liu, Zhaoxue Ma, Dongyou Zheng, Renkai Wang, Yongjing Yu, Guangmin Chen, Chenglong Li, Yue Bu, Hang Cao, Bing Zhang

**Affiliations:** ^1^ Department of Anesthesiology Second Affiliated Hospital of Harbin Medical University Harbin P. R. China; ^2^ The Key Laboratory of Anesthesiology and Intensive Care Research of Heilongjiang Province Harbin P. R. China; ^3^ The Key Laboratory of Myocardial Ischemia Organization Chinese Ministry of Education Harbin P. R. China; ^4^ State Key Laboratory of Frigid Zone Cardiovascular Diseases Harbin P. R. China; ^5^ Department of Anesthesiology Harbin Medical University Cancer Hospital Harbin P. R. China; ^6^ Department of Anesthesiology First Affiliated Hospital of Harbin Medical University Harbin P. R. China; ^7^ Department of Anesthesiology Fourth Affiliated Hospital of Harbin Medical University Harbin P. R. China; ^8^ Department of Pain Medicine Second Affiliated Hospital of Harbin Medical University Harbin P. R. China

**Keywords:** endothelial cells, ferroptosis, lung injury, mitophagy, sepsis

## Abstract

Sepsis‐associated lung injury (SALI) remains a critical clinical challenge, partly driven by ferroptosis‐induced endothelial dysfunction. The pathological interaction between FUN14 domain‐containing protein 1 (FUNDC1) and glutathione peroxidase 4 (GPX4) promotes ferroptosis and disrupts mitophagic flux. Magnesium lithospermate B (MLB), an active compound derived from *Salvia miltiorrhiza*, possesses anti‐inflammatory and antioxidant properties and exhibits potential for vascular protection. Here, it is demonstrated that MLB mitigates sepsis‐associated pulmonary vascular injury by suppressing ferroptosis and restoring mitochondrial homeostasis. Mechanistically, MLB directly binds GPX4 at Gly79, thereby disrupting the GPX4‐FUNDC1 interaction, stabilizing GPX4 enzymatic activity, and preventing its FUNDC1‐mediated mitophagic degradation. To enhance pulmonary targeting, P‐selectin‐binding peptide‐engineered adipose‐derived stem cell extracellular vesicles were constructed to deliver MLB, substantially improving its therapeutic efficacy in SALI. Furthermore, a silver‐citrate nanostructure‐based surface‐enhanced Raman spectroscopy platform was developed, enabling precise identification of MLB's Raman fingerprint spectrum with nanogram‐level sensitivity and time‐resolved in vivo biodistribution profiling. Collectively, these findings reveal a novel therapeutic mechanism and efficacy of MLB in SALI, highlighting a promising translational strategy that integrates targeted drug delivery with molecular detection for potential clinical applications.

## Graphical abstract

The diagram depicts the endothelial‐protective mechanism of magnesium lithospermate B (MLB) in sepsis‐associated lung injury. MLB binds GPX4 at Gly79, disrupts its interaction with FUNDC1, prevents mitophagy‐mediated GPX4 degradation, restores mitophagic flux, reduces ROS, and limits ferroptosis. For translational application, MLB@PBP‐ADSC‐EVs provide endothelial‐targeted delivery, while Ag@CIT‐based SERS substrates enable sensitive in vivo detection.

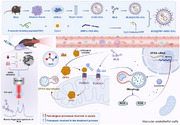

## 1 Introduction

Sepsis is a life‐threatening organ dysfunction resulting from dysregulated host immune responses to severe infection, representing a significant global health concern. Sepsis‐associated lung injury (SALI) is one of the most common and severe manifestations, characterized by non‐cardiogenic pulmonary edema and progressive hypoxemia, significantly contributing to high mortality rates in sepsis patients [1]. Despite substantial research efforts, effective therapeutic strategies for SALI remain scarce. Given the rapid progression of SALI, early and targeted intervention to mitigate lung injury and subsequent deterioration is critical to improving patient outcomes [2]. Pulmonary microvascular endothelial injury, an early hallmark of SALI, contributes to increased vascular permeability and compromised lung function, making the endothelium an attractive therapeutic target to halt disease progression [3, 4]. However, effective strategies to therapeutically target the endothelium remain elusive.

Ferroptosis, a form of regulated cell death driven by iron‐dependent lipid peroxidation, has emerged as a crucial pathological process influencing the prognosis of sepsis [5]. Recent studies, including our preliminary data, indicate heightened susceptibility of pulmonary vascular endothelial cells to ferroptosis during sepsis, underscoring that inhibiting ferroptosis could effectively protect endothelial function [6, 7]. Glutathione peroxidase 4 (GPX4), a key antioxidant enzyme that reduces lipid peroxides to their corresponding alcohols, plays a pivotal role in suppressing ferroptosis [8]. However, the marked decrease in GPX4 protein levels observed in SALI leads to heightened endothelial sensitivity to ferroptosis, vascular barrier disruption, and consequent pulmonary edema [7]. Currently, the mechanisms underlying GPX4 downregulation during SALI remain unclear, and no effective interventions have been developed to restore GPX4 levels and activity.

Mitophagy, a selective autophagic clearance of damaged or dysfunctional mitochondria, is crucial for maintaining cellular homeostasis and mitigating excessive inflammation and oxidative stress in various pathological conditions, including sepsis [9]. FUN14 domain‐containing protein 1 (FUNDC1), a mitochondrial outer membrane protein, has been shown to promote mitophagy through enhanced interaction with LC3B under inflammatory and oxidative stress conditions. Notably, enhancing FUNDC1‐mediated mitophagy has been associated with improved outcomes in sepsis models [10]. Recent evidence demonstrates a detrimental interaction between FUNDC1 and GPX4, wherein FUNDC1 recruits GPX4 into mitochondria, promoting its degradation via mitophagy. This interaction not only exacerbates ferroptosis but also diminishes the efficacy of FUNDC1‐mediated mitophagy itself [11]. Therefore, disrupting the GPX4‐FUNDC1 interaction offers a promising approach to mitigate endothelial injury and improve outcomes in SALI.

Magnesium lithospermate B (MLB), the most abundant bioactive component in *Salvia miltiorrhiza* aqueous extracts, exhibits potent antioxidant, mitochondrial protective, and anti‐inflammatory properties [12]. Previous studies have highlighted MLB's protective efficacy in organ injuries induced by inflammation and ischemia‐reperfusion [12, 13, 14]. Additionally, MLB is recognized for its significant endothelial protective effects [15]. However, the therapeutic potential and underlying mechanisms of MLB in SALI remain undefined.

In this study, we initially screened a library of small‐molecule compounds targeting GPX4 and identified MLB as an effective inhibitor of GPX4 degradation, subsequently alleviating ferroptosis. Our findings demonstrate that MLB directly interacts with GPX4, enhancing its enzymatic activity and preventing its mitophagy‐dependent degradation mediated by FUNDC1. Moreover, disrupting the GPX4‐FUNDC1 interaction restored FUNDC1‐dependent mitophagy efficiency. Significantly, combination therapy with MLB and the clinical trial compound MitoQ amplified protective effects against SALI. To further enhance targeted pulmonary delivery, we engineered adipose‐derived stem cell extracellular vesicles modified with a P‐selectin‐binding peptide (PBP)‐engineered adipose‐derived stem cell extracellular vesicle (ADSC‐EV) delivery system loaded with MLB (MLB@PBP‐ADSC‐EVs), markedly improving pulmonary distribution and therapeutic outcomes. Additionally, employing surface‐enhanced Raman spectroscopy (SERS) with a synthesized Ag@CIT substrate, we successfully captured MLB Raman signals in vivo, validating effective pulmonary targeting by MLB@PBP‐ADSC‐EVs. Collectively, this study identifies MLB as a promising small‐molecule candidate that preserves endothelial integrity in SALI by targeting the GPX4‐FUNDC1 interaction, while integrating targeted delivery and advanced detection techniques to establish translationally relevant therapeutic avenues.

## 2 Results

### 2.1 Endothelial‐Specific Deletion of GPX4 Aggravates Vascular Damage and Lung Injury

**FIGURE 1:**
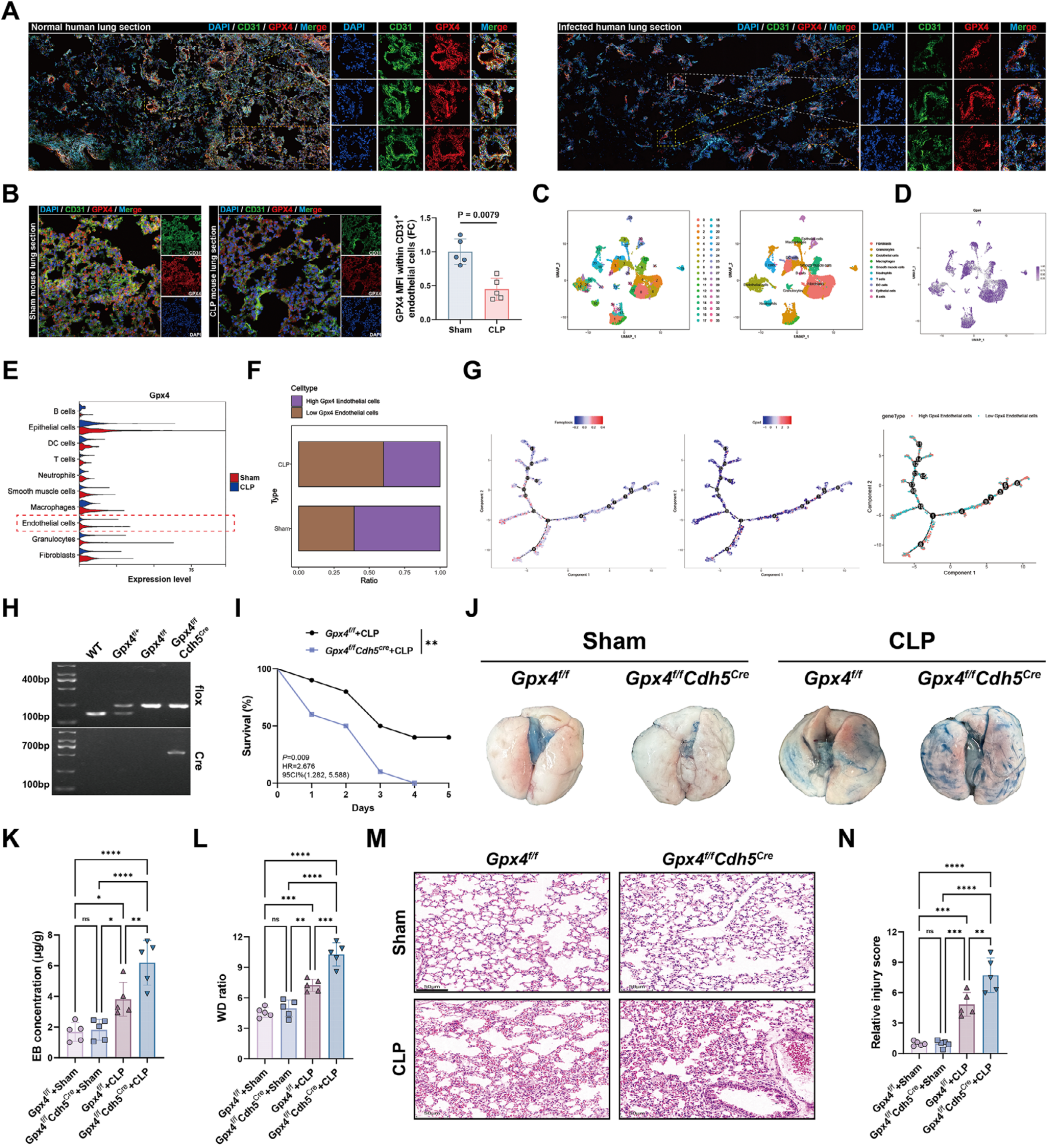
Endothelial GPX4 is downregulated in sepsis‐associated lung injury, and endothelial‐specific deletion of GPX4 exacerbates lung injury. (A) Representative immunofluorescence images of human lung tissue stained with DAPI (blue), CD31 (green), and GPX4 (red). Scale bar, 500 µm. (B) Representative immunofluorescence images and quantification of mouse lung tissue stained with DAPI, CD31, and GPX4 (*n* = 5). Scale bar, 20 µm. (C) UMAP plots showing cell clustering of mouse lung cells based on gene expression profiles (left) and annotation by cell types using canonical markers (right). (D) UMAP plot showing the distribution of *Gpx4* expression across all cell populations. (E) Violin plots of *Gpx4* expression levels across different lung cell types. (F) Bar graph showing the proportions of endothelial cells with high versus low GPX4 expression in sham and CLP‐treated mice. (G) Pseudotime trajectory analysis of endothelial cells, showing ferroptosis scores (low to high), the distribution of *Gpx4* expression, and classification into high‐ and low‐*Gpx4* expression groups. (H) Representative genotyping results showing detection of *Gpx4* floxed alleles and *Cdh5‐Cre* in WT, *Gpx4^f/f^*, and *Gpx4^f/f^Cdh5^Cre^*. (I) Survival curves of *Gpx4^f/f^* and *Gpx4^f/f^Cdh5^Cre^* mice subjected to CLP surgery (*n* = 20). (J,K) Relative Evans blue content in lung tissues (*n* = 5). (L) Quantification of the lung wet to dry weight ratio in mice (*n* = 5). (M,N) Representative H&E staining images of mouse lungs and corresponding lung injury scores (*n* = 5). Scale bar, 50 µm. Data are presented as mean ± SD. Statistical significance was determined using one‐way ANOVA with Tukey's post hoc test (for multiple comparisons) or log‐rank test (for survival analysis). *p < 0.05, **p < 0.01, ***p < 0.001, ****p < 0.0001, ns, not significant.

To examine whether GPX4 expression is altered in pulmonary endothelium under infectious conditions, we performed immunofluorescence staining on human lung tissues. Compared with non‐infected samples, infected lungs showed a pronounced reduction in CD31^+^GPX4^+^ endothelial cells, indicating suppressed GPX4 expression in inflamed endothelium (Figure 1A). Consistently, mouse lung tissues subjected to CLP exhibited a similar decrease in CD31^+^GPX4^+^ cells (Figure 1B). To validate these findings further, we reanalyzed publicly available single‐cell RNA‐seq data (GSE207651) from mouse lungs following CLP surgery. Uniform manifold approximation and projection (UMAP) analysis confirmed the presence of major lung cell populations (Figure 1C) and revealed a significant decrease in GPX4 expression specifically within endothelial cells from lung tissue of CLP‐treated mice (Figure 1D,E). Quantification revealed a reduced proportion of *Gpx4^high^* endothelial cells in CLP mouse lungs relative to sham controls (Figure 1F). Pseudotime trajectory analysis revealed that endothelial cells with high GPX4 expression and low ferroptosis scores were enriched at the root of the trajectory, whereas cells with reduced GPX4 and elevated ferroptosis potential appeared along the later branches (Figure 1G), indicating a potential transition toward ferroptotic vulnerability during disease progression.

To functionally evaluate the role of endothelial GPX4 in SALI, we generated endothelial‐specific *Gpx4* knockout mice (*Gpx4^f/f^Cdh5^Cre^*) using the Cre‐LoxP system (Figure 1H). Upon CLP challenge, *Gpx4^f/f^Cdh5^Cre^* mice exhibited significantly reduced survival compared to *Gpx4* ^f/f^ mice (Figure 1I), indicating a protective role for endothelial GPX4 during sepsis. Pulmonary vascular permeability, assessed by Evans blue (EB) dye extravasation and lung wet‐to‐dry weight (W/D) ratio, was markedly increased in CLP mice, with significantly higher levels observed in *Gpx4^f/f^Cdh5^Cre^* mice (Figure 1J–L; Table S1). H&E staining showed that lungs from both genotypes displayed minimal alterations under sham conditions. In contrast, CLP induced alveolar wall thickening, inflammatory infiltration, and architectural disruption, which were more pronounced in *Gpx4^f/f^Cdh5^Cre^* mice. Corresponding lung injury scores confirmed a significant exacerbation of lung damage upon endothelial GPX4 deletion (Figure 1M,N). Collectively, these results indicate that GPX4 expression is downregulated in pulmonary endothelial cells during sepsis and that its endothelial‐specific deletion exacerbates vascular permeability, inflammation, and lung injury.

### 2.2 MLB Directly Binds to GPX4, Enhances Its Enzymatic Activity, and Alleviates Lipid Peroxidation in Endothelial Cells

**FIGURE 2:**
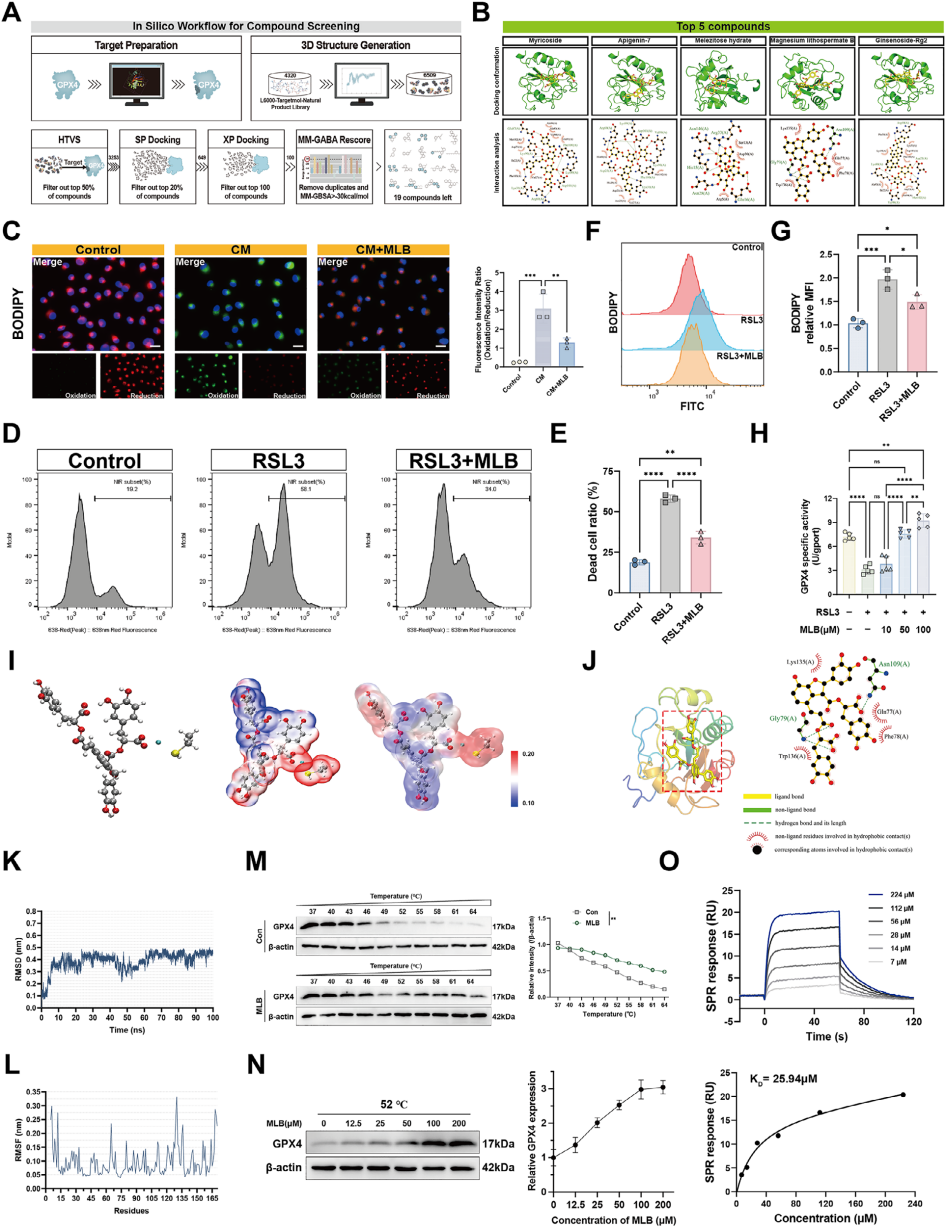
MLB directly binds to GPX4, enhances its enzymatic activity, and reduces lipid peroxidation in HPMECs. (A) Schematic workflow of the in silico compound screening pipeline, including structure preparation, virtual docking, and MM‐GBSA rescoring. (B) Representative docking poses and pharmacophore interaction maps of the top five screened compounds ranked by binding affinity. (C) Representative images and quantification of lipid peroxidation levels in HPMECs using C11‐BODIPY staining under different treatment conditions (*n* = 3). Scale bar, 50 µm. (D,E) Representative flow cytometry plots and quantification of Zombie NIR staining showing the percentage of dead cells in HPMECs treated with RSL3 in the presence or absence of MLB (*n* = 3). (F,G) Flow cytometry histograms and corresponding quantification of C11‐BODIPY fluorescence in HPMECs (*n *= 3). (H) Enzymatic activity of GPX4 protein after treatment with increasing concentrations of MLB, with or without the GPX4 inhibitor RSL3 (*n* = 5). (I) Structural characterization of the MLB‐GPX4 interaction. (J) Molecular docking model showing the binding interface between MLB and GPX4. (K,L) Molecular dynamics simulation results of the MLB‐GPX4 complex: root mean square deviation (RMSD, (K) and root mean square fluctuation (RMSF, (L) over 100 ns. (M) Temperature‐shift cellular thermal shift assay (CETSA) showing thermal stabilization of endogenous GPX4 in HPMECs pretreated with vehicle or MLB (*n* = 3). (N) Single‐temperature dose‐response CETSA performed at 52°C of GPX4 in response to MLB (12.5–200 µm) (*n* = 3). (O) Surface plasmon resonance (SPR) analysis of MLB and recombinant GPX4. Data are presented as mean ± SD. Statistical significance was determined using one‐way ANOVA with post hoc tests, except for CETSA data (M), which were analyzed by two‐way ANOVA with Sidak's multiple comparisons test. *p < 0.05, **p < 0.01, ***p < 0.001, ****p < 0.0001, ns, not significant.

Given the pivotal role of GPX4 in suppressing ferroptosis and maintaining redox homeostasis, we conducted an in silico screening of a natural product library to identify potential GPX4‐targeting compounds. The multi‐step virtual screening pipeline included HTVS, SP/XP docking, and MM‐GBSA rescoring, resulting in 19 top candidates (Figure 2A; Table S2). Based on binding affinity and known bioactivity profiles, Magnesium lithospermate B (MLB) was selected for further evaluation (Figure 2B). To evaluate the functional impact of MLB during sepsis, we exposed HPMECs to conditioned medium (CM) derived from LPS‐stimulated macrophages. C11‐BODIPY staining revealed elevated lipid peroxidation in CM‐treated cells, which was significantly attenuated by MLB treatment (Figure 2C), indicating its antioxidative potential. To determine whether MLB directly counteracts ferroptosis induced by GPX4 inhibition, we treated HPMECs with RSL3. MLB significantly reduced RSL3‐induced cell death and lipid peroxidation (Figure 2D–G), reinforcing its role as a ferroptosis suppressor. Functionally, MLB dose‐dependently enhanced GPX4 enzymatic activity and partially rescued its inhibition by RSL3 (Figure 2H). To explore potential interaction mechanisms at the atomic level, we conducted electrostatic potential (ESP) analysis using density functional theory (DFT). Ball‐and‐stick and ESP mapping indicated that MLB is spatially positioned near the selenium‐containing side chain of Sec46 (Figure 2I). Notably, regions of negative potential on MLB aligned with the electrophilic selenium center, suggesting possible weak orbital interactions that may contribute to allosteric regulation of GPX4 activity.

We next investigated whether MLB directly modulates GPX4. Molecular docking revealed a stable MLB‐GPX4 complex with a predicted binding free energy of −8.89 kcal/mol (Figure 2J). Consistently, molecular dynamics simulations confirmed stable binding, showing convergence at ∼10 ns and sustained RMSD stability throughout 100 ns (Figure 2K; Movie S1). Root mean square fluctuation (RMSF) analysis further indicated low atomic fluctuation at key binding regions, particularly near residues 75 and 135 of GPX4 (Figure 2L). To validate target engagement in cells, we performed cellular thermal shift assays (CETSA). Temperature‐shift assays showed that MLB pretreatment increased the thermal stability of endogenous GPX4 (Figure 2M). A single‐temperature CETSA at 52°C further revealed a dose‐dependent increase in stabilized GPX4 across 12.5–200 µm MLB (Figure 2N). Surface plasmon resonance (SPR) further validated the direct interaction between MLB and recombinant GPX4, with a dissociation constant of 25.94 µm (Figure 2O). Collectively, these results demonstrate that MLB directly binds to GPX4, enhances its enzymatic function, and mitigates lipid peroxidation in endothelial cells, supporting its potential as a GPX4‐targeting modulator of ferroptosis.

### 2.3 MLB Alleviates Ferroptosis and Barrier Dysfunction in Endothelial Cells During Sepsis

**FIGURE 3:**
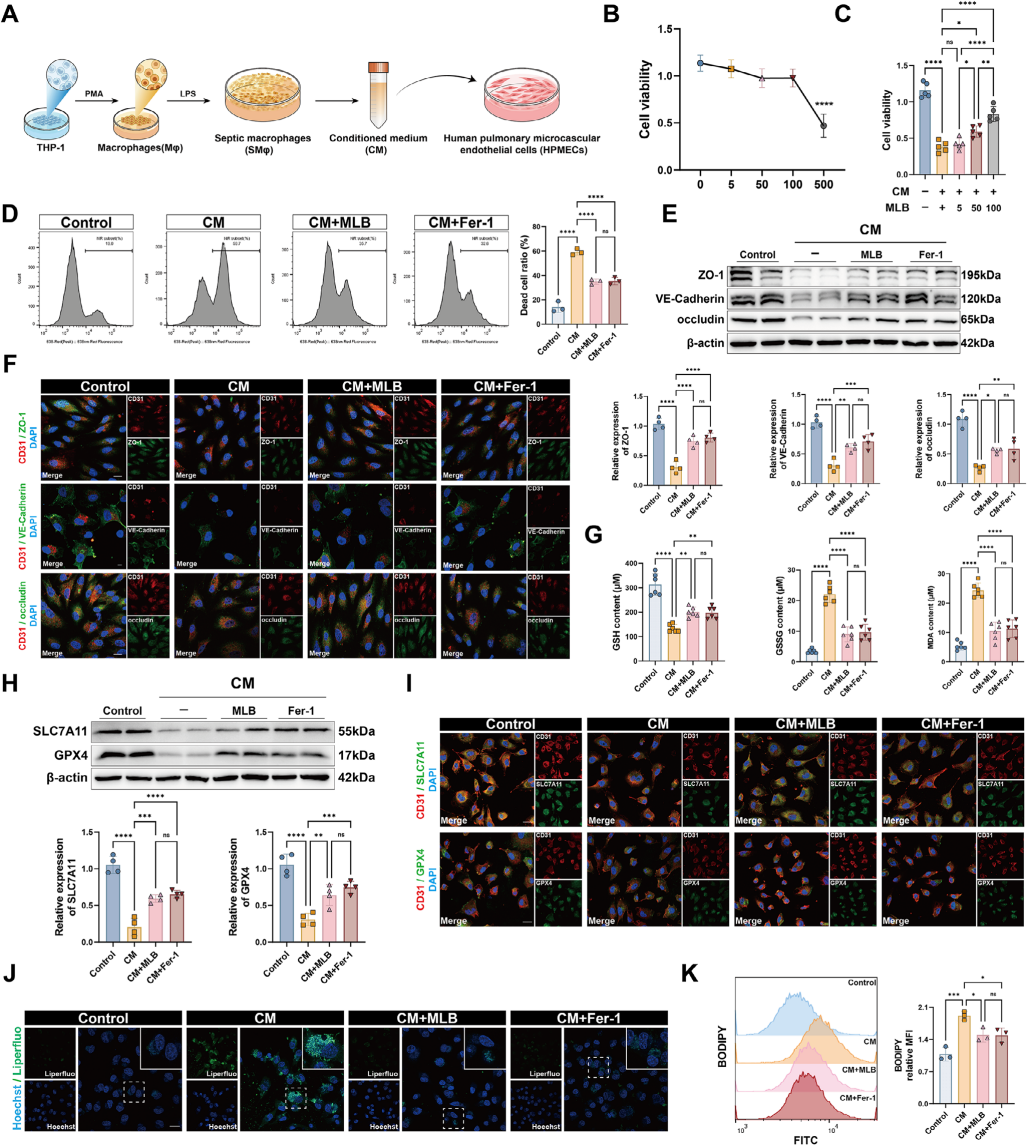
MLB attenuates sepsis‐induced ferroptosis and barrier dysfunction in HPMECs. (A) Schematic illustration of the experimental design. (B) Dose‐response curve of MLB on HPMEC viability assessed by CCK‐8 assay (*n* = 5). (C) Quantification of CCK‐8 assay in HPMECs co‐cultured with CM and treated with varying concentrations of MLB (*n* = 5). (D) Flow cytometry analysis and quantification of Zombie NIR‐positive dead cells in HPMECs treated with CM, with or without MLB or ferrostatin‐1 (Fer‐1) (*n *= 3). (E) Representative immunoblots and quantification of tight junction‐related proteins (ZO‐1, VE‐Cadherin, and occludin) in HPMECs (*n* = 4). (F) Immunofluorescence staining of ZO‐1, VE‐Cadherin, and occludin (green) with CD31 (red) and DAPI (blue) in HPMECs. Scale bar, 20 µm. (G) Quantification of oxidative stress parameters: intracellular GSSG, GSH, and MDA levels in HPMECs (*n* = 6). (H) Immunoblotting and quantification of ferroptosis‐related proteins SLC7A11 and GPX4 in HPMECs (*n* = 4). (I) Representative immunofluorescence staining of SLC7A11 (green) or GPX4 (green) with CD31 (red) and DAPI (blue) in HPMECs. Scale bar, 20 µm. (J) Representative fluorescence images of lipid peroxide levels in HPMECs detected by Liperfluo staining. Scale bar, 20 µm. (K) C11‐BODIPY flow cytometry assay evaluating lipid ROS accumulation in HPMECs (*n* = 3). Data are presented as mean ± SD. Statistical significance was determined using one‐way ANOVA with post hoc tests. *p < 0.05, **p < 0.01, ***p < 0.001, ****p < 0.0001, ns, not significant.

To further investigate the protective effects of MLB, we established an in vitro sepsis model by stimulating THP‐1‐derived macrophages with LPS and collecting their CM to treat HPMECs (Figure 3A). Cytotoxicity analysis revealed that high concentrations of MLB (500 µm) reduced cell viability, whereas 100 µm was well‐tolerated (Figure 3B). Notably, MLB significantly restored cell viability suppressed by CM exposure (Figure 3C). Flow cytometry analysis showed increased cell death in CM‐treated HPMECs, as indicated by elevated Zombie NIR positivity. This effect was effectively reversed by MLB or the ferroptosis inhibitor ferrostatin‐1 (Fer‐1), with no significant difference between the two treatments (Figure 3D). Western blot and immunofluorescence analyses demonstrated that CM markedly downregulated tight junction proteins ZO‐1, VE‐Cadherin, and occludin, indicating endothelial barrier disruption. MLB and Fer‐1 partially restored the expression of these markers (Figure 3E,F; Figure S1A–C), suggesting that MLB mitigates CM‐induced endothelial barrier impairment. Assessment of redox status revealed that CM elevated GSSG and MDA levels while decreasing GSH, consistent with increased oxidative stress. These alterations were ameliorated by MLB or Fer‐1 treatment (Figure 3G). Additionally, CM significantly suppressed the expression of key ferroptosis regulators SLC7A11 and GPX4, both of which were partially rescued by MLB or Fer‐1, as shown by immunoblotting and immunofluorescence (Figure 3H,I; Figure S1D,E). To further validate lipid peroxidation, we performed Liperfluo staining and C11‐BODIPY staining. Both assays confirmed a substantial increase in lipid ROS in CM‐treated cells, which was reversed by MLB or Fer‐1 (Figure 3J,K; Figure S1F). Overall, the evidence indicates that MLB effectively attenuates CM‐induced ferroptosis and endothelial dysfunction through modulation of GPX4‐dependent antioxidant defense.

### 2.4 MLB Protects Pulmonary Vascular Endothelial Cells From Sepsis‐Induced Ferroptosis and Barrier Disruption via GPX4‐Dependent Mechanisms

To ensure the biosafety of MLB under physiological conditions, we first assessed its potential to induce inflammation in healthy mice. Intravenous administration of MLB (30 mg/kg) did not alter the levels of IL‐1β, TNF‐α, or IL‐6 in lung tissues compared to vehicle‐treated controls (Figure S2A–C). These results indicate that MLB does not trigger basal inflammatory responses in vivo. To investigate the in vivo protective effects of MLB, we administered low (15 mg/kg) and high (30 mg/kg) doses of MLB via tail vein injection 1 day before and 2 h after CLP surgery in mice (Figure S3A). Kaplan–Meier survival analysis revealed that the high‐dose MLB group (MLB‐H) exhibited a significantly improved survival rate compared with the CLP group, whereas the low‐dose group (MLB‐L) showed no statistical difference (Figure S3B; Table S3). ELISA of lung tissue homogenates demonstrated that proinflammatory cytokine levels (TNF‐α, IL‐6, and IL‐1β) were markedly elevated in CLP mice but were significantly reduced by MLB treatment, with MLB‐H showing a more pronounced effect (Figure S3C). In addition, immunofluorescence staining for F4/80 and Ly6G revealed that MLB treatment partially reduced CLP‐induced pulmonary infiltration of macrophages and neutrophils (Figure S3D,E). H&E staining revealed severe lung tissue injury in the CLP group, which was notably alleviated in both MLB‐L and MLB‐H groups, with the latter showing a more substantial reduction in lung injury scores (Figure S3F). Similarly, lung W/D ratios were significantly increased in CLP mice and effectively reduced following MLB treatment, particularly in the MLB‐H group (Figure S3G). In bronchoalveolar lavage fluid (BALF), total protein content and cell count were both significantly elevated after CLP, and were markedly suppressed by MLB‐H administration (Figure S3H,I). Evans blue dye extravasation assays indicated a significant increase in pulmonary vascular leakage in CLP mice, which was reversed by MLB‐H treatment (Figure S3J and Table S4). Furthermore, immunofluorescence staining showed MLB restored the expression of tight and adherens junction proteins ZO‐1, VE‐Cadherin, and occludin, which were disrupted in the CLP group (Figure S3K,L). Western blotting confirmed that MLB‐H significantly rescued the CLP‐induced downregulation of these barrier‐related proteins (Figure S3M,N).

**FIGURE 4:**
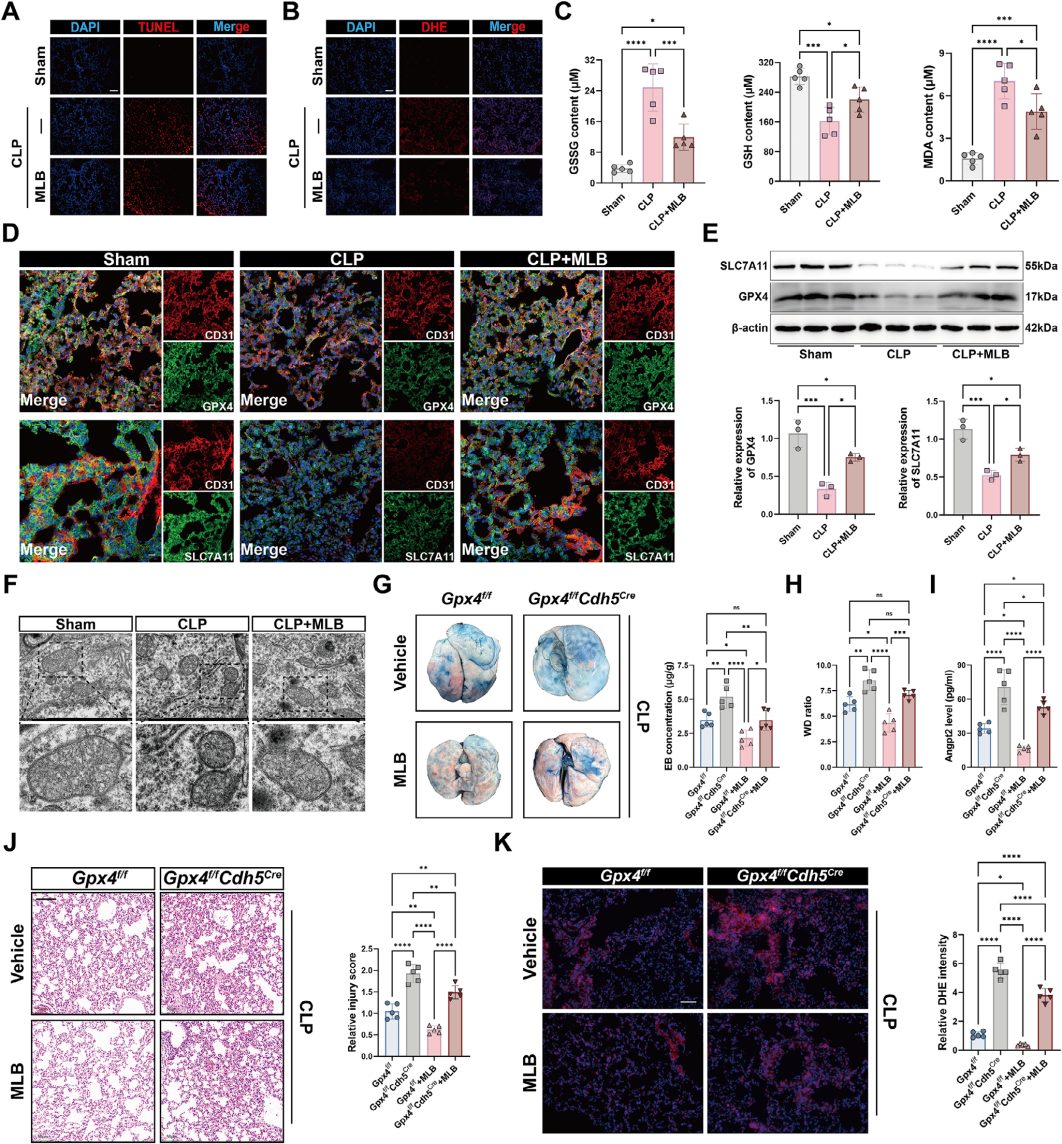
MLB alleviates pulmonary ferroptosis in CLP‐induced septic mice by upregulating GPX4. A,B) Representative images of TUNEL (A) and DHE (B) staining in lung tissues from sham, CLP, and CLP+MLB mice. Scale bar, 100 µm. (C) Quantification of oxidized glutathione (GSSG), reduced glutathione (GSH), and malondialdehyde (MDA) levels in lung tissues (*n* = 5). (D) Representative immunofluorescence images showing colocalization of CD31 (red) with GPX4 or SLC7A11 (green) in mouse lungs. Scale bar, 20 µm. (E) Immunoblot analysis and quantification of GPX4 and SLC7A11 expression in mouse lungs (*n* = 3). (F) Transmission electron microscopy (TEM) images of lung tissues showing mitochondrial morphology. Scale bar, 500 nm. (G) Representative images and quantification of Evans blue extravasation in lung tissues (*n* = 5). (H) Lung wet to dry weight ratio (*n* = 5). (I) Quantification of Angpt2 levels in mouse serum (*n *= 5). (J) H&E staining of lung sections and corresponding lung injury scores (*n* = 5). Scale bar, 100 µm. (K) Representative DHE staining images from *Gpx4^f/f^* and *Gpx4^f/f^Cdh5^Cre^* mice with or without MLB treatment (*n* = 5). Scale bar, 100 µm. Data are presented as mean ± SD. Statistical significance was determined by one‐way ANOVA followed by Tukey's post hoc test. *p < 0.05, **p < 0.01, ***p < 0.001, ****p < 0.0001, ns, not significant.

We next examined the effect of MLB on ferroptosis in the lungs of CLP‐treated mice. Based on the above findings, we selected the high dose of MLB (30 mg/kg) for subsequent experiments. TUNEL and DHE staining demonstrated a significant increase in dead and ROS‐positive cells in CLP mouse lungs, which was markedly reduced by MLB treatment (Figure 4A,B; Figure S4A,B). MLB also reversed CLP‐induced redox imbalance, as evidenced by reduced GSSG and MDA levels and increased GSH levels (Figure 4C). Immunofluorescence and immunoblotting revealed that GPX4 and SLC7A11 expression was downregulated in CLP mouse lungs and partially restored by MLB (Figure 4D,E; Figure S4C). Transmission electron microscopy showed mitochondrial shrinkage and increased membrane density in CLP mouse lungs, which were alleviated by MLB (Figure 4F), supporting the inhibition of ferroptosis at the ultrastructural level. In LPS‐challenged mice, MLB similarly increased pulmonary GPX4 and SLC7A11 expression, as shown by immunofluorescence and western blotting (Figure S4D–I).

To evaluate whether the protective effect of MLB depends on endothelial GPX4, we compared the responses of *Gpx4^f/f^* and *Gpx4^f/f^Cdh5^Cre^* mice treated with MLB following CLP induction. Compared with *Gpx4* ^f/f^ mice, *Gpx4^f/f^Cdh5^Cre^* mice exhibited significantly increased Evans blue extravasation (Figure 4G; Table S5), elevated lung wet to dry ratios (Figure 4H; Table S5), and higher serum levels of Angpt2 (Figure 4I). Histological assay further revealed aggravated lung injury and enhanced ROS accumulation in the *Gpx4^f/f^Cdh5^Cre^* mice despite MLB administration (Figure 4J,K). Immunoblotting analysis revealed that MLB‐treated *Gpx4^f/f^Cdh5^Cre^* mice exhibited significantly lower levels of endothelial junction proteins (ZO‐1, VE‐Cadherin, and occludin) and ferroptosis‐related markers (SLC7A11 and GPX4) compared to MLB‐treated *Gpx4^f/f^* mice (Figure S4J,K). In the LPS‐induced SALI model, endothelial‐specific GPX4 deletion blunted the lung‐protective effects of MLB (Figure S4L–O and Table S6). These findings indicate that MLB exerts its protective effects on the pulmonary vascular barrier primarily through a GPX4‐dependent mechanism.

### 2.5 MLB Stabilizes GPX4 and Preserves Mitochondrial Homeostasis in CM‐Treated HPMECs

**FIGURE 5:**
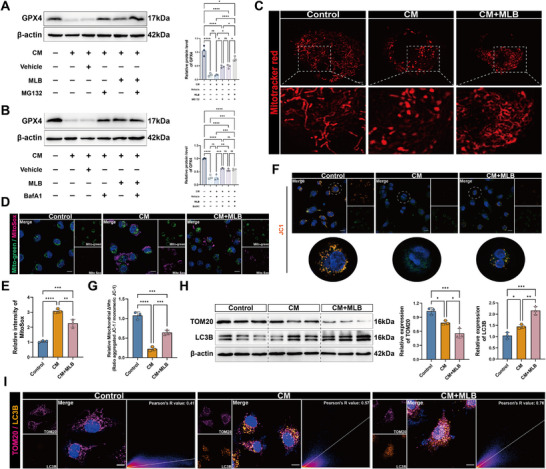
MLB restores mitochondrial function and enhances mitophagy in CM‐treated HPMECs. (A,B) Immunoblotting of GPX4 in HPMECs exposed to CM and treated with vehicle or MLB, with/without MG132 (A) or BafA1 (B). Quantification of GPX4 levels is shown (*n* = 3). (C) Representative confocal images of MitoTracker Red staining. Scale bar, 5 µm. (D,E) MitoSOX Red staining and quantification of mitochondrial ROS levels in HPMECs (*n* = 3). Scale bar, 10 µm. (F,G) Assessment of mitochondrial membrane potential (Δ*Ψm*) using JC‐1 staining (*n* = 3). Scale bar, 10 µm. (H) Western blot analysis and quantification of TOM20 and LC3B in HPMECs treated with CM or MLB (*n* = 3). (I) Immunofluorescence images showing colocalization of TOM20 and LC3B in HPMECs (*n* = 3). Scale bar, 10 µm. Data are presented as mean ± SD. Statistical significance was determined by one‐way ANOVA followed by Tukey's post hoc test. *p < 0.05, **p < 0.01, ***p < 0.001, ****p < 0.0001, ns, not significant.

To elucidate the mechanism by which MLB upregulates GPX4 expression, we investigated the post‐translational regulation of GPX4, given its confirmed binding to MLB. Since GPX4 degradation has been reported to occur through both proteasomal and lysosomal pathways [16, 17], we explored whether MLB affects either of these degradation mechanisms. We first treated HPMECs with the lysosomal inhibitor bafilomycin A1 (BafA1), which significantly increased LC3B and p62 levels, confirming effective inhibition of autophagic flux (Figure S5A). To further assess the contributions of the proteasomal and lysosomal pathways to GPX4 turnover, we performed cycloheximide (CHX) chase assays under CM treatment, with or without the proteasomal inhibitor MG132 or the lysosomal inhibitor BafA1. Both inhibitors markedly delayed GPX4 degradation over time, indicating that both pathways are involved in GPX4 turnover in HPMECs (Figure S5B,C). We next examined whether MLB interferes with these degradation routes. Under CM conditions, treatment with MG132, BafA1, or chloroquine (CQ) alone partially restored GPX4 protein levels. Co‐treatment with MLB and MG132 further elevated GPX4 levels compared to either treatment alone. In contrast, combining MLB with BafA1 or CQ did not result in additional upregulation beyond that achieved by MLB or lysosomal inhibition individually (Figure 5A,B; Figure S5D). Moreover, MLB treatment did not affect the ubiquitination level of GPX4 (Figure S5E). Collectively, these results suggest that MLB modulates GPX4 stability at least in part via the autophagy‐lysosome pathway under CM stress, rather than directly blocking proteasomal degradation.

Additionally, we reanalyzed single‐cell RNA‐sequencing (scRNA‐seq) data from CLP mouse lungs, focusing on pulmonary endothelial cells. KEGG pathway enrichment of differentially expressed genes between *Gpx4^high^* and *Gpx4^low^* cells revealed enrichment of mitophagy‐related pathways (Figure S5F), implying a potential link between GPX4 and mitochondrial homeostasis. Previous studies have shown that FUNDC1 recruits GPX4 to mitochondria, where mitophagy facilitates its degradation and promotes ferroptosis [11]. Given these findings, we speculate that MLB‐induced GPX4 upregulation may be mechanistically related to its effect on mitophagy.

To test this hypothesis, we evaluated mitochondrial integrity and mitophagic activity in HPMECs. MitoTracker Red staining revealed that mitochondria exhibited a filamentous morphology in the control group, while CM treatment caused pronounced mitochondrial fragmentation and shrinkage. MLB administration partially restored normal mitochondrial morphology (Figure 5C). Consistently, MitoSOX and JC‐1 staining demonstrated that CM elevated mitochondrial ROS levels and reduced membrane potential (Δ*Ψm*), both of which were significantly reversed by MLB treatment (Figure 5D–G). Western blot analysis further showed that MLB downregulated TOM20, while upregulating the autophagy marker LC3B (Figure 5H). Immunofluorescence staining revealed enhanced colocalization between TOM20 and LC3B in the MLB‐treated group compared with CM alone (Figure 5I), consistent with enhanced mitophagic activity. To further assess the compartment‐specific regulation of GPX4, we performed cytosolic and mitochondrial fractionation followed by immunoblotting. MLB treatment markedly increased GPX4 abundance in the cytosolic fraction while concurrently decreasing its levels in the mitochondrial compartment, suggesting that MLB may restrict the mitochondrial localization of GPX4 under sepsis‐induced stress conditions (Figure S5G). Together, these results suggest that MLB promotes mitophagy and preserves mitochondrial homeostasis in pulmonary endothelial cells during sepsis.

### 2.6 MLB Disrupts GPX4‐FUNDC1 Interaction and Alleviates GPX4 Degradation via Mitophagy

**FIGURE 6:**
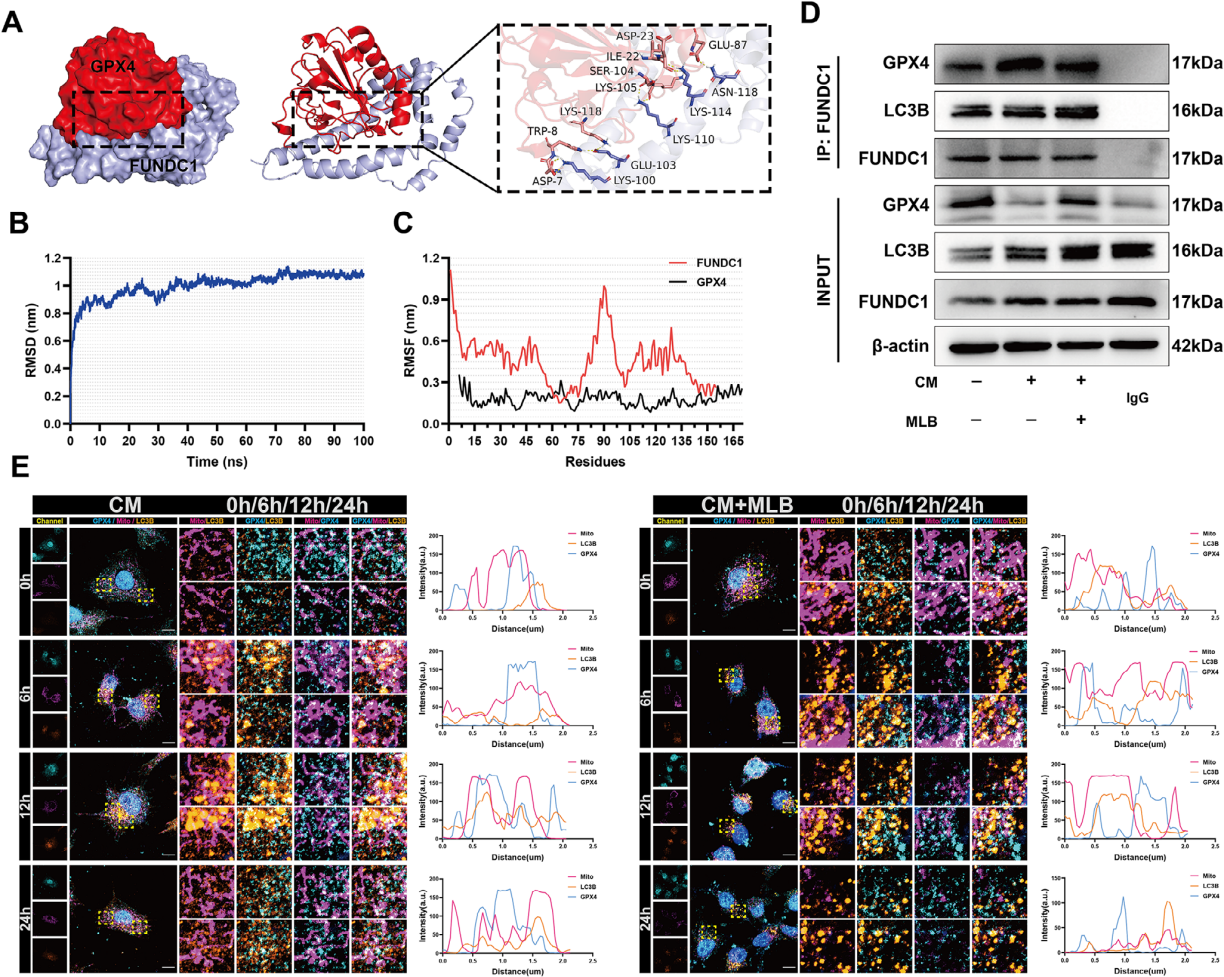
MLB disrupts GPX4‐FUNDC1 interaction and promotes FUNDC1‐LC3B binding. (A) Structural model of GPX4‐FUNDC1 complex showing the binding interface and key interacting residues. (B) Root mean square deviation (RMSD) and (C) root mean square fluctuation (RMSF) analyses of the GPX4‐FUNDC1 complex. (D) Co‐immunoprecipitation analyses showing the interactions of GPX4 and LC3B with FUNDC1 in HPMECs treated with conditioned medium (CM) with or without MLB. (E) Representative confocal images and quantitative colocalization analysis of DAPI, GPX4, LC3B, and MitoTracker in HPMECs at the indicated time points. Scale bar, 20 µm.

Previous studies have shown that FUNDC1 interacts with GPX4, which not only impairs the binding of FUNDC1 to LC3B and thereby reduces mitophagy efficiency, but also promotes the mitochondrial translocation of GPX4, facilitating its degradation via the mitophagy pathway [11]. Our preliminary data demonstrated that MLB simultaneously enhanced mitophagic activity and elevated GPX4 protein levels by inhibiting lysosome‐dependent degradation, prompting the hypothesis that MLB might exert its protective effect by disrupting the GPX4‐FUNDC1 interaction. To test this hypothesis, we performed protein–protein docking and molecular dynamics simulations to assess the stability of the GPX4‐FUNDC1 complex. The binding interface between GPX4 and FUNDC1 involved multiple electrostatic and hydrogen bonding interactions (Figure 6A). The result of RMSD showed that the GPX4‐FUNDC1 complex reached structural stability after approximately 10 ns (Figure 6B; Movie S2). RMSF analysis further revealed reduced flexibility in the region surrounding residue 75 of GPX4 (Figure 6C). Previous studies have shown that the interaction between GPX4 and FUNDC1 is primarily mediated by the 96–133 amino acid segment of FUNDC1 [11]. Consistent with this, our molecular dynamics simulations identified a binding hotspot within this region (Figure S6A,B). These results support the existence of a stable interaction between GPX4 and FUNDC1.

Co‐immunoprecipitation assays further confirmed that CM treatment enhanced GPX4‐FUNDC1 complex formation, whereas MLB significantly disrupted this interaction while promoting FUNDC1‐LC3B binding (Figure 6D). Proximity ligation assay (PLA) further confirmed that CM markedly enhanced GPX4‐FUNDC1 interaction in HPMECs, as indicated by increased PLA puncta, whereas MLB treatment abolished this effect (Figure S7A). CETSA showed that MLB did not increase FUNDC1 thermal stability (Figure S7B), indicating that MLB likely acts through GPX4 rather than FUNDC1. Confocal immunofluorescence imaging revealed that CM promoted GPX4‐LC3B colocalization over time, while MLB treatment reversed this trend and instead enhanced LC3B‐MitoTracker colocalization, consistent with increased mitophagy and reduced GPX4 mitochondrial degradation (Figure 6E). These results suggest that MLB disrupts the GPX4‐FUNDC1 complex, thereby promoting FUNDC1‐mediated mitophagy and alleviating mitophagic degradation of GPX4.

To investigate the role of FUNDC1 in regulating GPX4, we modulated FUNDC1 expression in HPMECs. Neither knockdown nor overexpression altered total GPX4 protein abundance under CM conditions (Figure S8A–G). Cytosolic and mitochondrial fractionation revealed a clear compartment‐specific shift: *FUNDC1* knockdown markedly reduced mitochondrial GPX4 while concomitantly increasing its cytosolic abundance, whereas *FUNDC1* overexpression produced the opposite pattern (Figure S8H,I). These findings indicate that FUNDC1 regulates GPX4 in a compartment‐restricted manner, primarily affecting its mitochondrial localization under CM stress. *GPX4* mRNA levels were positively correlated with FUNDC1 expression: *FUNDC1* silencing reduced, whereas *FUNDC1* overexpression increased *GPX4* transcription under CM stress, including in the presence of MLB (Figure S8J–L). Given that FUNDC1 regulates mitophagy and intracellular ROS levels—and ROS is known to suppress *GPX4* transcription [18] —FUNDC1 may indirectly modulate *GPX4* mRNA via oxidative stress. Meanwhile, FUNDC1‐dependent mitophagy may facilitate the turnover of mitochondrial GPX4. Together, this dual regulation at the transcriptional level and in a compartment‐linked manner may explain the minimal net change in total GPX4 protein abundance despite evident redistribution between mitochondrial and cytosolic pools.

To further validate this hypothesis, we assessed the effect of MLB on GPX4 expression in HPMECs. CM treatment markedly reduced *GPX4* mRNA levels compared with control, whereas MLB significantly reversed this suppression (Figure S9A). When MLB was co‐administered with either exogenous ROS (H_2_O_2_) or the NRF2 inhibitor ML385, GPX4 protein levels were only partially decreased but still remained higher than those in the CM group (Figure S9B). Interestingly, *GPX4* mRNA levels declined and were comparable to those in the CM group (Figure S9C). This discrepancy may be attributed to the concurrent role of MLB in preventing mitophagy‐mediated degradation of GPX4 protein.

We then investigated whether this regulatory effect of MLB also extends to apoptosis and pyroptosis. Immunoblotting showed that MLB markedly suppressed CM‐induced activation of cleaved caspase‐9 and caspase‐3, two key markers of apoptosis. This inhibitory effect was abolished by co‐treatment with H_2_O_2_, which restored their expression to levels comparable to those in the CM group (Figure S9D–F). Similarly, MLB significantly reduced the expression of gasdermin D (GSDMD) and cleaved caspase‐1, two hallmark effectors of pyroptosis, and this suppression was largely reversed by H_2_O_2_ co‐treatment (Figure S9G–I). Collectively, these results indicate that the anti‐apoptotic and anti‐pyroptotic effects of MLB are at least partly mediated by its capacity to mitigate oxidative stress.

### 2.7 MLB Targets Gly79 of GPX4 to Disrupt GPX4‐FUNDC1 Interaction, Thereby Preserving Endothelial Integrity and Mitochondrial Function in Sepsis

**FIGURE 7:**
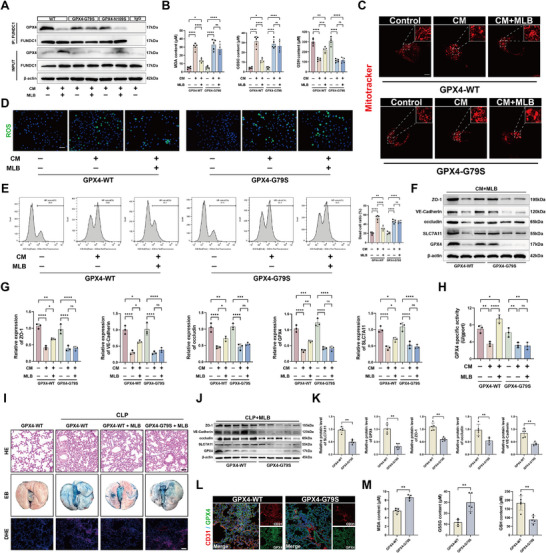
MLB interacts with GPX4 at Gly79 to disrupt GPX4‐FUNDC1 interaction and mitigate cellular injury and mitochondrial dysfunction. (A) Immunoblot analysis of GPX4 and FUNDC1 in HPMECs. Co‐immunoprecipitation was performed to assess GPX4‐FUNDC1 binding. (B) Quantification of MDA, GSSG, and GSH levels in HPMECs transfected with GPX4‐WT or GPX4‐G79S (*n* = 5). (C) Representative images of MitoTracker staining to assess mitochondrial morphology in HPMECs. Scale bar, 5 µm. (D) Fluorescence staining of intracellular ROS levels in HPMECs. Scale bar, 100 µm. (E) Flow cytometric analysis and quantification of cell death using Zombie NIR staining in HPMECs (*n* = 5). (F,G) Immunoblot analysis of ZO‐1, VE‐Cadherin, occludin, GPX4, and SLC7A11 expression in HPMECs transfected with wild‐type (WT) or G79S mutant GPX4 (*n *= 3). (H) GPX4 enzymatic activity (*n *= 3). (I) Representative images of lung histology, vascular permeability, and oxidative stress in septic mice. H&E staining, Evans blue (EB) extravasation, and DHE fluorescence are shown for GPX4‐WT+vehicle (baseline control), CLP+GPX4‐WT+vehicle (sepsis control), CLP+GPX4‐WT+MLB, and CLP+GPX4‐G79S+MLB groups, with identical AAV doses in WT/G79S groups to avoid confounding effects. Scale bar, 100 µm. (J,K) Immunoblot analysis and quantification of endothelial barrier and ferroptosis‐associated proteins (ZO‐1, VE‐Cadherin, occludin, SLC7A11, and GPX4) in lung tissues (*n* = 5). (L) Representative immunofluorescence images showing GPX4 and CD31 colocalization in lung sections. Scale bar, 20 µm. (M) Quantification of oxidative stress markers (MDA, GSSG, and GSH) in lung tissues (*n* = 5). Data are presented as mean ± SD. Statistical significance was determined by one‐way ANOVA followed by Tukey's post hoc test for (B,E,G,H) and unpaired two‐tailed Student's *t*‐test for (K,M). *p < 0.05, **p < 0.01, ***p < 0.001, ****p < 0.0001, ns, not significant.

Molecular docking analysis identified Gly79 and Asn109 of GPX4 as potential hydrogen‐bonding residues for MLB. To validate their roles, we generated single‐point mutants G79S (glycine‐to‐serine) and N109S (asparagine‐to‐serine). Plasmids encoding wild‐type GPX4 (GPX4‐WT), GPX4‐G79S, or GPX4‐N109S were transfected into HPMECs for functional characterization. Co‐immunoprecipitation demonstrated that MLB disrupted the GPX4‐FUNDC1 interaction in GPX4‐WT and GPX4‐N109S cells, whereas this effect was markedly attenuated in GPX4‐G79S cells (Figure 7A). Reciprocal IP confirmed these findings (Figure S10A). To directly assess the contribution of Gly79 to MLB binding, we purified recombinant GPX4‐WT and GPX4‐G79S proteins and performed differential scanning fluorimetry (DSF). MLB significantly increased the thermal stability of GPX4‐WT but exerted minimal stabilizing effects on GPX4‐G79S. In contrast, the structural analog Rosmarinic acid had no detectable effect on either protein (Figure S10B). Consistently, cellular CETSA showed that MLB increased the thermal stability of GPX4‐WT but not GPX4‐G79S (Figure S10C). Collectively, these assays indicate that Gly79 is critical for MLB‐GPX4 engagement and underlies MLB‐mediated disruption of the GPX4‐FUNDC1 complex.

Functionally, MLB failed to reverse CM‐induced elevations in MDA and GSSG levels or to restore GSH content in GPX4‐G79S cells (Figure 7B). ROS staining and MitoTracker assays demonstrated that MLB reduced oxidative stress and preserved mitochondrial morphology in GPX4‐WT cells, whereas these effects were abolished in the G79S mutant (Figure 7C,D; Figure S10D). Similarly, flow cytometry revealed that MLB significantly reduced CM‐induced cell death in GPX4‐WT cells but conferred minimal protection in G79S cells (Figure 7E). In GPX4‐G79S‐transfected HPMECs, MLB also failed to restore CM‐induced reductions in tight junction proteins and ferroptosis regulators (Figure 7F,G). Enzymatic activity assays further confirmed that the G79S mutation significantly impaired the restorative effect of MLB on GPX4 activity (Figure 7H).

CLP mice were injected via the tail vein with AAV vectors encoding GPX4‐WT or GPX4‐G79S, followed by MLB treatment. H&E staining revealed that MLB treatment significantly attenuated lung injury in GPX4‐WT mice but not in GPX4‐G79S mice, as indicated by higher injury scores in the latter (Figure 7I; Figure S10E). Evans blue extravasation and DHE staining showed increased vascular permeability and ROS accumulation in the lungs of GPX4‐G79S mice compared to GPX4‐WT (Figure 7I, Figure S10E, and Table S7). Western blot analysis confirmed that MLB‐induced upregulation of barrier proteins and ferroptosis suppressors was significantly diminished in GPX4‐G79S mice (Figure 7J,K). Immunofluorescence staining showed reduced colocalization of GPX4 and CD31 in the lungs of GPX4‐G79S mice (Figure 7L; Figure S10F). Quantification of oxidative stress markers further demonstrated that MLB significantly reduced MDA and GSSG levels and increased GSH content in GPX4‐WT lungs, but these effects were abrogated in the G79S mutant (Figure 7M). Collectively, these findings establish Gly79 of GPX4 as a critical determinant of MLB binding and therapeutic efficacy.

### 2.8 Combined MLB and MitoQ Treatment Provides Synergistic Protection Against SALI

**FIGURE 8:**
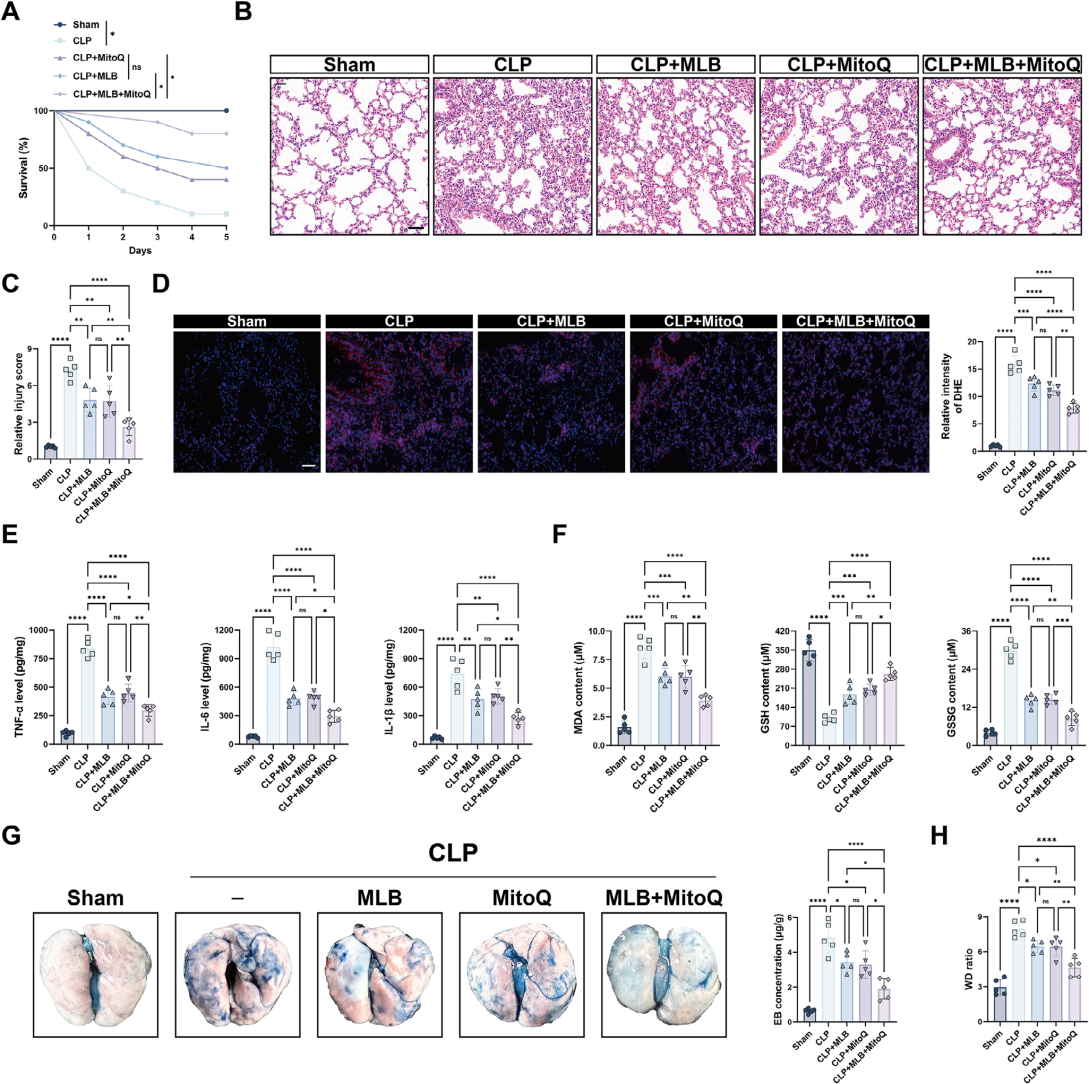
Combined treatment with MLB and MitoQ confers superior pulmonary protection in septic mice. (A) Kaplan–Meier survival curves of mice subjected to CLP and treated with vehicle, MLB, MitoQ, or their combination (*n* = 20). (B,C) Representative H&E staining of lung sections and quantification of lung injury scores (*n* = 5). Scale bar, 100 µm. (D) Representative images and quantitative analysis of DHE fluorescence in lung tissues (*n* = 5). Scale bar, 100 µm. (E) Quantification of pro‐inflammatory cytokines (IL‐6, TNF‐α, and IL‐1β) in lung tissues (*n* = 5). (F) Quantification of lipid peroxidation and redox markers (MDA, GSH, and GSSG) in mouse lungs (*n* = 5). (G) Representative images and quantification of Evans blue (EB) extravasation in lung tissues (*n* = 5). (H) W/D ratio in each group (*n* = 5). Data are presented as mean ± SD. Statistical significance was determined by one‐way ANOVA followed by Tukey's post hoc test. *p < 0.05, **p < 0.01, ***p < 0.001, ****p < 0.0001, ns, not significant.

Given that MLB targets both GPX4 and mitochondrial function, we investigated whether co‐administration with the mitochondria‐targeted antioxidant mitoquinone mesylate (MitoQ)—currently under clinical evaluation for endothelial protection and mitochondrial preservation [19, 20, 21]—could enhance therapeutic efficacy. To quantitatively evaluate the interaction between MLB and MitoQ, we performed a 6 × 6 dose‐response matrix in CM‐treated HPMECs using a CCK‐8 viability assay. Increasing concentrations of either agent alone modestly improved cell viability, whereas combined treatment produced higher viability than either monotherapy across most dose combinations, with the most pronounced effects observed at 25–50 µm MLB plus 0.25–0.5 µm MitoQ. Bliss independence analysis of the same data revealed an overall synergy score of 10.27, with a contiguous region of positive synergy scores within this concentration range (Figure S11A,B). Kaplan–Meier survival analysis showed that while MLB or MitoQ alone modestly improved survival after CLP surgery, whereas their combination significantly increased 5‐day survival compared with either monotherapy (Figure 8A; Table S8). Histological examination revealed that each agent partially alleviated CLP‐induced lung injury, and the combined treatment further reduced tissue damage, as indicated by lower lung injury scores (Figure 8B,C). DHE staining confirmed greater ROS clearance in the combination group relative to single‐agent treatment (Figure 8D). MLB‐MitoQ co‐treatment also markedly suppressed pulmonary levels of proinflammatory cytokines, including TNF‐α, IL‐6, and IL‐1β (Figure 8E). Similarly, oxidative stress markers reflected improved redox homeostasis: lipid peroxidation (MDA) was reduced, and GSH/GSSG balance was restored more efficiently by the combined therapy (Figure 8F). Notably, MLB+MitoQ provided superior vascular barrier protection, evidenced by reduced Evans blue extravasation and improved lung W/D ratios, indicating attenuated pulmonary edema (Figure 8G,H; Table S9). Together, these findings indicate that MLB and MitoQ act synergistically to mitigate SALI, while the underlying molecular basis remains to be clarified.

### 2.9 MLB@PBP‐ADSC‐EVs Enhance Pulmonary Targeting and Confer Superior Lung Protection in Sepsis

**FIGURE 9:**
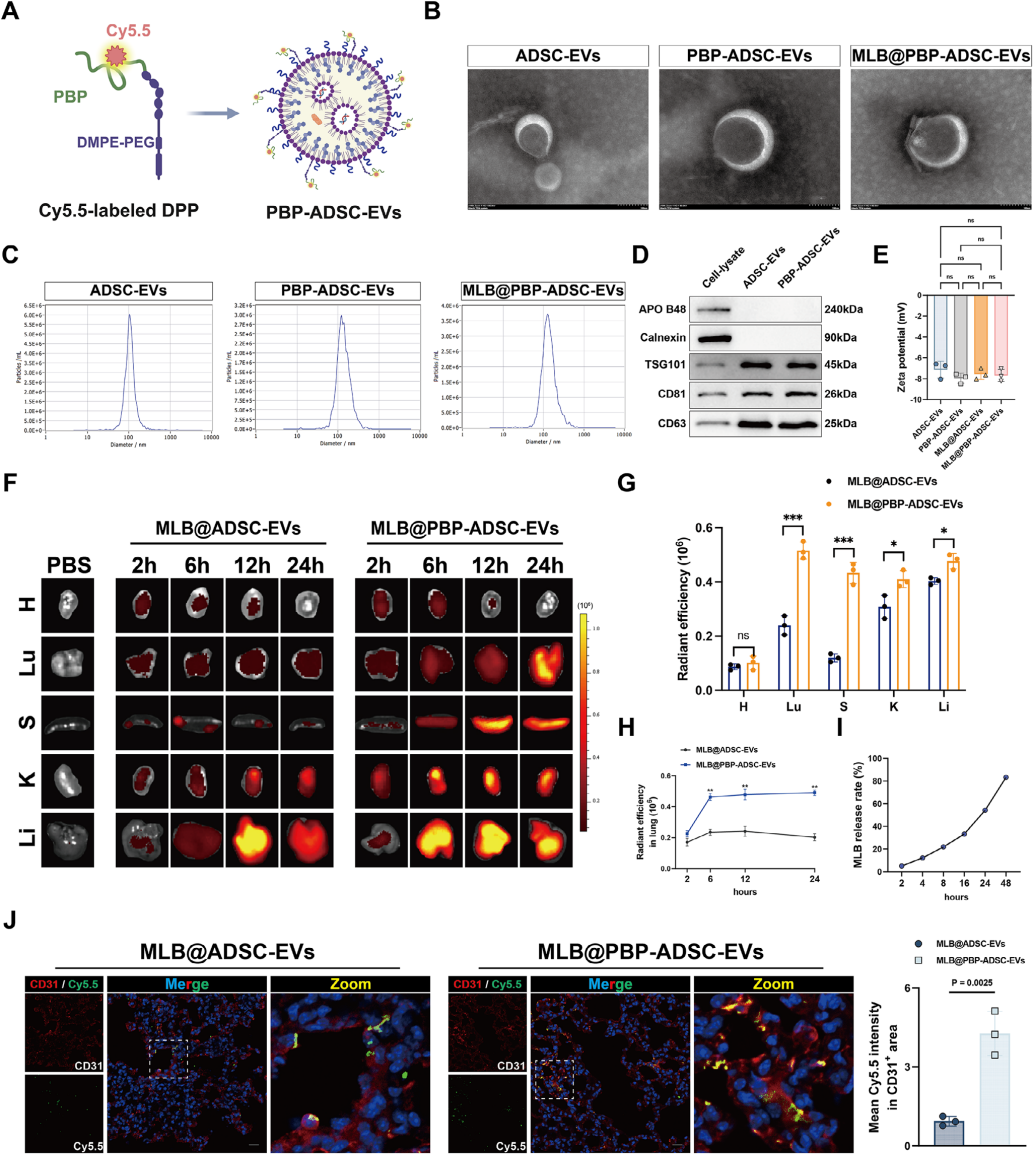
Targeted delivery of MLB@PBP‐ADSC‐EVs to the inflamed pulmonary endothelium in septic mice. (A) Schematic illustration of the construction of P‐selectin‐targeted EVs (PBP‐ADSC‐EVs). (B) Transmission electron microscopy (TEM) images showing the morphology of ADSC‐EVs, PBP‐ADSC‐EVs, and MLB@PBP‐ADSC‐EVs. Scale bar, 100 nm. (C) Nanoparticle tracking analysis (NTA) showing the size distribution profiles and average diameter of the indicated EVs. (D) Representative western blots showing the expression of EV markers (TSG101, CD81, CD63) and negative controls (Calnexin, APO B48). (E) Statistical graph of zeta potential measurements (*n* = 3). (F) Biodistribution of Cy5.5‐labeled MLB@ADSC‐EVs and MLB@PBP‐ADSC‐EVs in major organs (H: heart; Lu: lung; S: spleen; K: kidney; Li: liver) of CLP mice at 2, 6, 12, and 24 h after intravenous injection. (G) Quantification of fluorescence intensity in major organs at 24 h post‐injection (*n* = 3). (H) Quantitative analysis of Cy5.5 fluorescence intensity in the lung at the indicated time points post‐injection (*n* = 3). (I) In vitro release profile of MLB from MLB@PBP‐ADSC‐EVs over time. (J) Representative immunofluorescence images and quantitative analysis showing colocalization of CD31 (red) and Cy5.5‐labeled EVs (green) in mouse lung sections 12 h post‐injection (*n* = 3). Scale bar, 20 µm. Data are presented as mean ± SD. An unpaired two‐tailed Student's *t*‐test for (G,H,J) and one‐way ANOVA followed by Tukey's post hoc test for (E). *p < 0.05, **p < 0.01, ***p < 0.001, ****p < 0.0001, ns, not significant.

To improve the therapeutic efficacy of MLB in SALI, we developed a targeted delivery system based on engineered extracellular vesicles (EVs) derived from adipose‐derived stem cells (ADSCs). P‐selectin is rapidly upregulated on pulmonary vascular endothelial cells following injury, including in sepsis [22, 23, 24]. Immunofluorescence staining revealed a time‐dependent increase in endothelial P‐selectin expression of mouse lungs from 2 to 24 h post‐CLP (Figure S12A), supporting its potential as a targeting ligand. We next isolated and characterized ADSCs from mouse adipose tissue. The isolated cells exhibited fibroblast‐like morphology, adipogenic and osteogenic differentiation potential, and expressed canonical ADSCs surface markers (Figure S13A–D). To enable MLB delivery, ADSC‐EVs were incubated with MLB and subjected to ultrasound‐assisted loading, achieving an encapsulation efficiency of 42.16%. In parallel, P‐selectin‐binding peptide (PBP, CDAEWVDVS) was covalently conjugated to DMPE‐PEG‐MAL to generate DMPE‐PEG‐PBP (DPP), confirmed by the disappearance of the maleimide peak (6.8 ppm) and appearance of PBP‐related proton signals (7–7.6 ppm) in ^1^H NMR spectra (Figure S14A). DPP was inserted into the EV membrane, and Cy5.5 fluorophore was linked to PBP for imaging purposes (Figure 9A). The resulting MLB@PBP‐ADSC‐EVs retained the structural integrity and marker profile of native EVs and exhibited comparable zeta potential and particle size (Figure 9B–E). Biodistribution analysis showed that intravenously injected Cy5.5‐labeled MLB@PBP‐ADSC‐EVs accumulated more robustly in the lungs than MLB@ADSC‐EVs from 6 to 24 h post‐injection in CLP mice (6 × 10^12^ particles/kg). Importantly, quantitative analysis of major organs at 24 h further revealed that, compared with MLB@ADSC‐EVs, MLB@PBP‐ADSC‐EVs displayed a more pronounced increase in pulmonary signals, while increased uptake was also observed in the liver, spleen, and kidney, with no obvious increase in the heart (Figure 9F). Flow cytometry confirmed that MLB@PBP‐ADSC‐EVs retained structural stability at 4°C for at least 7 days (Figure S15A), and MLB release kinetics showed 85% cumulative release over 48 h (Figure 9I). Immunofluorescence confirmed enhanced colocalization of MLB@PBP‐ADSC‐EVs with pulmonary endothelial marker CD31 (Figure 9J), while in vitro uptake assays demonstrated increased internalization by CM‐treated HPMECs (Figure S15B). Together, these results support the preserved physicochemical properties of EVs, efficient MLB loading/release, and enhanced pulmonary endothelial targeting of MLB@PBP‐ADSC‐EVs.

**FIGURE 10:**
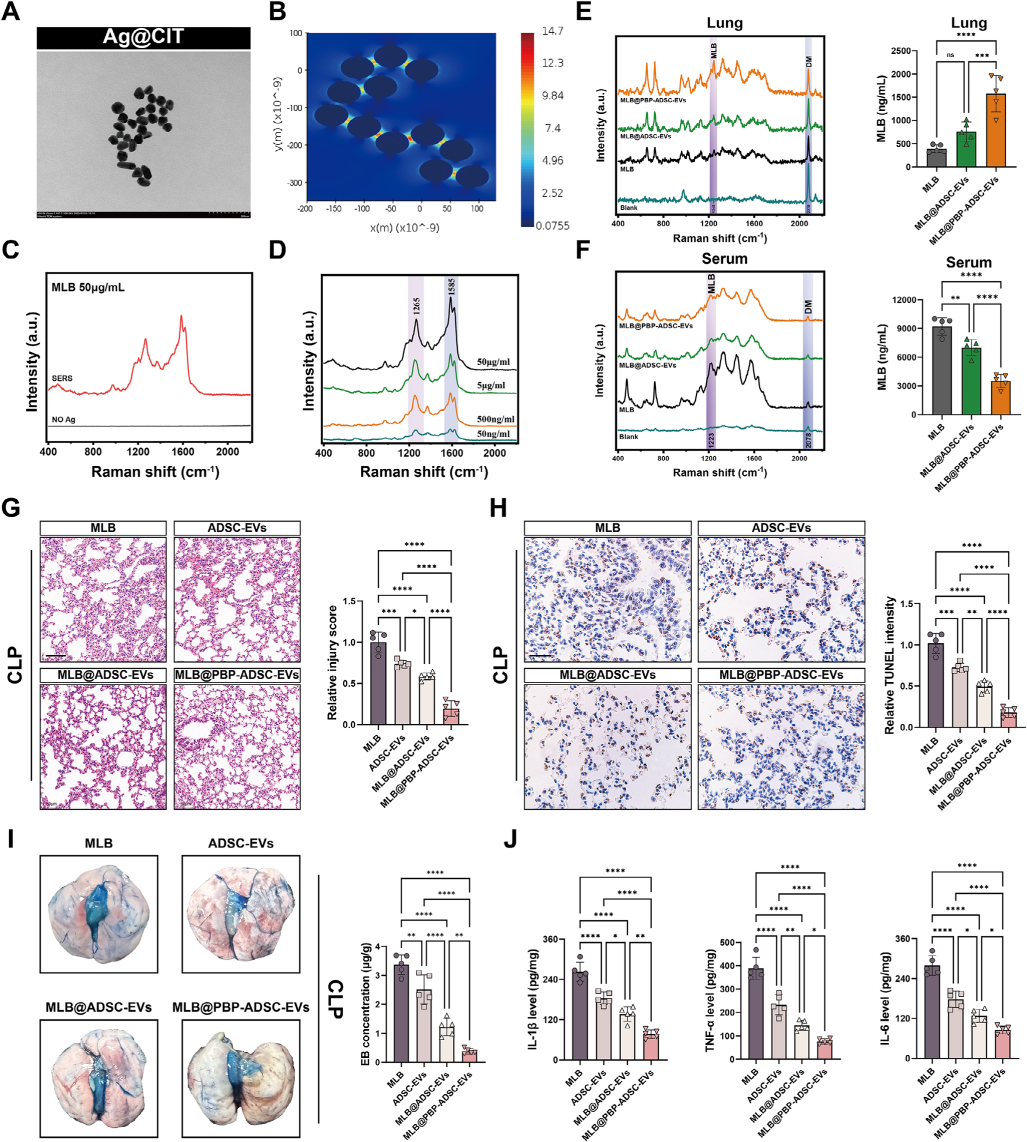
MLB@PBP‐ADSC‐EVs confer enhanced pulmonary protection in CLP mic‐induced septic mice. (A) TEM image of citrate‐coated silver nanoparticles (Ag@CIT) used as SERS substrates. Scale bar, 200 nm. (B) Finite‐difference time‐domain (FDTD) simulation depicting the electromagnetic field distribution around Ag@CIT nanoparticles. (C) Representative Raman spectra of MLB (50 µg/mL) acquired in the presence or absence of Ag@CIT substrates. (D) SERS spectra of MLB at various concentrations (50 ng/mL–50 µg/mL). (E,F) SERS‐based detection of MLB in lung tissue and serum at 24 h post‐injection of different formulations (*n *= 5). (G) Representative H&E staining of lung sections and quantification of lung injury scores (*n *= 5). Scale bar, 50 µm. (H) Representative TUNEL staining of lung sections and quantification of cell death (*n* = 5). Scale bar, 50 µm. (I) Representative images and quantitative analysis of Evans blue dye extravasation in lungs 24 h post‐treatment (*n* = 5). (J) ELISA‐based quantification of inflammatory cytokines IL‐1β, TNF‐α, and IL‐6 in lung tissues from mice (*n* = 5). Data are presented as mean ± SD. Statistical significance was determined by one‐way ANOVA followed by Tukey's post hoc test. *p < 0.05, **p < 0.01, ***p < 0.001, ****p < 0.0001, ns, not significant.

To evaluate the pulmonary delivery efficiency of MLB, we employed a SERS‐based strategy to quantify MLB distribution in lung tissue and serum. SERS is a highly sensitive and reproducible technique suitable for trace‐level compound detection [25]. Using previously established protocols [26], we successfully synthesized Ag@CIT SERS substrates (Figure 10A). Finite‐difference time‐domain (FDTD) simulations confirmed the presence of electromagnetic hotspots on the Ag@CIT surface, which facilitate signal enhancement (Figure 10B). The Ag@CIT substrates substantially amplified the Raman signal of MLB and enabled robust acquisition of its characteristic spectral fingerprint (Figure 10C), with stable readouts across replicates (Figure S16A). We next strengthened the quantitative performance of this SERS platform. A calibration curve was constructed using the intensity ratio of *I*_1265_/*I*_2078_ with deuterated methanol (DM) as an internal standard across MLB concentrations ranging from 1.5625 to 50 µg/mL (*R* ^2^ = 0.999) (Figure S16B). Based on the standard approach (*LOD* = 3.3*σ/k*; *LOQ *= 10*σ/k*), the detection sensitivity of this method was calculated as *LOD* = 40.9ng/mL and *LOQ* = 136.6 ng/mL (Figure 10D). To assess potential matrix interference, we acquired background Raman spectra from blank mouse serum and lung homogenates; neither sample displayed the characteristic MLB peak, indicating minimal endogenous spectral interference within the diagnostic region (Figure 10E,F). To provide orthogonal validation, we established a high‐performance liquid chromatography–mass spectrometry (HPLC–MS) calibration curve for MLB (*R* ^2^ = 0.9978) and quantified MLB levels in mouse serum. Paired comparison across six mice showed a strong concordance between SERS and HPLC–MS measurements (Pearson *R* = 0.945, *p* = 0.004) (Figure S16C,D), supporting the accuracy and reliability of SERS‐based in vivo quantification. We quantitatively assessed MLB levels in the lung and serum following intravenous administration. Notably, mice treated with MLB@PBP‐ADSC‐EVs exhibited significantly elevated MLB concentrations in lung tissue, accompanied by reduced serum levels, compared with free MLB or non‐targeted MLB@ADSC‐EVs (Figure 10E,F), indicating enhanced pulmonary delivery efficiency.

**FIGURE 11:**
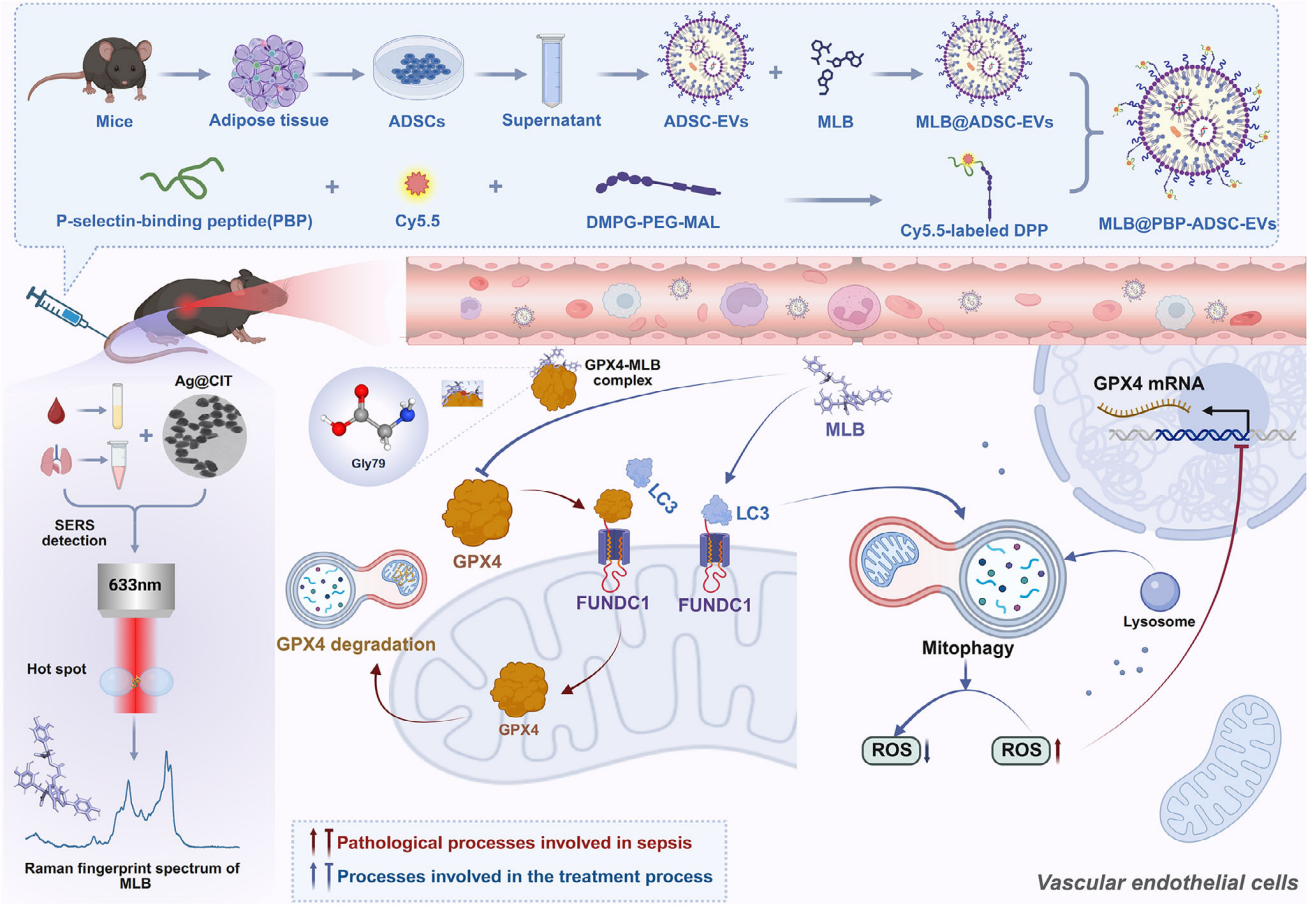
Schematic model illustrating the endothelial‐protective mechanism of MLB and its translational potential in targeted delivery and in vivo detection. MLB interacts with GPX4 to prevent its association with FUNDC1, thereby inhibiting GPX4 degradation through the mitophagy pathway. This dissociation enables FUNDC1 to recruit LC3 and restore mitophagic flux, reducing ROS accumulation in endothelial cells. The alleviation of oxidative stress relieves ROS‐mediated transcriptional repression of *GPX4*, resulting in increased GPX4 expression and mitigation of ferroptosis. For targeted delivery, MLB was encapsulated into ADSC‐EVs and further functionalized with Cy5.5‐labeled DPP to generate MLB@PBP‐ADSC‐EVs, enabling endothelial‐specific delivery in septic lungs. In parallel, SERS substrates based on Ag@CIT nanostructures were developed to detect MLB in biofluids and tissues with high sensitivity. These findings collectively highlight the therapeutic mechanism of MLB in SALI and support its translational application via targeted delivery and label‐free molecular detection.

We next investigated the therapeutic efficacy of MLB@PBP‐ADSC‐EVs in SALI. Histological analysis revealed no apparent toxic effects in the lung, heart, liver, or kidney after treatment in healthy mice (Figure S17A). In CLP mice, we compared MLB, ADSC‐EVs, MLB@ADSC‐EVs, and MLB@PBP‐ADSC‐EVs with identical MLB doses across all MLB‐containing groups and equal EV numbers among EV‐based formulations. Treatment with MLB@PBP‐ADSC‐EVs markedly attenuated pulmonary damage, as evidenced by reduced lung injury scores and fewer TUNEL‐positive cells, and conferred the greatest protection among all treatment groups compared with MLB, ADSC‐EVs, and MLB@ADSC‐EVs (Figure 10G,H). Consistently, MLB@PBP‐ADSC‐EVs most effectively reduced vascular permeability and proinflammatory cytokine levels (IL‐1β, TNF‐α, and IL‐6) in lung tissue (Figure 10I,J; Table S10) and suppressed ROS accumulation, as shown by diminished DHE fluorescence (Figure S18A). Collectively, these results demonstrate that MLB@PBP‐ADSC‐EVs provide superior lung protection over free MLB and non‐targeted EV formulations in SALI. Figure 11 illustrates the proposed mechanism by which MLB regulates endothelial ferroptosis in SALI, and outlines the strategy for targeted delivery and molecular detection.

## 3 Discussion

In the early stages of sepsis, excessive inflammatory responses contribute substantially to the onset of acute lung injury, representing a leading cause of mortality [27, 28, 29]. However, most current strategies fail to effectively prevent the rapid progression of SALI. Pulmonary microvascular endothelial damage has emerged as a central driver of early mortality in septic patients [30]. Accordingly, developing effective strategies to preserve endothelial integrity holds critical promise for improving clinical outcomes in sepsis [31]. A consistent reduction in GPX4 levels has been observed in septic lungs, where it is considered a major contributor to endothelial injury [7, 32]. While GPX4 is increasingly recognized for its protective role against multi‐organ dysfunction during sepsis, approaches to restore its expression and enzymatic activity remain insufficiently developed.

In this study, we first confirmed that GPX4 is significantly downregulated in pulmonary endothelial cells under septic conditions. Immunofluorescence analysis of human‐infected lung tissues, along with lung sections from CLP‐treated mice, confirmed this reduction. Although direct validation in SALI patient lung tissues was not feasible due to sample limitations, data from infection‐related human tissues provide meaningful translational relevance. To investigate the functional significance of endothelial GPX4 deficiency, we generated endothelial‐specific *Gpx4* knockout mice. Following CLP induction, these mice exhibited exacerbated pulmonary injury, highlighting the protective role of GPX4 in maintaining endothelial integrity during sepsis. These findings align with prior reports and reinforce the rationale for targeting ferroptosis, and specifically GPX4 preservation, as a therapeutic strategy in sepsis [32, 33].

To explore potential strategies for preserving GPX4 expression and activity, we conducted a high‐throughput screening of natural compounds targeting GPX4. Among the 19 candidate molecules with predicted binding capacity, Magnesium lithospermate B (MLB)—a major active constituent of *Salvia miltiorrhiza* aqueous extract—was selected based on its well‐documented anti‐inflammatory and antioxidant properties [34]. Previous studies have also demonstrated its protective effects against endothelial injury induced by hyperglycemia or inflammation [15, 35, 36]. To mimic the inflammatory milieu of sepsis, we employed an in vitro model in which HPMECs were treated with CM from LPS‐stimulated macrophages, as described previously [6, 7]. MLB treatment markedly reduced lipid peroxidation in HPMECs and was found to directly bind GPX4. Notably, MLB not only reversed the CM‐induced downregulation of GPX4 protein but also enhanced its enzymatic activity. GPX4 detoxifies lipid hydroperoxides via its catalytic selenocysteine residue at position 46 (Sec46). Using DFT calculations, we showed that MLB engages in electrostatic interactions within the Sec46 region, suggesting a potential mechanism by which MLB protects GPX4 from oxidative inactivation through noncovalent or orbital‐level interactions. However, the precise nature of this interaction remains to be clarified.

Immune cells, including macrophages and neutrophils, also play essential roles in SALI [37]. In our in vivo models, MLB not only ameliorated CLP‐ and LPS‐induced lung damage, preserved endothelial barrier function, and attenuated ferroptosis, but was also associated with a modest reduction in pulmonary infiltration of F4/80^+^ macrophages and Ly6G^+^ neutrophils. We speculate that this effect on immune cell recruitment is likely indirect. During SALI, mitochondrial dysfunction and ferroptotic injury can increase ROS production and promote the release of damage‐associated molecular patterns (DAMPs), while concomitant endothelial barrier disruption facilitates leukocyte extravasation, together promoting immune cell accumulation in the lung. By stabilizing GPX4, restoring mitochondrial homeostasis, lowering ROS and inflammatory cytokine levels, and reinforcing barrier integrity, MLB may collectively contribute to a less permissive inflammatory microenvironment and thereby secondarily limit immune cell infiltration. These observations raise the possibility that, beyond direct endothelial protection, MLB might also modulate local inflammatory responses. However, the precise effects of MLB on immune cell activation and function will require more detailed investigation in future studies.

Since our preliminary data confirmed the direct binding of MLB to the GPX4 protein, we first focused on the protein degradation pathways. Previous studies have established that GPX4 is primarily degraded through both proteasomal and lysosomal pathways [16, 17], and our prior work also observed that GPX4 undergoes ubiquitination during sepsis‐induced lung injury [7]. In the present study, CHX‐chase assays revealed that both MG132 and BafA1 delayed GPX4 turnover, supporting the involvement of both proteasomal and lysosomal pathways in CM‐treated endothelial cells. Notably, co‐treatment with MLB and MG132 further increased GPX4 levels, whereas MLB showed no additive effect when combined with BafA1 or CQ. Together with the unchanged ubiquitination status of GPX4, these pharmacological patterns suggest that MLB may preferentially limit lysosome‐associated GPX4 degradation, rather than directly blocking proteasomal turnover. While we systematically evaluated lysosome‐mediated degradation using chemical inhibitors, subcellular fractionation, and autophagy flux analysis, future studies incorporating ATG5/ATG7 knockdown or knockout models will be essential to determine the autophagy dependence of GPX4 turnover.

To further elucidate the underlying mechanism, we conducted single‐cell transcriptomic enrichment analysis of pulmonary endothelial cells stratified by *GPX4* expression levels. The results revealed a significant association between GPX4 expression and mitophagy‐related pathways. Consistently, subcellular fractionation indicated that GPX4 is regulated in a compartment‐restricted manner under CM stress, with differential distribution between mitochondrial and cytosolic pools. Notably, MLB reduced the mitochondrial localization of GPX4 under CM stress. Previous studies have shown that FUNDC1, an outer mitochondrial membrane protein, directly interacts with GPX4, promoting its translocation into mitochondria and subsequent degradation via mitophagy [11]. This interaction also impairs the binding of FUNDC1 to LC3B, thereby reducing mitophagy efficiency and leading to excessive ROS accumulation and cellular injury. Such crosstalk between GPX4 and FUNDC1 has been identified as a critical node influencing mitochondrial homeostasis under inflammatory stress. We found that MLB markedly ameliorated sepsis‐induced mitochondrial dysfunction and enhanced mitophagic activity, in line with previous reports describing its role in maintaining mitochondrial homeostasis [13, 38]. Mechanistically, MLB disrupted the GPX4‐FUNDC1 interaction while concurrently promoting the association between FUNDC1 and LC3B and shifting GPX4 from the mitochondrial to the cytosolic compartment. CETSA further confirmed that MLB does not directly bind to FUNDC1, suggesting that its effect is explicitly mediated through GPX4. Taken together, these findings support a dual mechanism by which MLB protects endothelial cells. By selectively binding to GPX4, MLB interferes with its association with FUNDC1, thereby stabilizing GPX4, favoring its cytosolic localization, and enhancing its enzymatic activity to suppress ferroptosis. Simultaneously, the dissociation of GPX4 from FUNDC1 facilitates the latter's engagement with LC3B, restoring mitophagic flux. This functional uncoupling of GPX4 and FUNDC1 highlights MLB as a modulator of both ferroptosis and mitochondrial quality control, presenting a promising therapeutic strategy for preserving endothelial integrity in sepsis.

In subsequent experiments, we further investigated the regulatory role of FUNDC1 on GPX4 protein expression. Unexpectedly, neither knockdown nor overexpression of *FUNDC1* altered GPX4 protein levels in CM‐treated HPMECs. However, subcellular fractionation revealed that FUNDC1 promotes the mitochondrial localization of GPX4 under CM stress, which is consistent with the proposed FUNDC1‐dependent, mitochondria‐linked turnover of GPX4. In addition, given that mitophagy mitigates intracellular ROS accumulation, and elevated ROS levels are known to suppress *GPX4* transcriptionally [18], we speculate that the relatively stable net GPX4 protein abundance upon FUNDC1 manipulation under CM stress may reflect counterbalancing effects between ROS‐sensitive transcriptional regulation and post‐translational stabilization of GPX4. To test this hypothesis, we treated HPMECs with exogenous H_2_O_2_ in the presence of MLB. Notably, H_2_O_2_ completely abolished the MLB‐induced increase in *GPX4* mRNA levels and partially attenuated the upregulation of GPX4 protein expression. These results further support the notion that MLB modulates GPX4 expression via both transcriptional and post‐translational mechanisms. Moreover, CM stimulation elevated apoptosis markers (cleaved caspase‐3/9) and pyroptosis markers (GSDMD, cleaved caspase‐1) in HPMECs, while MLB treatment significantly attenuated these changes. However, co‐treatment with H_2_O_2_ largely abolished MLB's protective effects. These observations suggest that ROS regulation is closely linked to the broader cytoprotective actions of MLB, although the precise contribution of apoptosis and pyroptosis to SALI, and how MLB differentially modulates these death programs, will require more detailed investigation in future studies. Collectively, our findings reveal a multifaceted protective role of MLB in regulating GPX4 through: (1) enhancing GPX4 enzymatic activity; (2) shielding GPX4 from mitophagy‐dependent degradation; and (3) disrupting the inhibitory interaction between GPX4 and FUNDC1, thereby restoring mitophagic flux, reducing ROS accumulation, and relieving ROS‐mediated suppression of *GPX4* transcription. This triple regulatory mechanism underscores the pivotal role of MLB in sustaining GPX4 levels and promoting ferroptosis resistance during sepsis.

To further elucidate the molecular basis of the MLB‐GPX4 interaction, we conducted structure‐based molecular docking, which revealed that MLB forms hydrogen bonds with GPX4 at Gly79 and Asn109. Among these, Gly79 was experimentally validated as a critical residue mediating the protective effect of MLB. Moreover, RMSF analysis from molecular dynamics simulations demonstrated that the region surrounding residue 75 of GPX4 exhibits low structural flexibility, suggesting it may serve as a favorable binding hotspot for small‐molecule ligands. Notably, Gly79 is located within this relatively rigid region, providing indirect structural support for its functional role in MLB binding. Additionally, Gly79 is located near the catalytic Sec46 residue of GPX4. Integrating this spatial insight with our DFT calculations, we propose that MLB binding to Gly79 may also exert an electronic influence on Sec46, thereby enhancing GPX4 enzymatic activity. Supporting this model, the Gly79 mutation not only disrupted MLB binding but also significantly reduced its ability to boost GPX4 activity. Together, these findings identify Gly79 as a dual regulatory site mediating both the structural and catalytic modulation of GPX4 by MLB. This previously unrecognized allosteric mechanism expands our understanding of the structure–function relationship underlying MLB's cytoprotective effects.

The reciprocal inhibitory interaction between GPX4 and FUNDC1 may limit the effectiveness of current strategies aimed at restoring mitochondrial homeostasis. MitoQ has been shown to alleviate mitochondrial oxidative stress, inhibit apoptosis, and preserve intracellular homeostasis [39, 40]. Preclinical studies have demonstrated that MitoQ enhances endothelial barrier integrity in sepsis models, and its vascular‐protective effects are currently under clinical investigation [41]. Moreover, MitoQ has been reported to promote mitophagy, contributing to sustained mitochondrial function and redox stability [42]. In this study, the combination of MLB and MitoQ conferred superior protection against pulmonary injury compared to either agent alone. We hypothesize that this enhanced efficacy arises from a synergistic mechanism: MLB inhibits mitophagy‐dependent degradation of GPX4, thereby offsetting the potential GPX4 depletion associated with MitoQ‐induced mitophagic activation. This coordinated modulation preserves GPX4 activity while simultaneously optimizing mitochondrial quality control, resulting in improved mitochondrial homeostasis and amplified therapeutic benefit. These findings reveal a previously unrecognized therapeutic interplay between a mitophagy‐enhancing agent and a ferroptosis‐inhibiting compound. This combinatorial approach offers a novel strategy to restore mitochondrial redox balance and reinforce endothelial protection in sepsis.

P‐selectin, a member of the selectin family of transmembrane adhesion molecules, is predominantly expressed on vascular endothelial cells. While its basal expression is low under resting conditions, it is rapidly upregulated in response to inflammatory cytokines, thrombin, or histamine, thereby promoting leukocyte adhesion and exacerbating local inflammation [22]. Consistent with previous studies [23, 24], our data revealed that P‐selectin was markedly elevated in pulmonary endothelium during SALI, supporting its feasibility as a target for endothelial‐directed delivery. PBP, a small peptide ligand with high affinity for P‐selectin, offers advantages over traditional antibodies or antagonists—including low synthesis cost, facile chemical modification, and ease of conjugation to nanocarriers—making it an attractive candidate for engineering targeted extracellular vesicles (EVs) [43]. ADSCs, which are abundant and readily isolatable, have shown increasing translational value in mitigating sepsis‐induced organ dysfunction [44]. Notably, ADSC‐EVs have been reported to modulate mitochondrial function and inhibit programmed cell death, highlighting their potential as both therapeutic agents and drug delivery vehicles [45, 46, 47]. Thus, we developed PBP‐engineered ADSC‐EVs encapsulating MLB (MLB@PBP‐ADSC‐EVs) to achieve endothelial‐specific delivery of MLB for treating SALI. Our results confirmed that this engineering strategy significantly enhanced pulmonary targeting efficiency and improved therapeutic outcomes. Under conditions where the MLB dose was identical across all MLB‐containing groups, and the EV number was matched among EV‐based formulations, MLB@PBP‐ADSC‐EVs consistently exhibited the most pronounced lung‐protective effects compared with MLB, ADSC‐EVs, and MLB@ADSC‐EVs. These stepwise differences indicate that loading MLB into a P‐selectin‐targeted EV delivery platform confers additional therapeutic benefit beyond that achieved with non‐targeted formulations or free drug administration.

Notably, biodistribution analyses revealed that, in addition to robust lung enrichment, MLB@PBP‐ADSC‐EVs also exhibited increased accumulation in other organs, including the liver, spleen, and kidney. This pattern is consistent with the systemic inflammatory and endothelial‐activation context of sepsis, in which P‐selectin may be inducibly upregulated across multiple vascular beds, potentially increasing the overall vascular association of PBP‐modified EVs. Notably, the primary objective of this study remains to optimize targeted delivery and therapeutic benefit in SALI. Given the highly vascularized architecture of the lung and its full exposure to circulating EVs, PBP modification may confer a favorable targeting advantage for pulmonary applications. Meanwhile, the increased uptake in other organs suggests that this platform may warrant further exploration for broader prevention or treatment of sepsis‐related multi‐organ injury. This study represents the first application of P‐selectin‐targeted EVs in a septic context, underscoring their promise as a targeted drug‐delivery strategy for systemic inflammatory diseases.

The limited systemic distribution of MLB following administration presents a key technical barrier to accurately assessing delivery efficiency. To address this, we employed SERS, a highly sensitive, rapid, and non‐destructive molecular detection technology capable of enabling trace‐level analysis in complex biological matrices [25]. SERS amplifies Raman signals through plasmonic enhancement when molecules adsorb onto metallic nanostructures and has been increasingly applied for profiling drug biodistribution and informing personalized dosing strategies [26]. We synthesized a high‐performance SERS substrate and successfully captured the characteristic Raman fingerprint spectrum of MLB for the first time. This platform enabled sensitive detection of MLB in both lung homogenates and serum at the nanogram scale. Using this approach, we found that MLB@PBP‐ADSC‐EVs significantly increased MLB accumulation in lung tissue compared to non‐targeted delivery systems. Together, these findings demonstrate the feasibility of using SERS for quantitative MLB detection in vivo and underscore the potential of PBP‐modified ADSC‐EVs for enhancing endothelial‐specific delivery. This work provides a promising foundation for the development of personalized monitoring and precision delivery strategies for small‐molecule therapeutics in sepsis.

In this study, we confirm that MLB confers pulmonary protection in a mouse model of SALI. Nonetheless, several limitations warrant consideration. First, although MLB significantly ameliorated CLP‐induced lung injury in mice, its efficacy has yet to be validated in large animal models that more closely mimic clinical sepsis. Second, while molecular docking, site‐directed mutagenesis, and functional assays collectively revealed Gly79 as a critical residue mediating MLB‐GPX4 binding, definitive structural evidence is still lacking. Third, our investigation focused primarily on pulmonary microvascular endothelial cells, leaving the potential effects of MLB on other cell types involved in SALI pathogenesis unexplored. Finally, although we delineated the role of MLB in modulating the GPX4‐FUNDC1 interaction, it remains to be determined whether MLB also affects additional regulators of mitochondrial homeostasis or ferroptosis.

## 4 Conclusion

In summary, our findings identify the natural small molecule MLB as a novel modulator of GPX4, capable of disrupting its interaction with FUNDC1 to prevent mitophagy‐dependent GPX4 degradation, thereby restoring FUNDC1‐mediated mitophagic flux and mitigating endothelial ferroptosis during sepsis. Furthermore, by engineering MLB@PBP‐ADSC‐EVs, we achieved targeted delivery of MLB to inflamed endothelium, substantially enhancing its therapeutic efficacy in SALI. Additionally, we applied SERS‐based molecular detection to enable quantitative monitoring of in vivo MLB distribution. These findings not only clarify MLB's protective mechanism in SALI but also establish a targeted delivery and detection strategy that supports its clinical translation.

## 5 Experimental Section

### 5.1 Reagent

Mouse ELISA kits for Angpt2, IL‐1β, IL‐6, and TNF‐α were purchased from Jingkang Bio (Shanghai, China). Magnesium lithospermate B (HY‐126415), Ferrostatin‐1 (HY‐100579), Chloroquine (HY‐17589A), MG‐132 (HY‐13259), and Cy5.5 (HY‐D0924) were obtained from MedChemExpress (NJ, USA). Lipopolysaccharide (LPS, L2630) and phorbol 12‐myristate 13‐acetate (PMA, P1585) were sourced from Sigma‐Aldrich (MO, USA). Lipofectamine 3000 and MitoTracker Red were purchased from Invitrogen (MD, USA). Phalloidin (MX4405) was obtained from Mkbio (Shanghai, China). Propidium iodide (PI), DAPI, Hoechst 33342, JC‐1, DHE, MitoSOX Red, and ROS detection kits were purchased from Beyotime Biotechnology (Shanghai, China). Zombie NIR fixable viability dye was obtained from BioLegend (CA, USA). Cell Counting Kit‐8 (CCK‐8) and Liperfluo were purchased from DOJINDO Laboratories (Kumamoto, Japan). AceQ qPCR SYBR Green Master Mix (Q111‐03) was provided by Vazyme (Nanjing, China), and the First Strand cDNA Synthesis Kit (FSK‐101) was obtained from TOYOBO (Osaka, Japan). Primer sequences used for quantitative real‐time PCR are listed in Table S11.

### 5.2 Virtual Screening and Molecular Docking

Virtual screening was conducted using a natural compound library (L6000, Topscience; 4320 compounds). 2D structures in SDF format were imported into the Schrödinger Suite for ligand preparation. The LigPrep module was used to generate 3D conformers with appropriate protonation states and tautomers predicted by Epik at pH 7 ± 2. All structures were minimized using the OPLS_4 force field. Molecular docking was carried out in a hierarchical manner: initial screening by high‐throughput virtual screening (HTVS, top 50%), followed by Standard Precision (SP, top 20%), and Extra Precision (XP) docking for the top 100 compounds. Binding free energies of the XP‐docked poses were further rescored using the MM‐GBSA module. Compounds with MM‐GBSA Δ*G* bind scores < −50 kcal/mol were considered for further analysis after removing structural duplicates.

### 5.3 Immunofluorescence Staining of Frozen Human Lung Tissues

Frozen human lung tissues were collected from patients with or without pulmonary infection admitted to the Second Affiliated Hospital of Harbin Medical University. All procedures involving human specimens were approved by the Institutional Ethics Committee (Approval No.: YJSKY2022‐138), and written informed consent was obtained from all participants or their legal representatives. Tissues were embedded in OCT compound and cryosectioned at a thickness of 7 µm using a Leica CM1950 cryostat.

Sections were air‐dried at room temperature for 30 min, fixed in cold acetone for 10 min, and rinsed three times with PBS. After blocking with 5% bovine serum albumin for 1 h at room temperature, sections were incubated overnight at 4°C with primary antibodies against CD31 and GPX4. The following day, after thorough washing, sections were incubated with Alexa Fluor‐conjugated secondary antibodies for 1 h at room temperature. Nuclei were counterstained with DAPI, and slides were mounted using antifade mounting medium. Fluorescence images were acquired using a Zeiss LSM 880 confocal microscope.

### 5.4 Animal Model of Sepsis‐Associated Lung Injury (SALI)

Male C57BL/6 mice (8—10 weeks old, 20–25 g) were obtained from the Animal Experiment Center of the Second Affiliated Hospital of Harbin Medical University. *Gpx4^f/f^* and *Gpx4^f/f^Cdh5^Cre^* mice, both on a C57BL/6 background, were purchased from Cyagen Biosciences. All animal procedures were approved by the Ethics Committee of the Second Affiliated Hospital of Harbin Medical University (Approval No.: SYDW2021‐087) and conducted in accordance with the ARRIVE guidelines.

A mouse model of SALI was established using the cecal ligation and puncture (CLP) procedure with minor modifications [48]. Briefly, mice were anesthetized with sevoflurane (induction: 5%, maintenance: 2%–3%), and a midline laparotomy (∼1 cm) was performed to expose the cecum. The midpoint of the cecum was ligated using a 4‐0 silk suture, and the ligated segment was punctured bilaterally with a 20‐gauge needle. A small amount of fecal material was gently extruded to ensure patency. The cecum was returned to the peritoneal cavity, and the abdominal wall was closed in layers using 4‐0 silk sutures. Postoperative fluid resuscitation was performed with sterile 0.9% saline (50 µL/g body weight, subcutaneous) immediately after surgery. Mice were maintained on a warming pad (30°C ± 2°C) until full recovery. Sham‐operated mice underwent identical procedures without cecal ligation or puncture.

### 5.5 Randomization and Blinding

Animals were randomly assigned to experimental groups using a computer‐generated randomization sequence by an investigator not involved in subsequent outcome assessment or data analysis. Due to the nature of the surgical procedure, the operator could not be blinded to CLP versus sham surgery; however, the operator was blinded to treatment allocation within CLP groups (vehicle vs. MLB) via coded injections prepared by an independent investigator. Blinding was maintained during sample processing (sectioning, staining, protein extraction) and quantitative analysis/statistics until completion of data validation.

### 5.6 Prespecified Exclusion Criteria

Exclusion criteria were defined a priori. Animals were excluded if they met any of the following: (1) death during anesthesia or within 2 h after surgery (procedure/anesthesia‐related), (2) technical failure of CLP (e.g., incomplete ligation/puncture confirmed intraoperatively), or (3) excessive intraoperative bleeding requiring premature termination of the procedure.

### 5.7 Compound Administration

Magnesium lithospermate B (MLB) was administered by tail‐vein injection at 15 mg/kg (low dose, MLB‐L) or 30 mg/kg (high dose, MLB‐H). The dosing schedule consisted of one pretreatment dose at 24 h before CLP and one post‐treatment dose at 2 h after CLP, aiming to ensure target engagement before the septic insult and to maintain drug exposure during the early phase of sepsis when endothelial barrier disruption develops. Dose selection was guided by a previous study [15] and our preliminary dose‐response study in CLP mice. Mitoquinone (MitoQ) was administered intravenously at 3 mg/kg via tail vein injection, 30 min before CLP surgery, according to previously published protocols [41].

### 5.8 Preparation of Macrophage‐Conditioned Medium

THP‐1 monocytes were differentiated into M0 macrophages by treatment with phorbol 12‐myristate 13‐acetate (PMA, 50 ng/mL) for 24 h. After differentiation, cells were stimulated with lipopolysaccharide (LPS, 1 µg/mL) for an additional 24 h. The culture supernatant was then harvested and centrifuged at 2000 rpm for 10 min to remove cellular debris, followed by filtration through a 0.22‐µm pore‐size membrane to obtain cell‐free conditioned medium.

### 5.9 Cell Culture and Experimental Grouping

Human THP‐1 monocytes were maintained in RPMI‐1640 medium supplemented with 10% fetal bovine serum (FBS) and subcultured routinely at appropriate densities in fresh medium. Human pulmonary microvascular endothelial cells (HPMECs) were cultured in endothelial cell medium (ECM) supplemented with 5% FBS and 1% endothelial cell growth supplement. For subculture, adherent cells were rinsed with sterile phosphate‐buffered saline (PBS), digested with trypsin for 2 min, and neutralized with complete medium. Single‐cell suspensions were obtained by gentle pipetting and seeded into new culture flasks for expansion.

To evaluate the effects of magnesium lithospermate B (MLB) on conditioned medium (CM)‐treated HPMECs, cells were divided into four groups: (1) Control group, HPMECs cultured under standard conditions without any treatment; (2) CM group, culture medium was replaced with macrophage‐derived CM for 24 h prior to analysis; (3) CM + MLB group, HPMECs were pretreated with MLB (100 µm) for 2 h followed by co‐culture with CM for 24 h; and (4) CM + Fer‐1 group, HPMECs were pretreated with Ferrostatin‐1 for 2 h prior to co‐culture with CM for 24 h.

### 5.10 Histological Assessment of Lung Injury

Paraffin‐embedded lung tissue sections 4 µm) were deparaffinized in xylene and rehydrated through a graded ethanol series. Sections were stained with hematoxylin (5 min), rinsed in water, and counterstained with eosin (10 min), followed by a 15‐min rinse. After dehydration and clearing, the slides were mounted using neutral resin. Lung histopathological changes were evaluated under a light microscope in a blinded manner by two independent investigators. Lung injury was scored using a previously established semi‐quantitative system assessing alveolar congestion, hemorrhage, neutrophil infiltration, and alveolar wall thickening [49].

### 5.11 Evans Blue Extravasation Assay

Pulmonary vascular permeability was assessed using the Evans Blue dye extravasation method. Mice were intravenously injected with 0.5% Evans Blue dye (2 mL/kg) via the tail vein. Thirty minutes after injection, mice were euthanized, and lung tissues were harvested and homogenized in 1 mL of 50% trichloroacetic acid (prepared in PBS). The homogenates were centrifuged at 10 000 × *g* for 20 min, and the resulting supernatants were diluted 1:4 with absolute ethanol. The absorbance of each sample was measured at 620 nm using a spectrophotometer, and Evans Blue concentrations were determined based on a standard calibration curve.

### 5.12 Measurement of Lung W/D Ratio

To assess pulmonary edema, the left lung was excised immediately after euthanasia, gently blotted to remove surface moisture, and weighed to obtain the wet weight. The tissues were then placed in an oven at 60°C and dried for 72 h to determine the dry weight. The lung water content was calculated using the formula: W/D ratio = (wet weight/dry weight) × 100%.

### 5.13 Western Blot Analysis

Protein samples were separated by SDS‐PAGE and transferred onto 0.22 µm PVDF membranes at 300 mA for 2 h under ice bath conditions. Membranes were blocked with 5% non‐fat milk for 1 h and incubated overnight at 4°C with the following primary antibodies: ZO‐1 (1:2000, AF5145, Affinity, OH, USA), VE‐Cadherin (1:2000, AF6265, Affinity, OA, USA), occludin (1:1000, 91 131, Cell Signaling Technology, MA, USA), β‐actin (1:100 000, AC026, ABclonal, Wuhan, China), SLC7A11 (1:10 000, ab175186, Abcam, UK), GPX4 (1:10 000, ab125066, Abcam, UK), Calnexin (1:2000, AF5362, Affinity, OA, USA), TSG101 (1:3000, DF8427, Affinity, OA, USA), CD81 (1:2000, DF2306, Affinity, OA, USA), CD63 (1:1000, AF5117, Affinity, OA, USA), APO B48 (1:10 000, ab139401, Abcam, UK), TOM20 (1:5000, 11802‐1‐AP, Proteintech, IL, USA), LC3B (1:4000, 14 600‐1‐AP, Proteintech, IL, USA), Cleaved Caspase‐9 (1:1000, 9661, Cell Signaling Technology, MA, USA), Cleaved Caspase‐3 (1:1000, 9505, Cell Signaling Technology, MA, USA), Cleaved Caspase‐1 (1:1000, 4199, Cell Signaling Technology, MA, USA) and Cleaved Gasdermin D (1:1000, 36 425, Cell Signaling Technology, MA, USA). After washing with TBST, membranes were incubated with secondary antibodies (1:10 000, ab6712, Abcam, UK; 1:100 000, AS003, ABclonal, China) for 1 h at room temperature. Bands were visualized using enhanced chemiluminescence (ECL) and imaged accordingly.

### 5.14 Flow Cytometric Analysis of Cell Viability

Endothelial cell viability was evaluated using the Zombie NIR fixable viability dye (BioLegend). Cells were harvested by trypsinization, washed with phosphate‐buffered saline (PBS), and resuspended at a density of 1–10 × 10^6^ cells per 100 µL in freshly prepared Zombie NIR staining solution. Cells were incubated in the dark at room temperature for 30 min. The staining reaction was terminated by adding 10% bovine serum albumin (BSA), and the cells were immediately subjected to flow cytometric analysis using a Micro Plus nanoflow cytometer (Apogee) with emission detection at 746 nm.

### 5.15 Flow Cytometric Detection of Lipid Peroxidation

Lipid peroxidation in endothelial cells was assessed using the fluorescent probe C11‐BODIPY 581/591 (Thermo Fisher Scientific). Cells were incubated with 10 µm C11‐BODIPY in complete medium at 37°C for 30 min in the dark, washed three times with PBS, harvested using trypsinization, and resuspended in PBS. Fluorescence intensity shifts were immediately analyzed at 488 nm using a Micro Plus nanoflow cytometer (Apogee).

### 5.16 Liperfluo Staining

Intracellular lipid peroxidation was measured using the Liperfluo fluorescent probe (Dojindo). After removing the culture medium, cells were washed once with serum‐free medium and incubated with Liperfluo working solution at 37°C for 30 min. Following two gentle washes with PBS, cells were immediately imaged using a laser scanning confocal microscope (Zeiss LSM series).

### 5.17 JC‐1 Staining

Mitochondrial membrane potential (Δ*Ψm*) was assessed using JC‐1 staining (Beyotime). The JC‐1 working solution was prepared by diluting the stock reagent (1:200) in the JC‐1 staining buffer. Cells were incubated with the working solution at 37°C for 20 min, followed by gentle washing with the buffer. Fluorescence images were acquired using a confocal microscope to evaluate the red/green fluorescence ratio, which reflected changes in mitochondrial polarization status.

### 5.18 Cellular Thermal Shift Assay

Briefly, cell lysates treated with vehicle or MLB were aliquoted into equal volumes and subjected to a temperature gradient ranging from 37°C to 64°C. Following heating, samples underwent two cycles of rapid freeze–thaw using liquid nitrogen and were centrifuged to remove precipitated proteins. The supernatants containing thermally stable proteins were collected and analyzed by western blot to evaluate GPX4 stability under different thermal conditions [50].

### 5.19 Molecular Dynamics Simulations

Molecular dynamics simulations were performed using GROMACS v2022.3. The force field parameters for MLB were generated using AmberTools22 with the general amber force field (GAFF), and RESP atomic charges were derived from electrostatic potentials calculated by Gaussian 16 W. The protein–ligand complex was parameterized using the Amber99SB‐ILDN force field and solvated in a TIP3P explicit water model in a cubic box with periodic boundary conditions. Counterions (Na^+^) were added to neutralize the system. After steepest descent energy minimization, the system was equilibrated under NVT and NPT ensembles for 100 ps each. The production run was conducted for 100 ns with a 2‐fs time step at 300 K and 1 bar using the v‐rescale thermostat and Parrinello–Rahman barostat. Trajectory analyses, including root mean square deviation (RMSD) and fluctuation (RMSF), were performed using GROMACS utilities [51].

### 5.20 Cell Transfection

Small interfering RNAs (siRNAs) targeting genes of interest were synthesized by GenePharma and transfected using Lipofectamine 2000 (Invitrogen) according to the manufacturer's instructions. HPMECs at 40%–60% confluence were transfected with siRNA‐lipid complexes prepared in Opti‐MEM and incubated for 4–6 h before replacement with fresh complete medium.

For overexpression experiments, plasmids encoding human *GPX4* wild‐type (*GPX4‐WT*), G79S mutant (*GPX4‐G79S*), and N109S mutant (*GPX4‐N109S*) (obtained from the Shanghai Institute of Nutrition) were transfected into HPMECs using Lipofectamine 3000 (Invitrogen). Briefly, 0.8 µg of plasmid DNA and 2 µL of Lipofectamine 3000 reagent were each diluted in 50 µL of Opti‐MEM, mixed, and incubated for 20 min at room temperature before addition to cells. Transfected cells were cultured under standard conditions for 24–48 h prior to downstream assays.

### 5.21 AAV‐Mediated GPX4 Site‐Specific Mutation

To evaluate the functional role of the GPX4 point mutation in SALI, recombinant adeno‐associated virus serotype 2/9 (AAV2/9) vectors encoding either wild‐type GPX4 (GPX4‐WT) or the G79S mutant (GPX4‐G79S) were generated and purified. C57BL/6 mice (6–8 weeks old) received a single intravenous injection of 100 µL AAV suspension containing 1 × 10^1^ ^2^ vector genomes (vg) via the tail vein. After a 3‐week expression period to ensure stable systemic transgene expression, mice were subjected to CLP surgery to induce polymicrobial sepsis, following established procedures.

### 5.22 Isolation and Characterization of Primary Mouse Adipose‐Derived Stem Cells

Primary mouse adipose‐derived stem cells (ADSCs) were isolated from the inguinal white adipose tissues of 4–6‐week‐old C57BL/6 mice. All surgical instruments and consumables were sterilized under ultraviolet light for 30 min before use. Collagenase type I was prepared at 2 mg/mL in 10 mm HEPES‐buffered PBS and sterilized by filtration through a 0.22 µm membrane. Mice were euthanized by cervical dislocation and disinfected by immersion in 75% ethanol for 5–10 min. Inguinal fat pads were aseptically harvested and rinsed in sterile PBS. Tissues were finely minced and incubated in freshly prepared collagenase solution at 37°C for 30–60 min until fully digested. The enzymatic reaction was terminated by adding an equal volume of complete medium (containing 10% fetal bovine serum and 1% penicillin‐streptomycin). The cell suspension was centrifuged at 2000 rpm for 10 min at 4°C. The resulting pellet was resuspended in 5 mL of complete medium, filtered through a 40 µm strainer, and centrifuged again under identical conditions. The final cell pellet was seeded into a T25 culture flask in fresh complete medium. After 4 h of incubation, non‐adherent cells were removed, and adherent cells were washed 3–5 times with PBS to eliminate debris. Cells were cultured at 37°C in a humidified 5% CO_2_ incubator. Phenotypic characterization was performed by flow cytometry. ADSCs were positive for CD29, CD44, CD73, and CD105 (>90%) and negative for CD34, CD45, and CD11b (<5%). Multipotency was validated by differentiation into adipogenic and osteogenic lineages using standard induction protocols.

### 5.23 Isolation and Engineering of ADSC‐Derived Extracellular Vesicles

Extracellular vesicles (EVs) were isolated from the culture supernatants of ADSCs via differential centrifugation. Conditioned media were sequentially centrifuged at 2000 × g for 20 min and 10 000 × g for 30 min at 4°C to remove cells and debris. Supernatants were then ultracentrifuged at 120 000 × g for 70 min. Pelleted EVs were washed once with PBS and re‐ultracentrifuged. The final EV pellet was resuspended in PBS, quantified using a BCA protein assay, and stored at −80°C until further use.

### 5.24 MLB Loading and PBP Surface Modification

To generate MLB‐loaded EVs (MLB@EVs), 100 µg of purified EVs were incubated with Magnesium lithospermate B (MLB) at a mass ratio of 1:5 (EVs: MLB) at 37°C for 1 h. Free MLB was removed by ultracentrifugation followed by one wash with PBS. The EV pellets were then resuspended in PBS and subjected to ultrasonication to disrupt the vesicle membrane and release encapsulated MLB. The MLB concentration in the lysate was determined by UV spectrophotometry. Encapsulation efficiency (*EE*%) was calculated as follows: *EE*% = (mass of loaded MLB / mass of input MLB) × 100%. Surface modification with P‐selectin‐binding peptide (PBP; CDAEWVDVS) was achieved through hydrophobic insertion. Specifically, DMPE‐PEG5000‐MAL was conjugated with PBP to form DMPE‐PEG‐PBP (DPP), which was incubated with MLB@EVs at 25°C for 30 min. Cy5.5‐labeled DPP was used to confirm membrane modification efficiency (>90%) by flow cytometry [43]. In vitro release kinetics were assessed by placing MLB@EVs in a dialysis bag (MWCO: 10 kDa) and dialyzing in PBS containing 0.5% Tween‐80 at 37°C with shaking (100 rpm). At predetermined time points (0–72 h), samples were collected and replaced with fresh buffer. MLB concentration was determined based on absorbance at 284 nm, and cumulative release curves were generated accordingly.

### 5.25 Preparation of Ag@CIT Nanoparticles for SERS Substrates

Silver nanoparticles (AgNPs) were synthesized with slight modifications. Briefly, 200 mL of deionized water was added to a 500 mL three‐necked round‐bottom flask and brought to a vigorous boil under magnetic stirring. Once the temperature reached ∼100°C, 0.068–0.070 g of silver nitrate (AgNO_3_) was added and fully dissolved. Subsequently, 0.118–0.120 g of trisodium citrate dihydrate (CIT), pre‐dissolved in 12 mL of deionized water, was rapidly injected into the boiling AgNO_3_ solution. The mixture was maintained under vigorous reflux until the solution turned pale yellow, indicating AgNP formation, and heating was immediately stopped. The colloidal suspension was centrifuged at 6500 rpm for 20 min to remove large aggregates, and the resulting pellet was resuspended in deionized water. To induce mild aggregation and enhance SERS activity through hot spot generation, the AgNP suspension was mixed with an equal volume of 10 mm potassium iodide (KI) solution (v/v = 1:1) and incubated at room temperature for 6 h before use [26].

### 5.26 SERS Detection

After collecting serum or lung tissue homogenates, samples were prepared by mixing the liquid with methanol at a 6:1 ratio, followed by vortexing for 1 min and centrifugation for another minute. To prepare for Raman analysis, 14 µL of Ag@CIT was thoroughly mixed with 5 µL of the sample, which included 2 µL of deuterated methanol as an internal standard. A small volume of the resulting mixture was drawn into a capillary tube for detection. Raman measurements were conducted using a WITec Alpha 300 Raman microscope (Germany) with the following settings: 633 nm excitation wavelength, 600 grating, 20 s scan time, 10 mW laser output power, and 3 s accumulation time.

### 5.27 LOD and LOQ Determination

The limit of detection (LOD) and limit of quantification (LOQ) for MLB were calculated based on standard solution measurements using Ag@CIT SERS substrates. A series of MLB standard solutions (50–1000 ng/mL) was prepared and analyzed under identical SERS conditions as described above. The calibration curve was constructed using the intensity ratio of the MLB characteristic peak (*I*_1265_) to the internal standard peak of deuterated methanol (*I*_2078_). LOD and LOQ were calculated following the equations: *LOD* = 3.3*σ/k*, *LOQ* = 10*σ/k*, where σ represents the standard deviation of blank samples (*n* = 6), and *k* is the slope of the calibration curve.

### 5.28 Statistical Analysis

All statistical analyses were performed using GraphPad Prism 9.0. Data are presented as mean ± SD. n indicates the number of independent biological replicates or animals and is specified in each figure legend. For quantification, immunoblot signals were normalized to the corresponding loading controls, and fluorescence/intensity‐based assays were analyzed using identical acquisition settings and expressed as raw values or fold change versus the indicated control. No data transformation was applied, and no outliers were removed unless prespecified exclusion criteria were met or a technical failure occurred. Two‐group comparisons were performed using an unpaired two‐tailed Student's *t*‐test; comparisons among ≥3 groups used one‐way ANOVA with Tukey's post hoc test; and experiments with two independent variables used two‐way ANOVA with Sidak's multiple comparisons test. *P* < 0.05 was considered statistically significant.

## Author Contributions

Z.L., C.L., and B.Z. performed conceptualization. Z.L., C.L., Z.M., D.Z., R.W., Y.J.Y., G.C., C.L., Y.B., and H.C. performed methodology. Z.L. and C.L. performed formal analysis. Z.M., Y.J.Y., G.C., C.L., and Y.B. performed the investigation. C.L. and C.L. performed data curation. Z.M., R.W., Y.J.Y., and Y.B. performed the software. Z.M. performed visualization, and G.C. performed validation. D.Z., R.W., Y.J.Y., G.C., C.L., Y.B., and H.C. performed supervision. Z.L., C.L., and B.Z. acquired funding. D.Z. and B.Z. provided resources. and B.Z. performed project administration. Z.L. wrote the original draft, and B.Z. reviewed and edited the manuscript.

## Funding

National Natural Science Foundation of China grant 82502608, China Postdoctoral Science Foundation grant 2024MD763969, 2025MD784133, Postdoctoral Fellowship Program of CPSF grant GZC20250991, Second Affiliated Hospital of Harbin Medical University, Harbin Medical University Cancer Hospital.

## Conflicts of Interest

The authors declare no conflicts of interest.

## Supporting information




**Supporting File 1**: advs74152‐sup‐0001‐SuppMat.docx.

**Supporting File 2**: advs74152‐sup‐0002‐MovieS1.mp4.

**Supporting File 3**: advs74152‐sup‐0003‐MovieS2.mp4.

**Supporting File 4**: advs74152‐sup‐0004‐Data.zip.

## Data Availability

The data that support the findings of this study are available from the corresponding author upon reasonable request.
