# Supplementary material for: Targeting the GPX4–FUNDC1 Interaction with Magnesium Lithospermate B Attenuates Sepsis‐Associated Lung Injury
Source: Adv Sci (Weinh). 2026 Jan 30;13(20):e16488. doi: 10.1002/advs.202516488 (PMC13067869; doi:10.1002/advs.202516488)
Supplement: Supplementary file 1 — Supporting File 1: advs74152‐sup‐0001‐SuppMat.docx. [file ADVS-13-e16488-s001.docx]

Supporting Information

Targeting the GPX4–FUNDC1 Interaction with Magnesium Lithospermate B Attenuates Sepsis-Associated Lung Injury

Zhixi Li, Chang Liu, Zhaoxue Ma, Dongyou Zheng, Renkai Wang, Yongjing Yu, Guangmin Chen, Chenglong Li, Yue Bu, Hang Cao, Bing Zhang*

***Corresponding author: Bing Zhang

E-mail: 600771@hrbmu.edu.cn


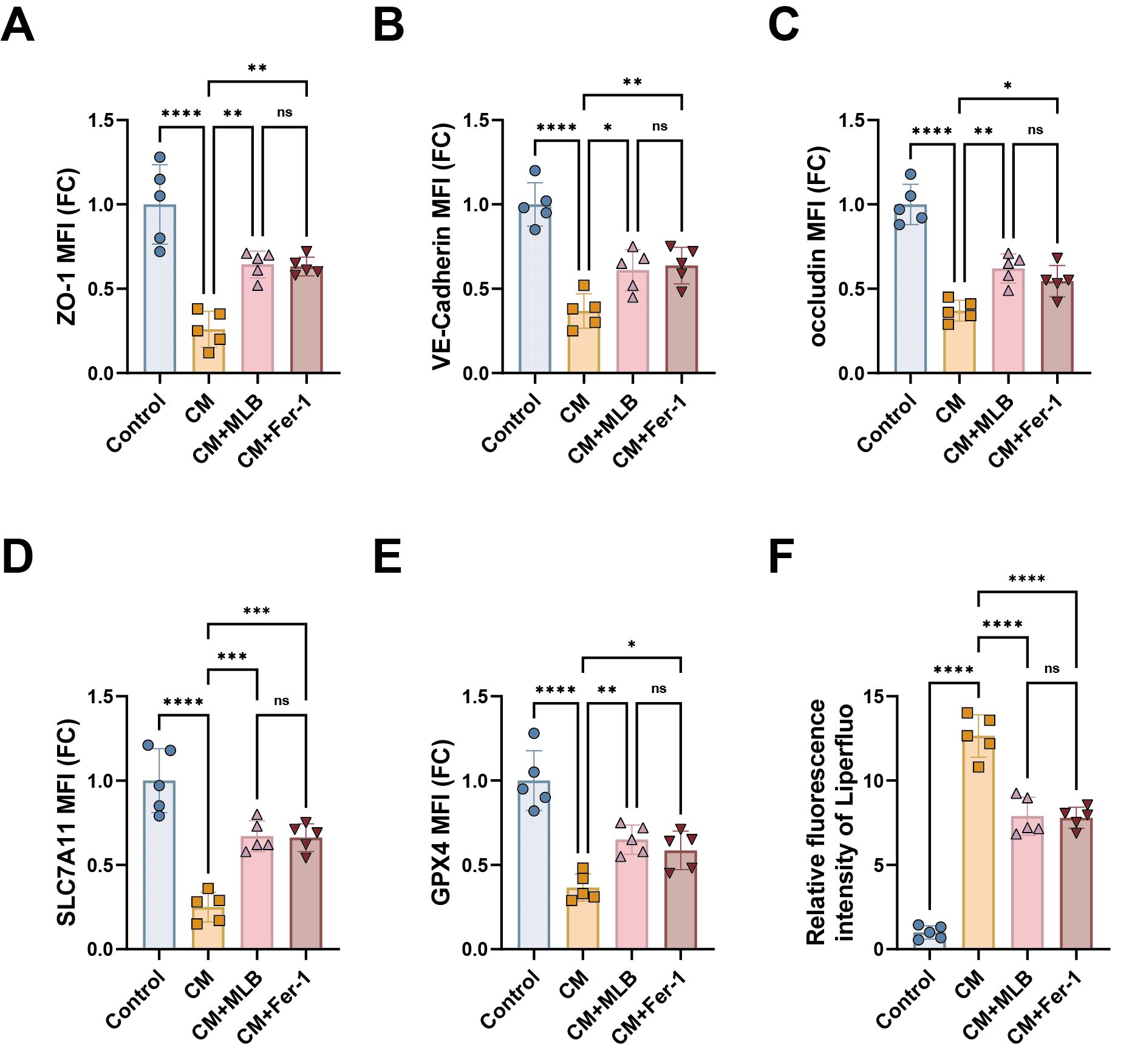


**Figure S1.** MLB alleviates ferroptosis and barrier disruption in endothelial cells exposed to LPS-Mφ conditioned medium (CM). A-C) Immunofluorescence quantification of endothelial barrier markers ZO-1, VE-Cadherin, and occludin in HPMECs treated with CM, CM + MLB (100 μM), or CM + ferrostatin-1 (1 μM) (n = 5). D,E) Quantification of ferroptosis regulators SLC7A11 and GPX4 via immunofluorescence in HPMECs (n = 5). F) Relative fluorescence intensity of Liperfluo staining in HPMECs (n = 5). Data are presented as mean ± SD. One-way ANOVA followed by Tukey’s multiple comparisons test was used to determine statistical significance. **P* < 0.05, ***P* < 0.01, ****P* < 0.001, *****P* < 0.0001, ns, not significant.


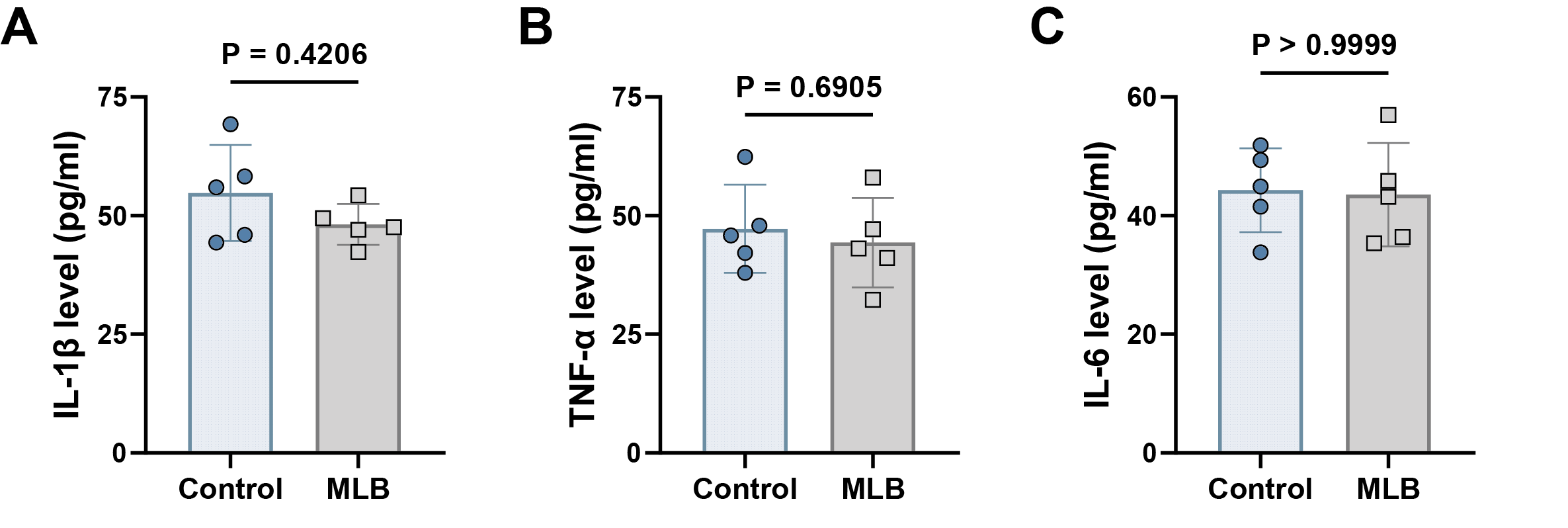


**Figure S2.** MLB treatment does not induce systemic inflammation under physiological conditions. A-C) Quantification of IL-1β, TNF-α, and IL-6 levels in lung tissues from mice treated with vehicle or MLB (n = 5). Data are presented as mean ± SD. An unpaired two-tailed Student’s *t*-test determined *P* values.


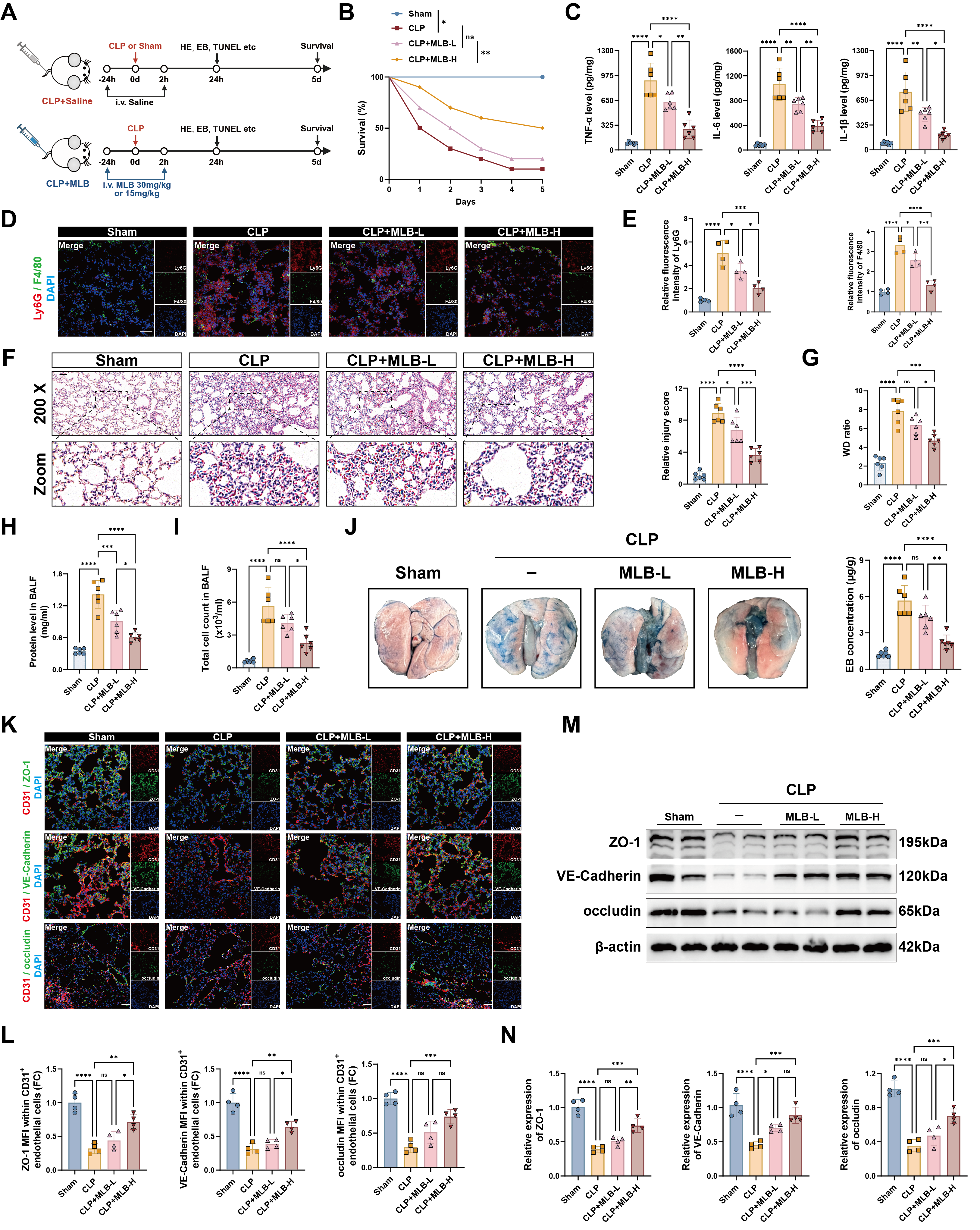


**Figure S3.** MLB preserves pulmonary vascular endothelial integrity and attenuates lung injury in CLP-induced septic mice. A) Schematic timeline of MLB administration, CLP induction, and tissue collection. B) Kaplan-Meier survival curves of CLP mice treated with low or high doses of MLB (n = 20). C) Quantification of proinflammatory cytokines (TNF-α, IL-6, and IL-1β) in lung tissue homogenates (n = 6). D,E) Representative immunofluorescence images and quantification of lung sections stained for Ly6G (neutrophils) and F4/80 (macrophages) (n = 5). Scale bar, 50 μm. F) Representative H&E-stained lung sections and quantification of lung injury scores (n = 6). Scale bar, 50 μm. G) Lung wet-to-dry (W/D) weight ratios (n = 6). H,I) Total protein concentration and total cell count in bronchoalveolar lavage fluid (BALF) (n = 6). J) Representative images and quantification of Evans blue dye extravasation in lung tissues (n = 6). K) Representative immunofluorescence staining of lung sections showing expression of endothelial junction proteins ZO-1, VE-Cadherin, and occludin. Scale bar, 50 μm. L) Quantification of ZO-1 and VE-Cadherin fluorescence intensity within CD31⁺ areas (n = 4). M,N) Representative immunoblots and quantification of ZO-1, VE-Cadherin, and occludin in lung tissues (n = 4). Data are presented as mean ± SD. One-way ANOVA followed by Tukey’s multiple comparisons test was used to determine statistical significance. **P* < 0.05, ***P* < 0.01, ****P* < 0.001, *****P* < 0.0001, ns, not significant.


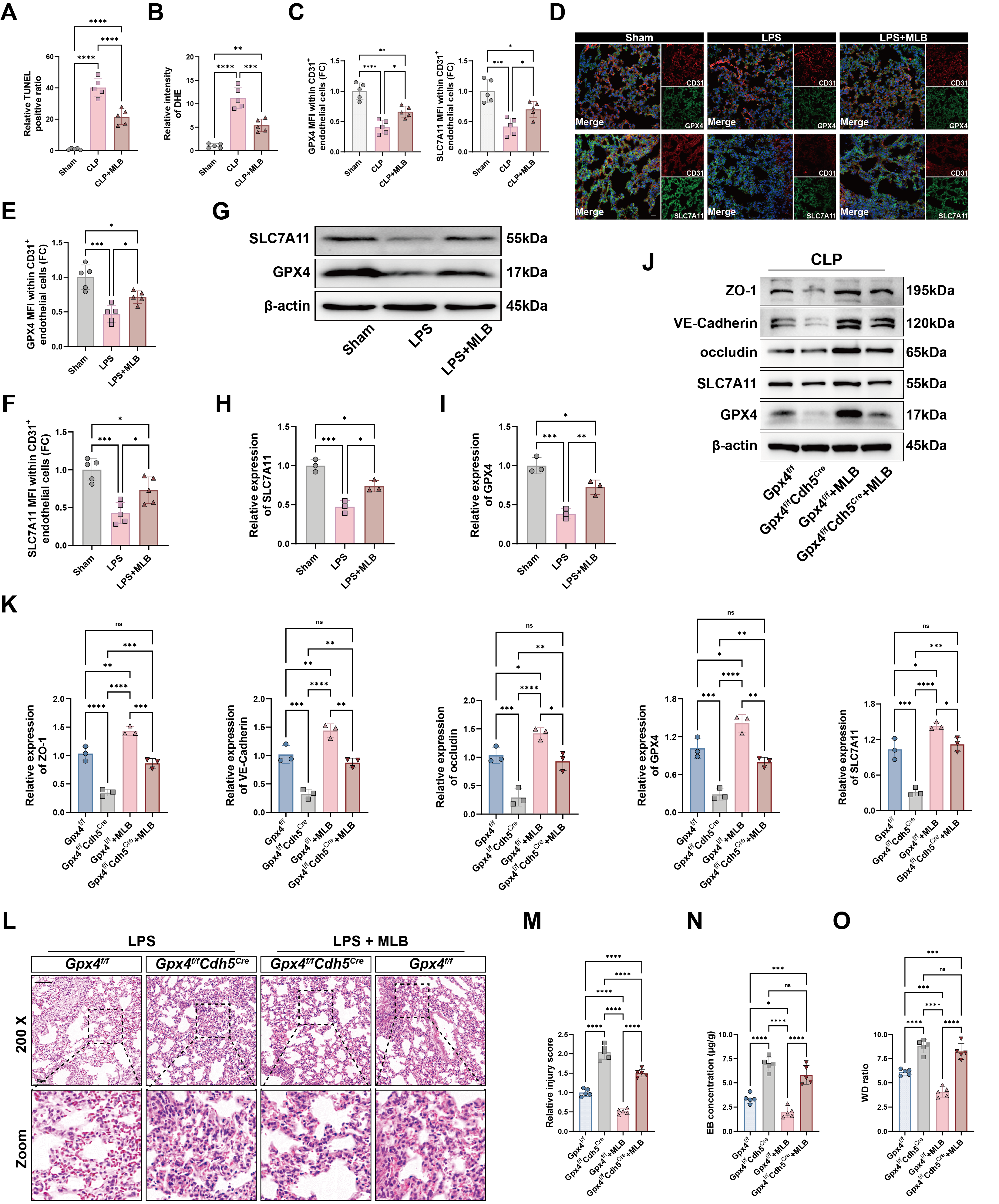


**Figure S4.** MLB attenuates pulmonary ferroptosis and vascular injury through GPX4-dependent mechanisms. A,B) Quantification of TUNEL-positive and DHE intensity in lung tissues from Sham, CLP, or CLP + MLB (30 mg/kg) groups (n = 5). C) Quantification of GPX4 and SLC7A11 mean fluorescence intensity (MFI) (fold change vs Sham) within CD31^+^ endothelial cells (n = 5). D) Representative immunofluorescence images of CD31-labeled pulmonary vessels (red) co-stained with GPX4 or SLC7A11 (green). Scale bar, 50 μm. E,F) Quantification of GPX4 and SLC7A11 mean fluorescence intensity (MFI) within CD31^+^ endothelial cells (n = 5). G) Immunoblotting of GPX4 and SLC7A11 expression in lung tissues. H,I) Quantification of GPX4 and SLC7A11 protein expression by immunoblotting (n = 3). J,K) Immunoblotting analysis of endothelial junction proteins (ZO-1, VE-Cadherin, occludin) and ferroptosis-related proteins (SLC7A11, GPX4) in lungs from MLB-treated *Gpx4^f/f^* and *Gpx4^f/f^Cdh5^Cre^* mice 24 h after CLP (n = 3). L,M) Representative H&E-stained lung sections and corresponding lung injury scores in LPS-treated mice (n = 5). Scale bar, 50 μm. N,O) Quantification of vascular permeability (Evans blue leakage) and lung wet/dry weight ratios in the same LPS-induced lung injury model (n = 5). Data are presented as mean ± SD. One-way ANOVA followed by Tukey’s multiple comparisons test was used to determine statistical significance. **P* < 0.05, ***P* < 0.01, ****P* < 0.001, *****P* < 0.0001, ns, not significant.


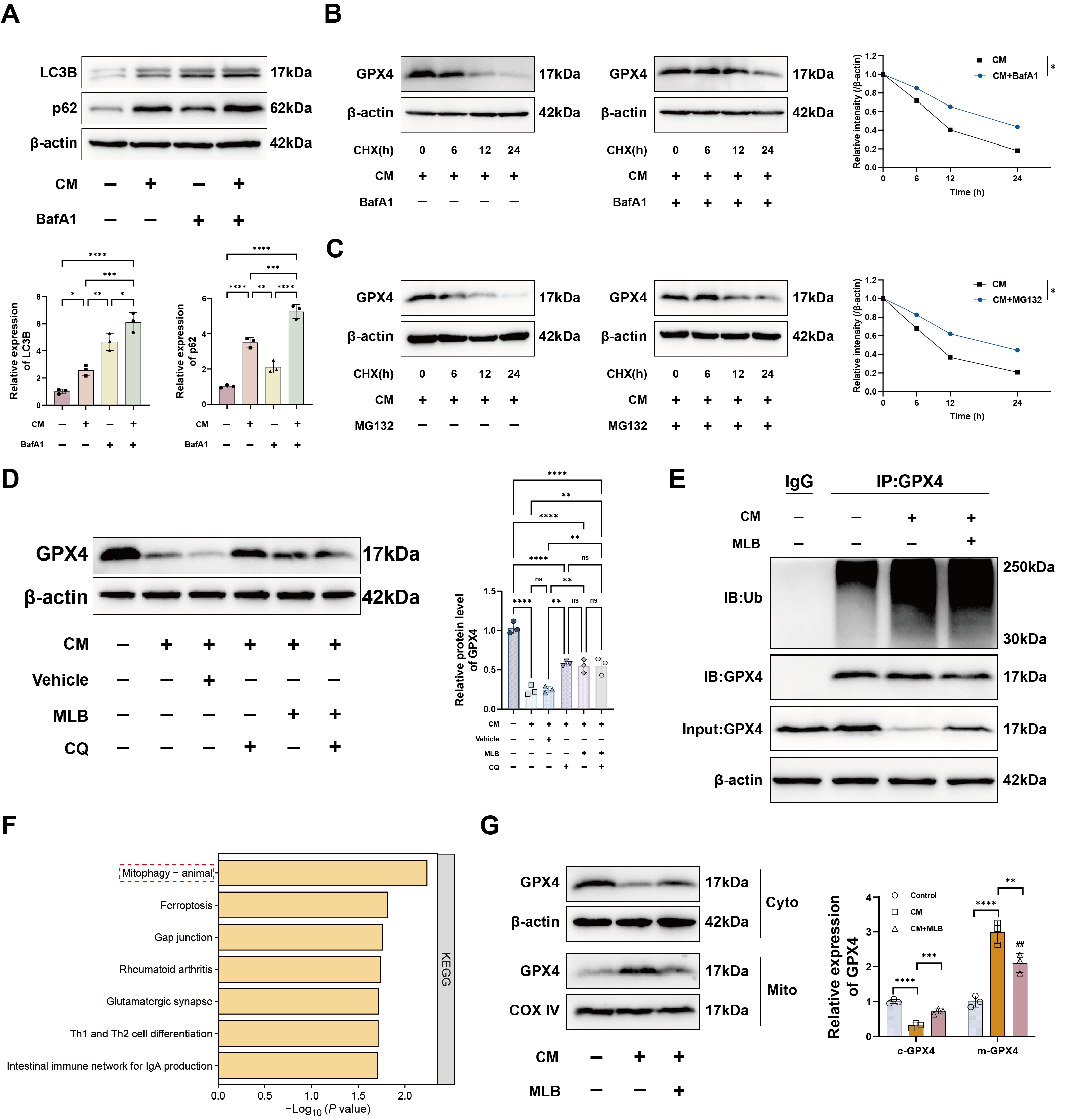


**Figure S5.** MLB stabilizes GPX4 by limiting lysosome-associated degradation and preserves mitochondrial homeostasis under CM conditions. A) Immunoblot analysis of LC3B and p62 in HPMECs treated with CM and/or BafA1 (100 nM) for 12 h (n = 3). B,C) Cycloheximide (CHX) chase assays were conducted in HPMECs exposed to CM for the indicated time points (0-24 h), with or without BafA1 (B) or MG132 (C) (n = 3). D) Immunoblot analysis of GPX4 in HPMECs under CM conditions treated with vehicle, MLB, CQ (50 μM), or MLB + CQ (n = 3). E) Ubiquitination status of GPX4 assessed by immunoblotting. F) KEGG pathway enrichment analysis of differentially expressed genes in pulmonary endothelial cells from CLP mouse scRNA-seq data. G) Subcellular fractionation and immunoblotting analysis of GPX4 in cytosolic and mitochondrial compartments from CM- and/or MLB-treated HPMECs (n = 3). Data are presented as mean ± SD. One-way ANOVA followed by Tukey’s multiple comparisons test for (A,D,G) and two-way ANOVA with Sidak’s multiple comparisons test for (B,C), were used for statistical analysis. **P* < 0.05, ***P* < 0.01, ****P* < 0.001, *****P* < 0.0001, ns, not significant.


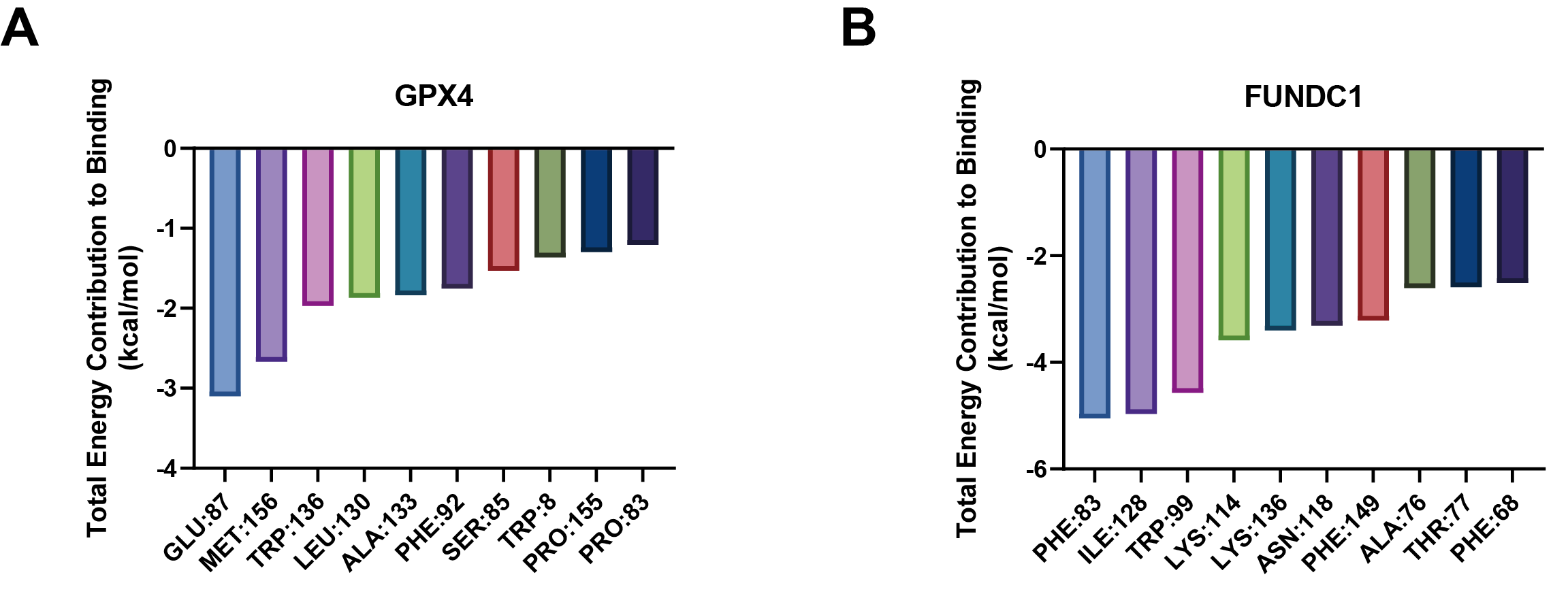


**Figure S6.** Per-residue energy decomposition confirms a stable GPX4-FUNDC1 interaction. A,B) Molecular dynamics simulations combined with per-residue energy decomposition identified key residues contributing to the stability of the GPX4-FUNDC1 complex. The top 10 binding residues from GPX4 and FUNDC1, ranked by binding free energy contribution, are presented.


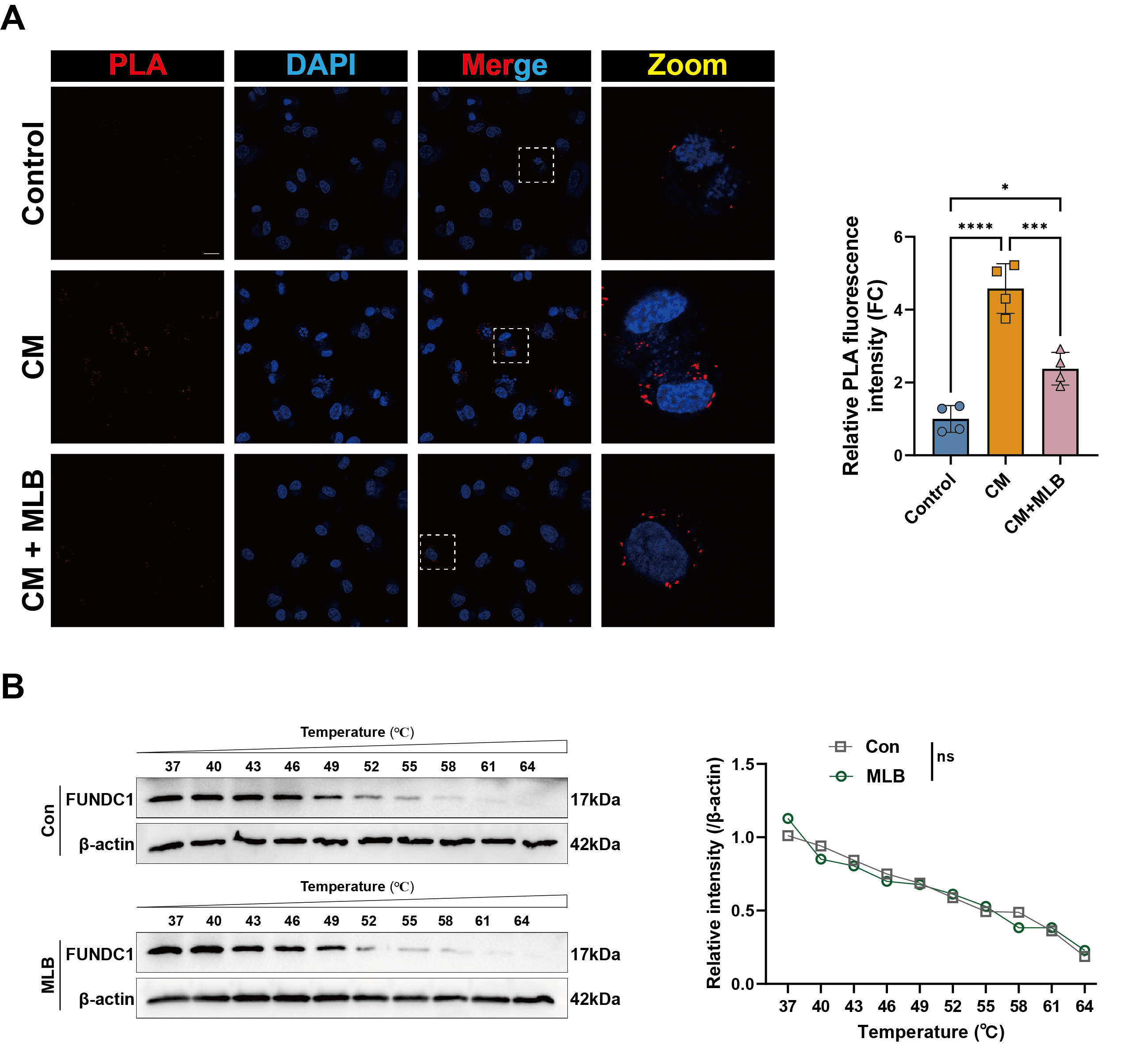


**Figure S7.** MLB disrupts CM‑induced GPX4-FUNDC1 interaction without directly targeting FUNDC1. A) Representative proximity ligation assay (PLA) images showing the interaction between endogenous GPX4 and FUNDC1 in HPMECs (n = 4). B) Cellular thermal shift assay (CETSA) evaluating the thermal stability of FUNDC1 in HPMECs treated with vehicle (Con) or MLB (n = 3). Data are presented as mean ± SD. Statistical significance was determined by one‑way ANOVA with Tukey’s post hoc test for PLA quantification (A) and two‑way ANOVA with Sidak’s multiple comparisons test for CETSA analysis (B).


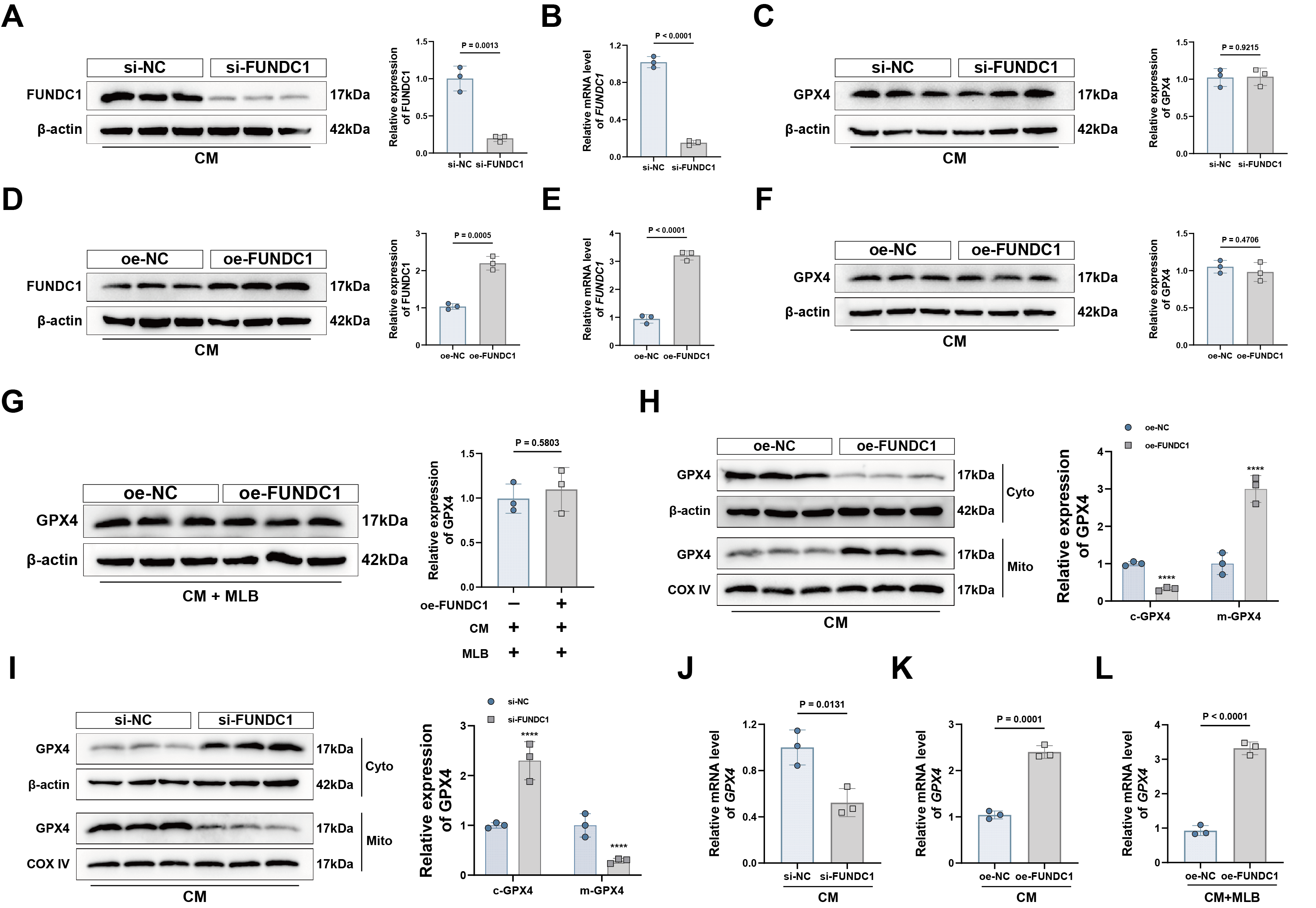


**Figure S8.** FUNDC1 regulates GPX4 localization and transcription without affecting total protein levels. A,D) Representative immunoblots showing *FUNDC1* knockdown or overexpression efficiency in HPMECs following siRNA transfection or plasmid transduction, respectively (n = 3). B,E) RT-qPCR analysis confirming the silencing and overexpression efficiency of *FUNDC1* in HPMECs (n = 3). C,F) Western blot analysis of GPX4 protein levels upon *FUNDC1* knockdown or overexpression under CM conditions (n = 3). G) Western blot analysis and quantification of GPX4 protein levels after MLB treatment in *FUNDC1*-overexpressing HPMECs (n = 3). H,I) Mitochondrial and cytosolic fractionation analysis showing compartmental redistribution of GPX4 under CM conditions (n = 3). J-L) RT-qPCR analysis of *GPX4* mRNA levels (n = 3). Data are presented as mean ± SD. An unpaired two-tailed Student’s *t*-test determined *P* values.

**
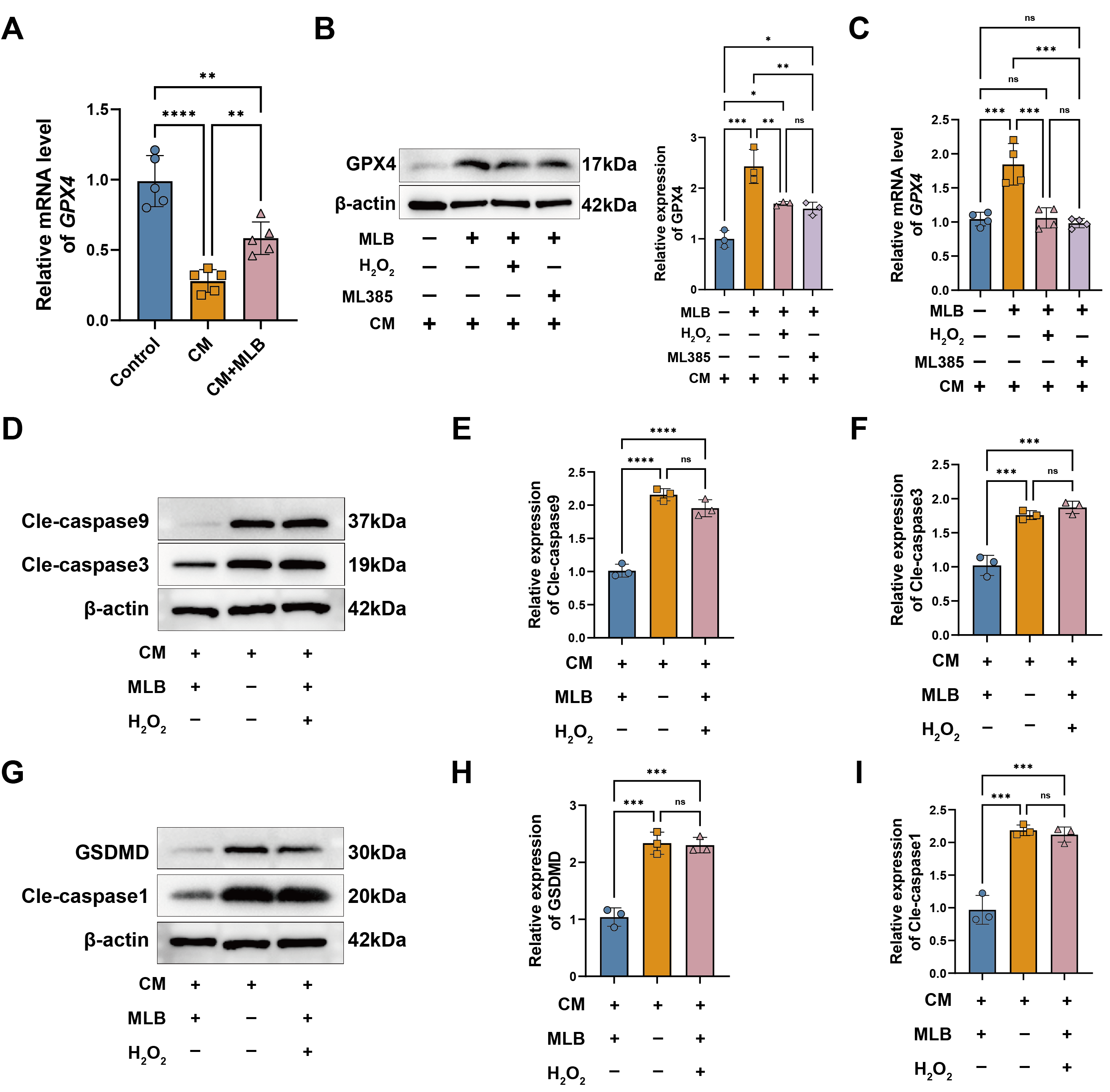
**

**Figure S9.** Elevated ROS levels attenuate MLB-mediated GPX4 upregulation and reverse its inhibitory effects on apoptosis and pyroptosis. A) RT-qPCR quantification of *GPX4* mRNA levels (n = 5). B) Western blot analysis and quantification showing GPX4 protein expression in HPMECs (n = 3). C) RT-qPCR quantification of *GPX4* mRNA levels (n = 4). D-F) Immunoblotting and quantification of cleaved caspase-9 (Cle-caspase9) and cleaved caspase-3 (Cle-caspase3) in HPMECs with or without MLB and/or H₂O₂ treatment (n = 3). G-I) Immunoblotting and quantification of GSDMD and cleaved caspase-1 (Cle-caspase1) in HPMECs (n = 3). Data are presented as mean ± SD. Statistical significance was determined by one-way ANOVA followed by Tukey’s post hoc test. **P* < 0.05, ***P* < 0.01, ****P* < 0.001, *****P* < 0.0001, ns, not significant.


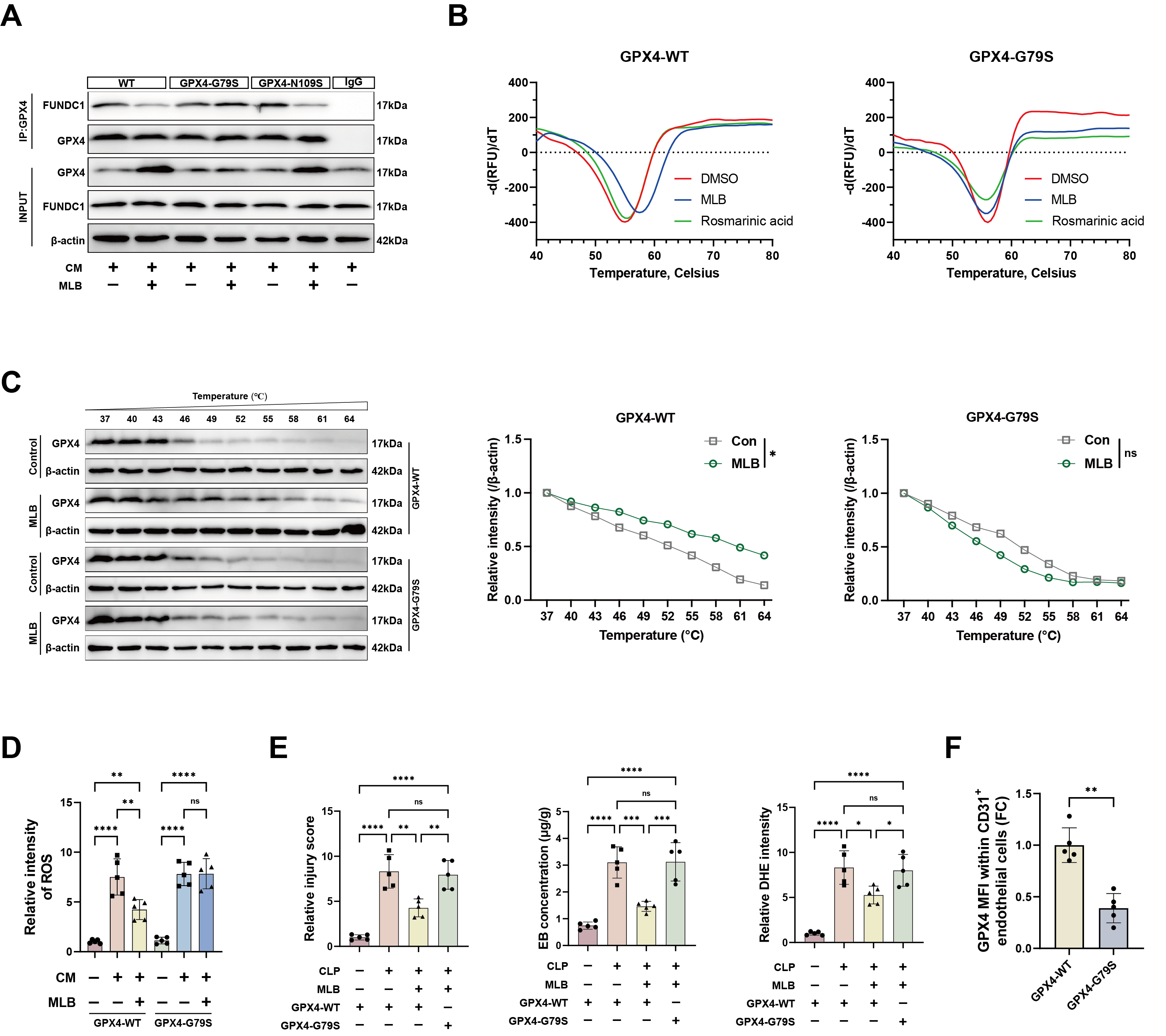


**Figure S10.** Gly79 of GPX4 is essential for MLB-GPX4 interaction and functional protection in endothelial cells and septic mice. A) Co-immunoprecipitation showing MLB-induced disruption of GPX4-FUNDC1 interaction in HPMECs expressing GPX4-WT or GPX4 mutants (G79S, N109S). B) Differential scanning fluorimetry (DSF) analysis of recombinant GPX4-WT and GPX4-G79S proteins in the presence of MLB, Rosmarinic acid, or vehicle (DMSO). C) Cellular thermal shift assay (CETSA) detection of MLB’s stabilizing effect on GPX4-WT and GPX4-G79S (n = 3). D) Quantification of ROS levels in HPMECs expressing GPX4-WT or GPX4-G79S under CM ± MLB conditions (n = 5). E) Lung injury scores, Evans blue (EB) leakage, and DHE staining intensity in CLP mice injected with AAV-GPX4-WT or AAV-GPX4-G79S, with or without MLB treatment (n = 5). F) Quantification of GPX4 mean fluorescence intensity (MFI) within CD31⁺ endothelial cells by flow cytometry (n = 5). Data are presented as mean ± SD. One-way ANOVA followed by Tukey’s multiple comparisons test for (D,E), two-way ANOVA with Sidak’s multiple comparisons test for (C), or unpaired two-tailed Student’s *t*-test for (F). **P* < 0.05, ***P* < 0.01, ****P* < 0.001, *****P* < 0.0001, ns, not significant.


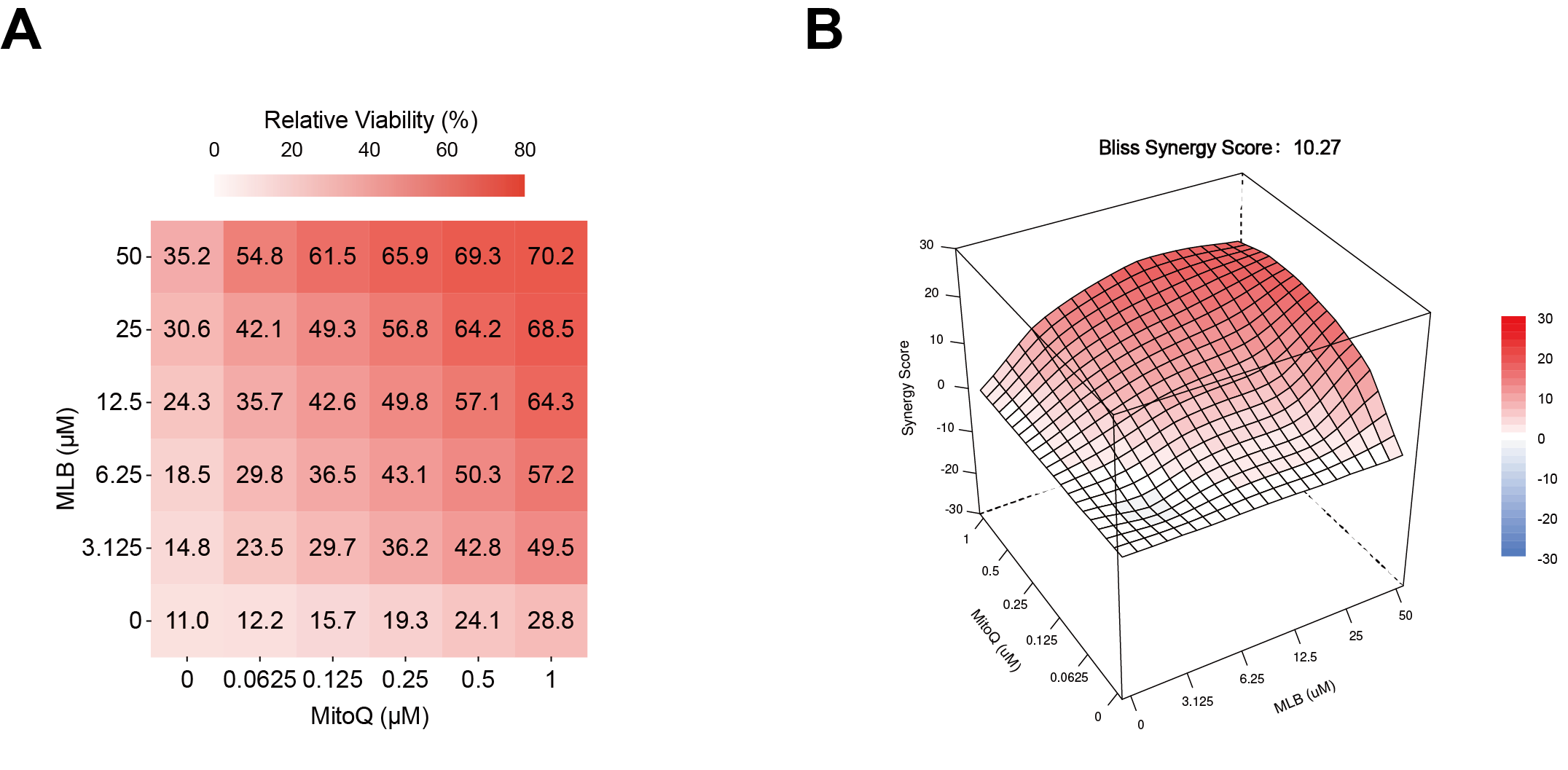


**Figure S11.** MLB-MitoQ co-treatment synergistically improves HPMEC viability under CM-induced stress. A) Heatmap of relative viability in CM-treated HPMECs following co-treatment with varying concentrations of MLB (0-50 μM) and MitoQ (0-1 μM), as assessed by the CCK-8 assay (n = 3 per dose combination). B) 3D surface plot depicting Bliss synergy scores across the dose-response matrix. Synergy scores were computed using the Bliss independence model.


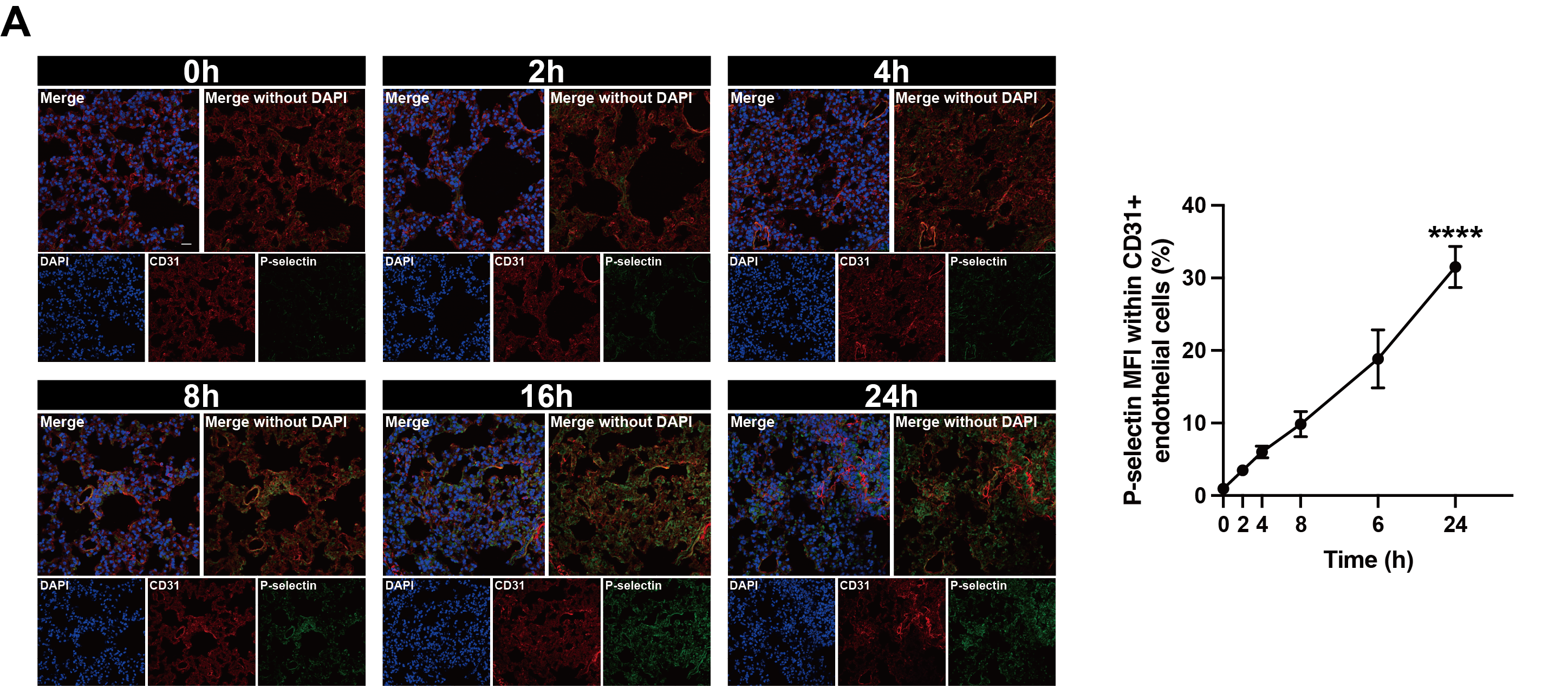


**Figure S12.** Time-dependent upregulation of P-selectin expression in pulmonary vascular endothelium after CLP. A) Representative immunofluorescence images and quantitative analysis of P-selectin signal intensity in CD31⁺ endothelial cells at 0-24 h post-CLP (n = 4). Data are presented as mean ± SD. One-way ANOVA followed by Tukey’s post hoc test determined *P* values. *****P* < 0.0001. Scale bar, 20 μm.


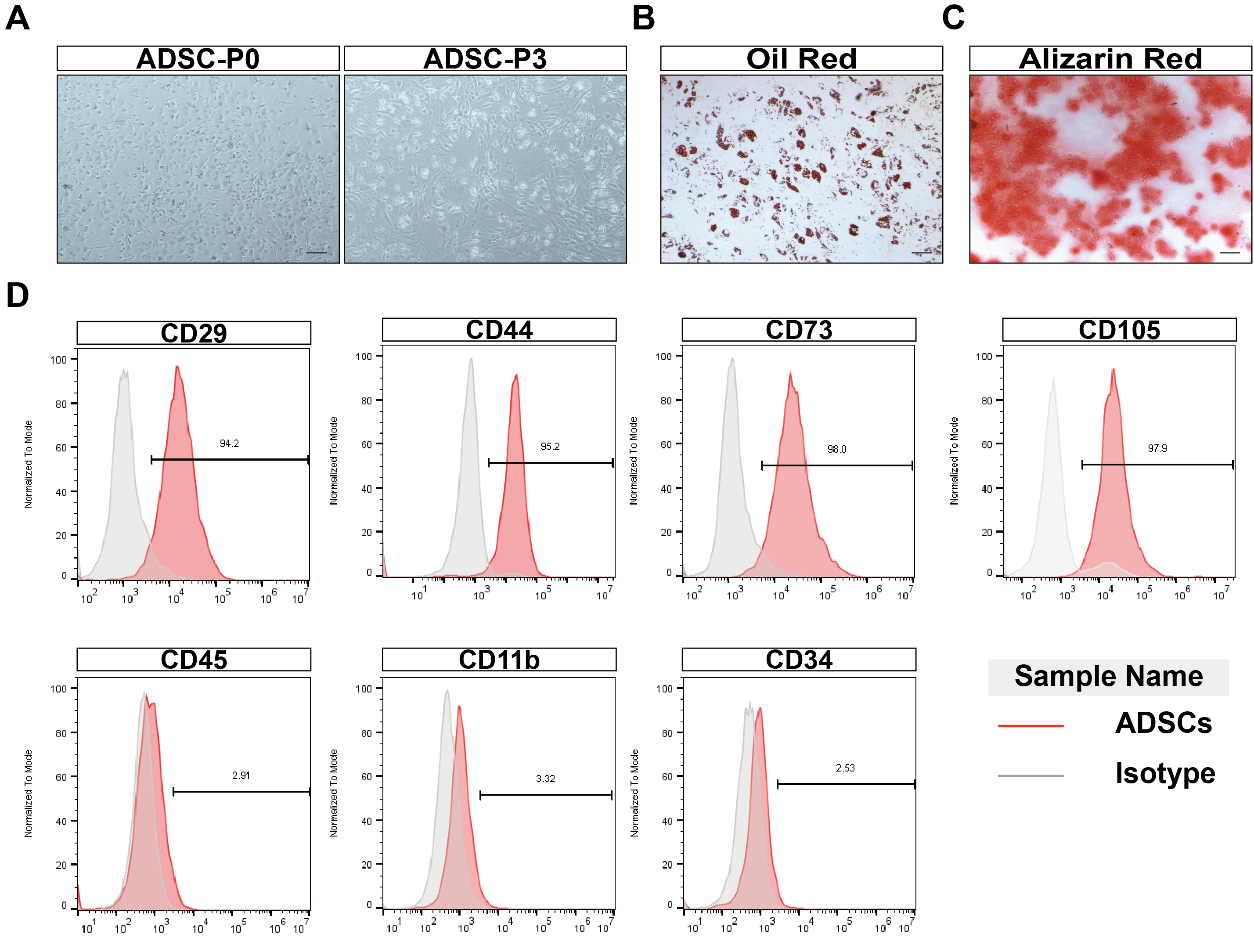


**Figure S13.** Characterization of murine adipose-derived stem cells (ADSCs). A) Representative bright-field images showing the morphology of primary (P0) and third-passage (P3) ADSCs. Scale bar, 50 μm. B) Oil Red staining of ADSCs after 7 days of adipogenic induction, revealing intracellular lipid droplet accumulation. Scale bar, 50 μm. C) Alizarin Red staining of ADSCs after 14 days of osteogenic induction, showing mineralized calcium nodules. Scale bar, 50 μm. D) Flow cytometric analysis of surface marker expression on ADSCs. Cells were positive for CD29, CD44, CD73, and CD105, and negative for hematopoietic and myeloid markers CD45, CD11b, and CD34, consistent with MSC identity.


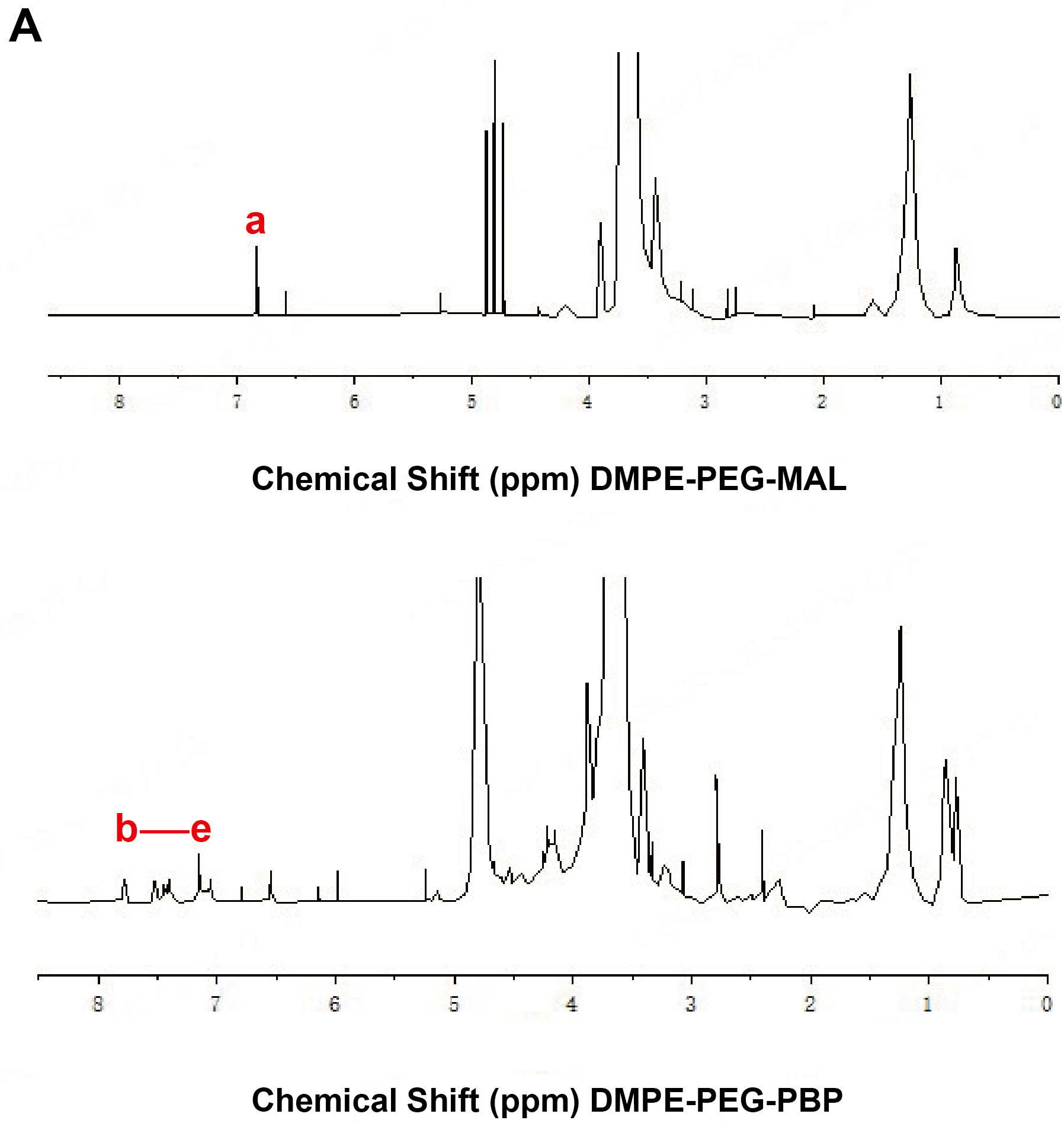


**Figure S14.** Structural validation of the synthesized DMPE-PEG-PBP (DPP) conjugate. A) Representative ^1^H NMR spectra in D₂O confirming the successful conjugation. The top panel shows the spectrum of DMPE-PEG-MAL, with the characteristic maleimide proton peak (a) at ~6.8 ppm. The bottom panel displays the spectrum of DMPE-PEG-PBP (DPP), in which the disappearance of the maleimide signal and the appearance of new peaks (b-e) corresponding to PBP protons indicate successful conjugation.


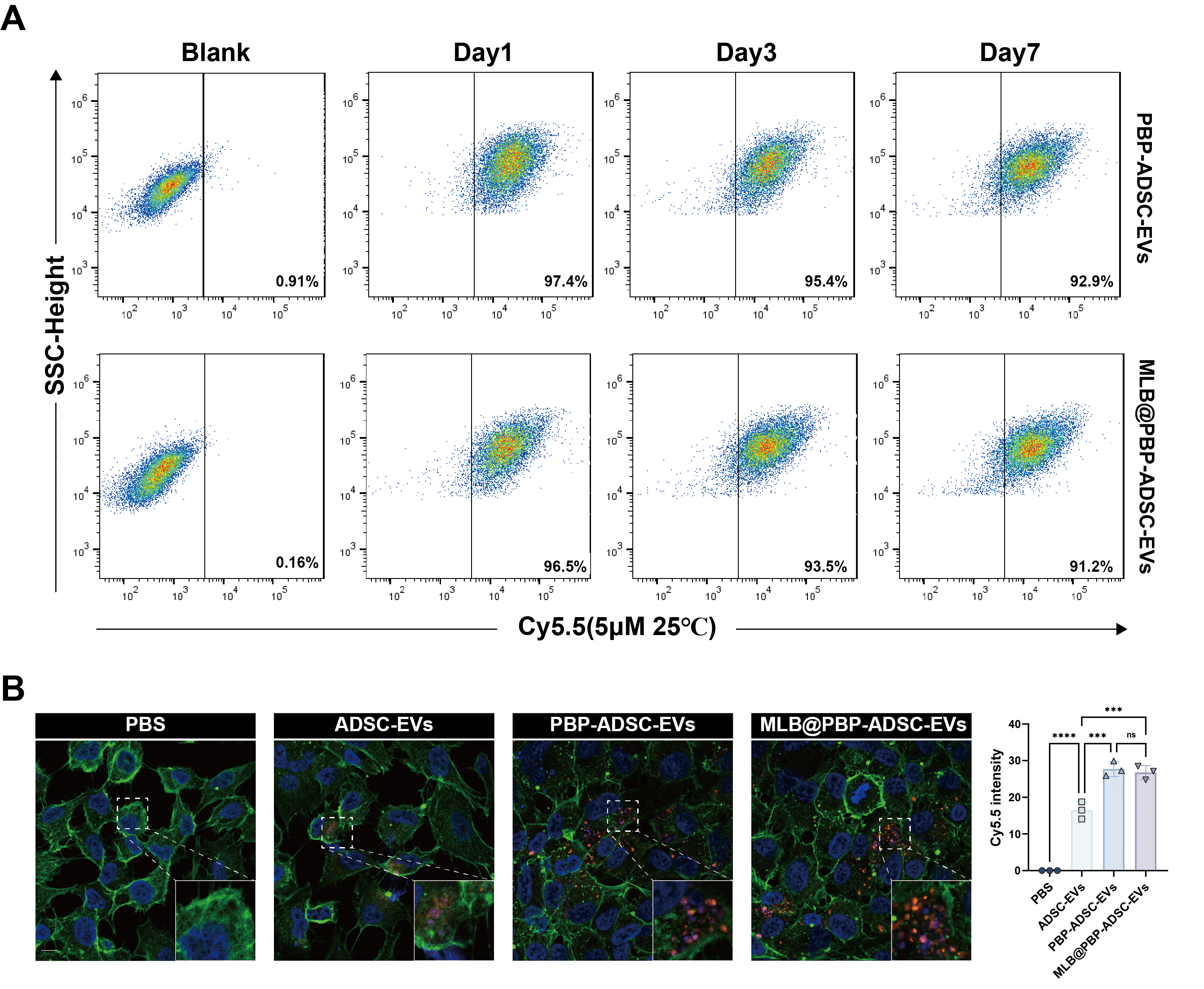


**Figure S15.** MLB@PBP-ADSC-EVs exhibit stability over 7 days and efficiently target injured HPMECs. A) Flow cytometry to evaluate the stability of PBP-ADSC-EVs and MLB@PBP-ADSC-EVs preserved at 4 °C for 1, 3, and 7 days. B) Internalization of Cy5.5-labeled EVs (orange) by phalloidin-stained HPMECs (green) upon CM treatment (n = 3). Scale bar, 10 μm. Data are presented as mean ± SD. Statistical significance was determined by one-way ANOVA followed by Tukey’s post hoc test. **P* < 0.05, ***P* < 0.01, ****P* < 0.001, *****P* < 0.0001, ns, not significant.


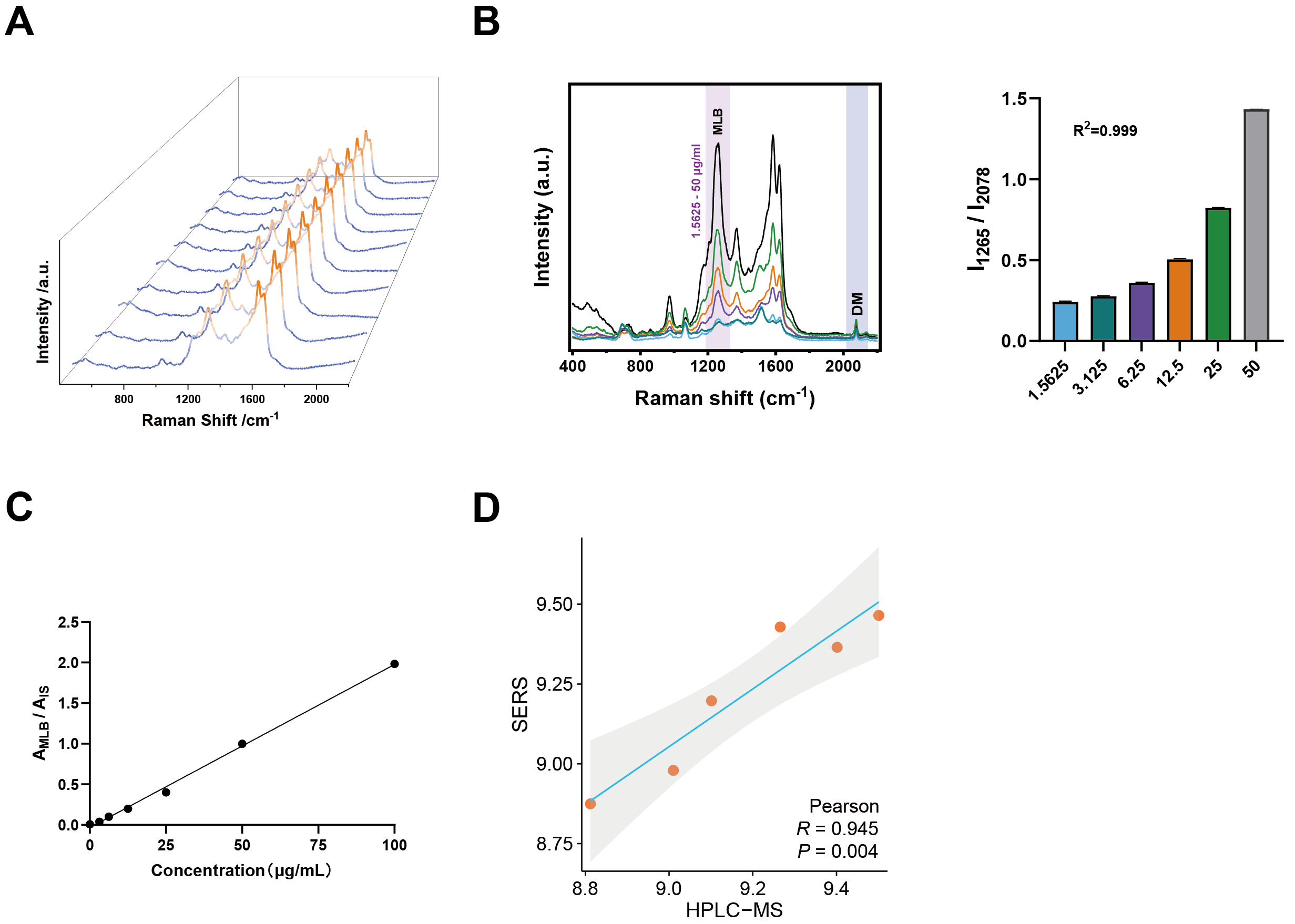


**Figure S16.** SERS-based quantitative strategy for MLB detection and orthogonal validation. A) Three-dimensional stacked SERS spectra of MLB acquired using Ag@CIT substrates across increasing concentrations. B) Representative SERS spectra of MLB at concentrations ranging from 1.5625 to 50 μg/mL, with characteristic MLB peak (I₁₂₆₅) and deuterated methanol (DM) internal standard peak (I₂₀₇₈) highlighted. Right panel, calibration plot of the intensity ratio I₁₂₆₅/I₂₀₇₈ versus MLB concentration. C) A high-performance liquid chromatography–mass spectrometry (HPLC–MS) calibration curve for MLB quantification, plotting peak area normalized to internal standard against MLB concentration. D) Correlation analysis between SERS- and HPLC-MS-determined MLB concentrations in mouse serum samples (n = 6).


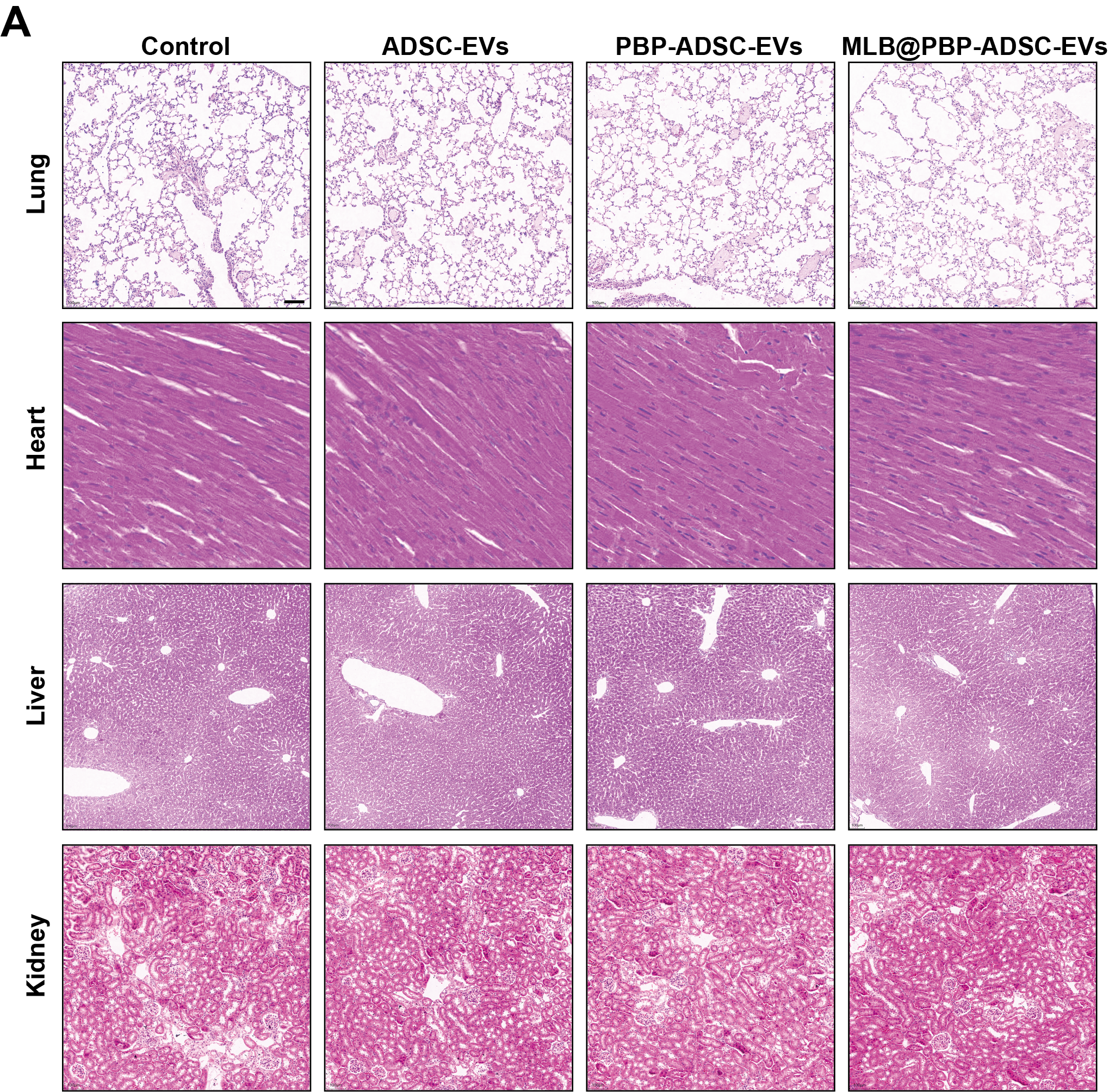


**Figure S17.** MLB@PBP-ADSC-EVs exhibit no histological toxicity in major organs. A) Representative H&E staining images of lung, heart, liver, and kidney tissues from healthy mice treated with ADSC-EVs, PBP-ADSC-EVs, or MLB@PBP-ADSC-EVs. Scale bar, 100 μm.


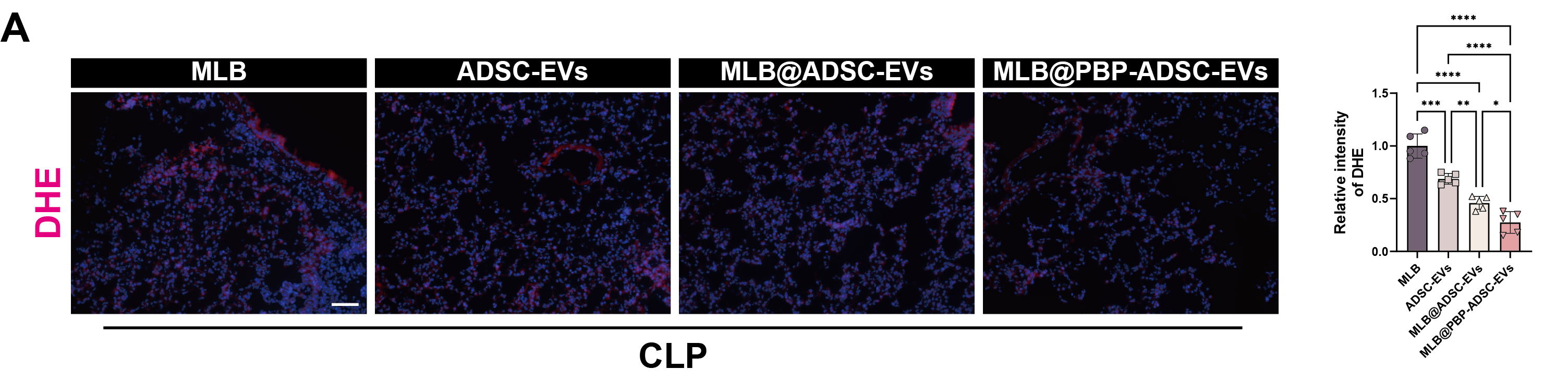


**Figure S18.** MLB@PBP-ADSC-EVs attenuate oxidative stress in the lungs of CLP-induced septic mice. A) Representative images and quantification of DHE staining in lung sections (n = 5). Scale bar, 100 μm. Statistical significance was determined by one-way ANOVA followed by Tukey’s post hoc test. **P* < 0.05, ***P* < 0.01, ****P* < 0.001, *****P* < 0.0001, ns, not significant.

**Table S1.** Statistical data of effect sizes with 95% CIs for barrier endpoints in Figure 1.

| **Comparison** | **Mean diff.^1^**  **(95% CI)** | **Cohen's *d***  **(95% CI)** | ***P* value** |
| --- | --- | --- | --- |
| **EB concentration (μg/g)** |  |  |  |
| *Gpx4^f/f^*+Sham vs *Gpx4^f/f^*+CLP | 2.13  (0.287 to 3.973) | 2.6764  (0.969 to 4.38) | 0.0208 |
| *Gpx4^f/f^Cdh5^Cre^*+Sham vs *Gpx4^f/f^Cdh5^Cre^*+CLP | 4.366  (2.523 to 6.209) | 4.31  (2.051 to 6.569) | <0.0001 |
| *Gpx4^f/f^*+CLP vs *Gpx4^f/f^Cdh5^Cre^*+CLP | 2.38  (0.537 to 4.223) | 2.061  (0.527 to 3.594) | 0.0095 |
| **WD ratio** |  |  |  |
| *Gpx4^f/f^*+Sham vs *Gpx4^f/f^*+CLP | 2.672  (1.212 to 4.132) | 5.419  (2.740 to 8.097) | 0.0004 |
| *Gpx4^f/f^Cdh5^Cre^*+Sham vs *Gpx4^f/f^Cdh5^Cre^*+CLP | 5.318  (3.858 to 6.778) | 5.955  (3.066 to 8.844) | <0.0001 |
| *Gpx4^f/f^*+CLP vs *Gpx4^f/f^Cdh5^Cre^*+CLP | 3.046  (1.586 to 4.506) | 3.741  (1.686 to 5.796) | 0.0001 |

¹ Effect size was calculated as the mean of the latter group minus the mean of the former group.

**Table S2.** The 19 compounds targeting GPX4.

| **Name** | **MMGBSA** **dG Bi** **(kcal/mol)** | **2D structure** |
| --- | --- | --- |
| Myricoside | -47.0 | 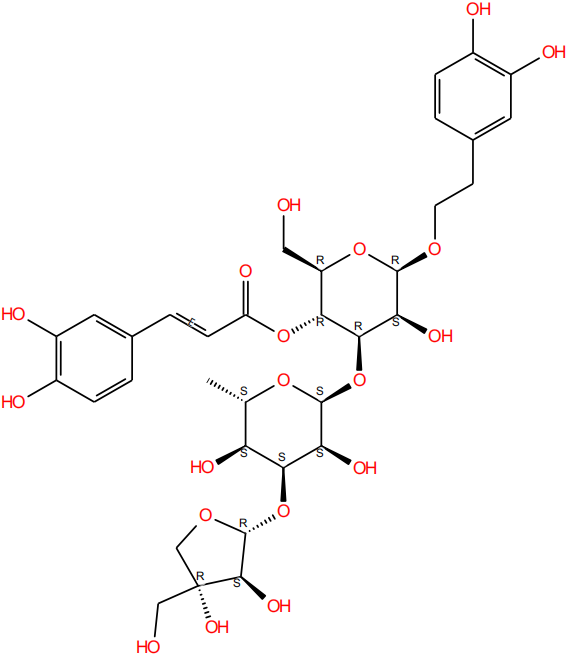 |
| Apigenin-7-O-(2G-rhamnosyl)gentiobioside | -44.43 | 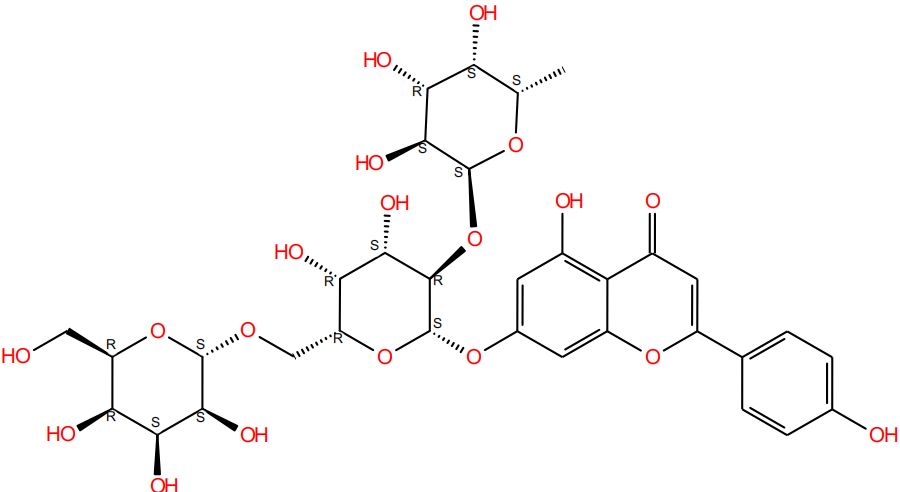 |
| D-(+)-Melezitose hydrate | -42.8 | 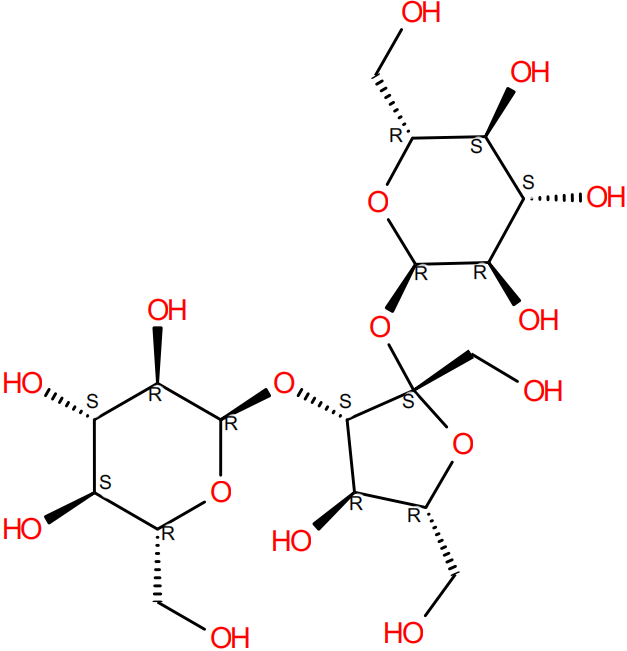 |
| Magnesium Lithospermate B | -39.72 | 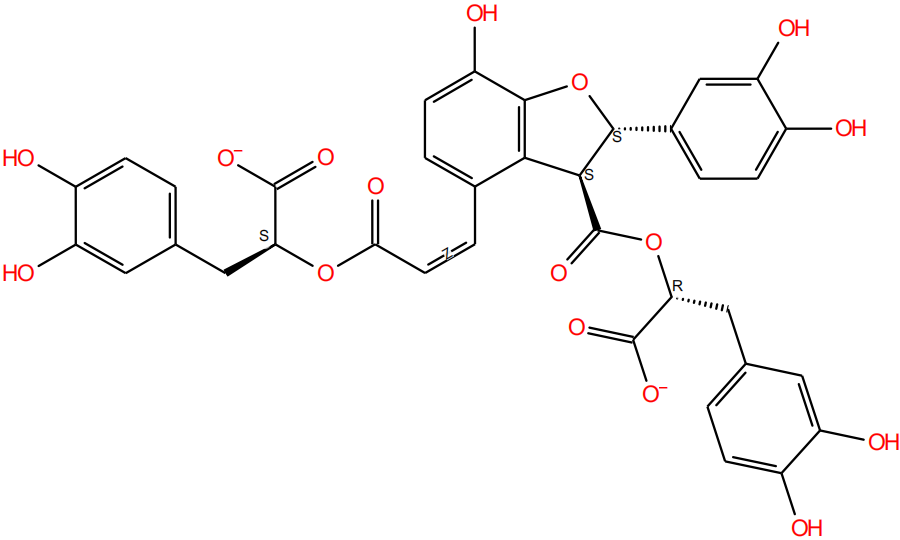 |
| Ginsenoside Rg2 | -36.99 | 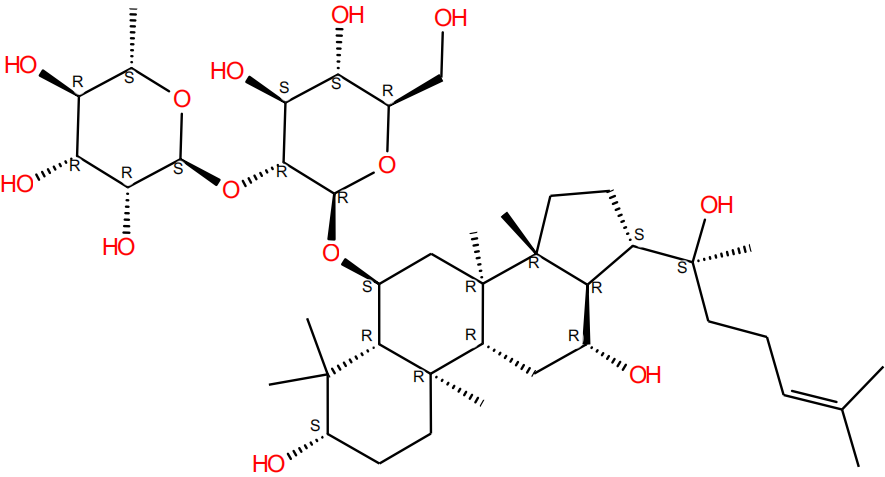 |
| 4,5-Dicaffeoylquinic acid | -36.5 | 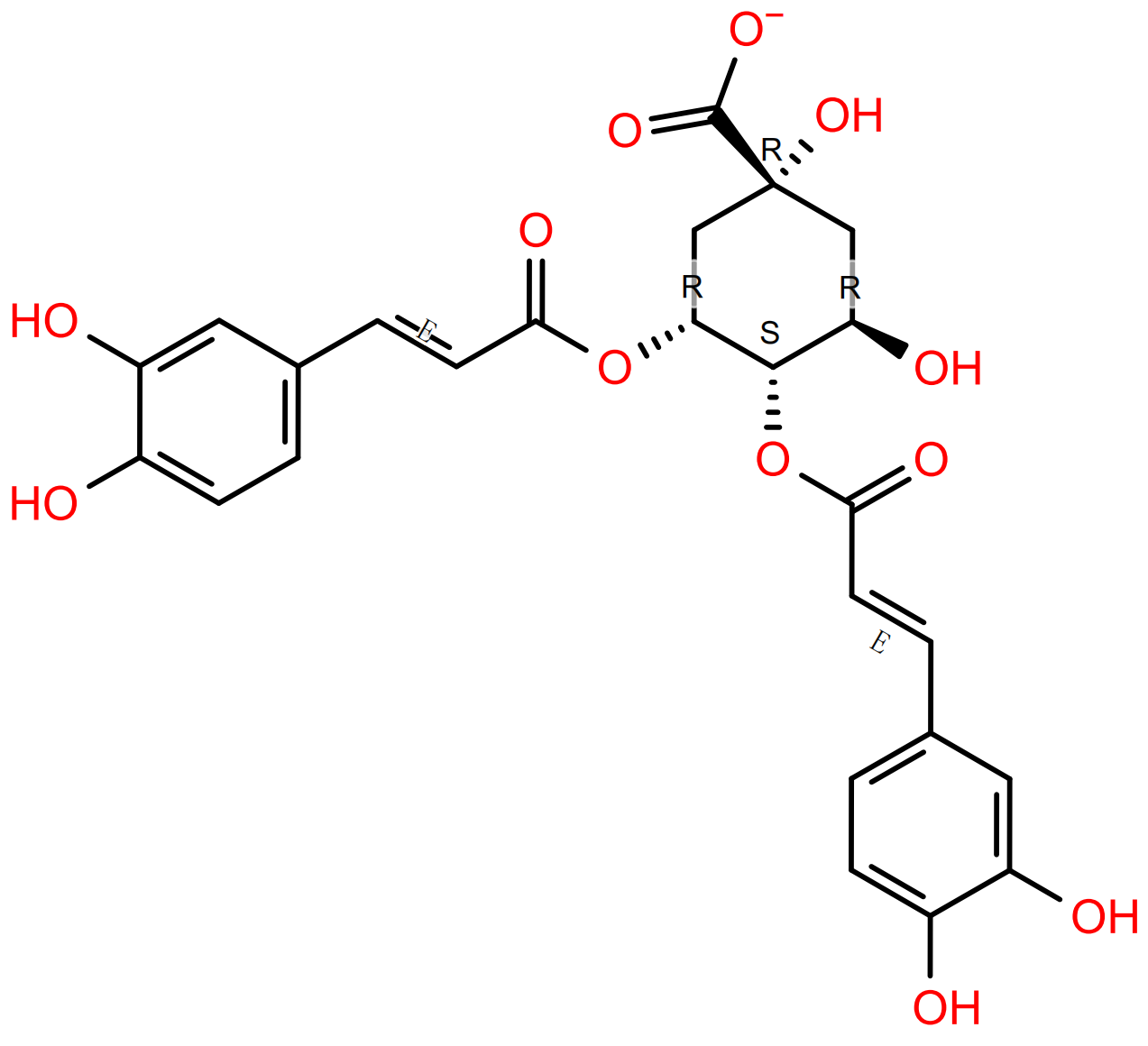 |
| Rutin hydrate | -36.45 | 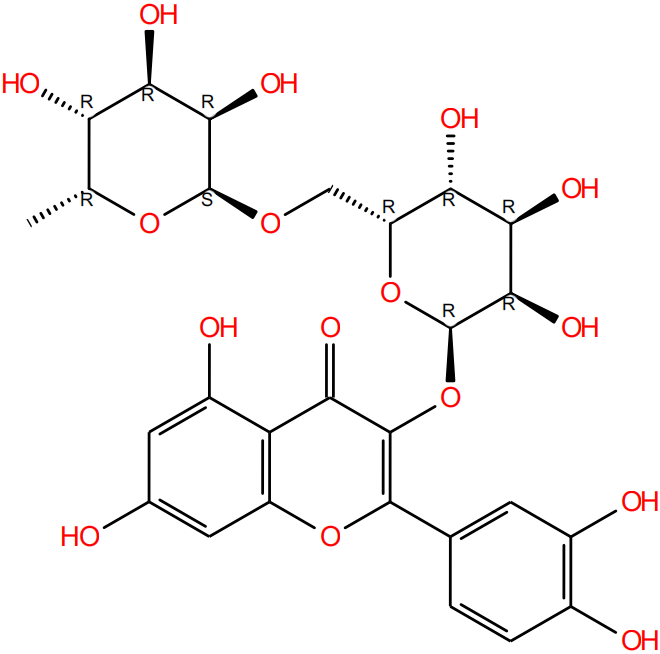 |
| Parishin B | -36.44 | 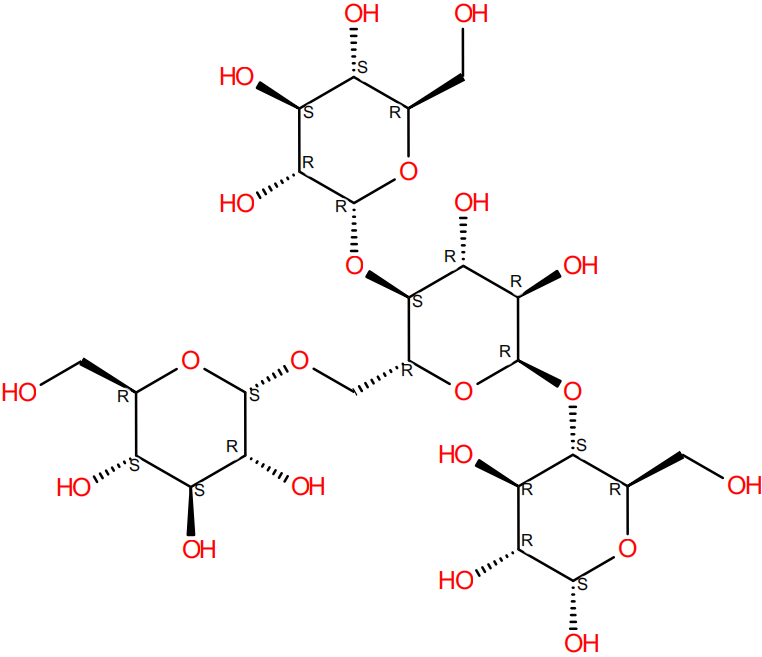 |
| Baimaside | -36.4 | 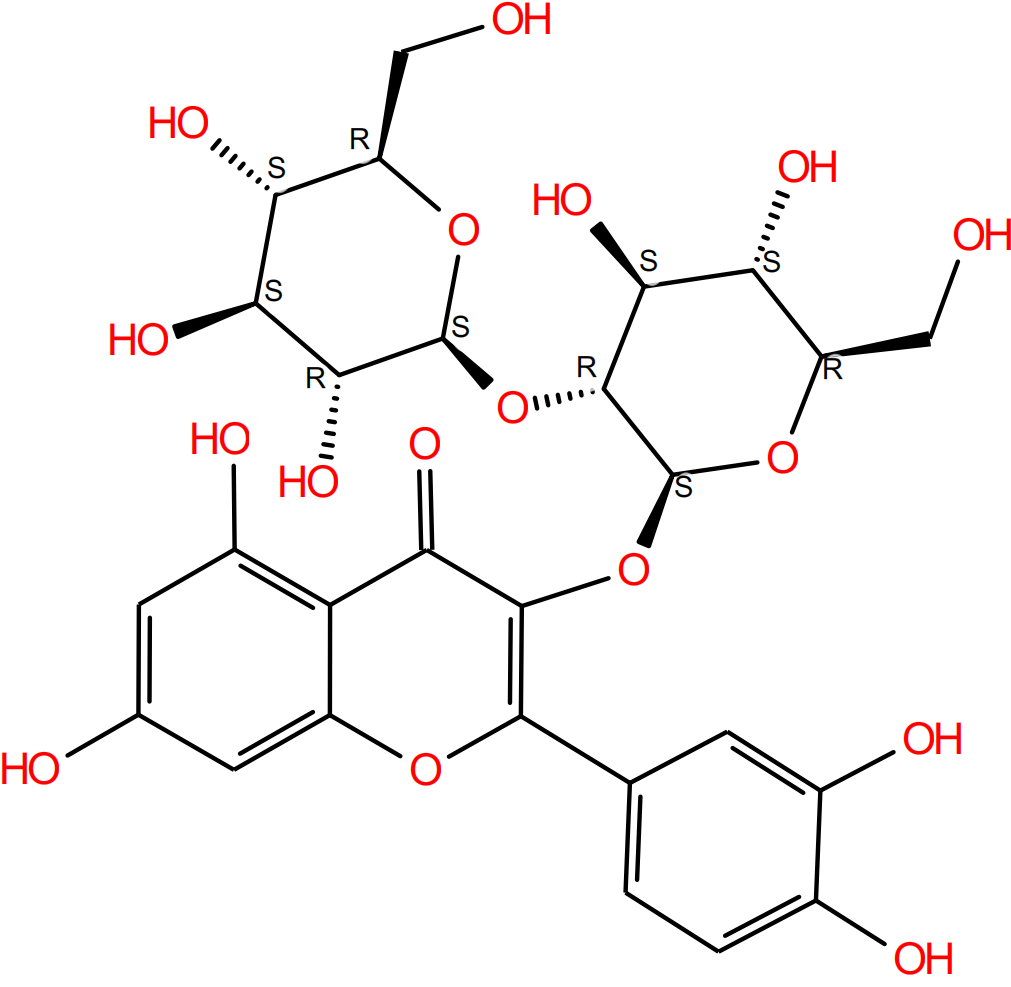 |
| Stachyose tetrahydrate | -36.23 | 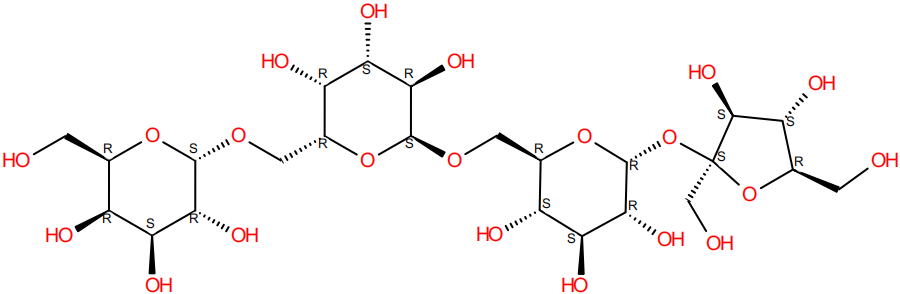 |
| Isomaltose | -36.12 | 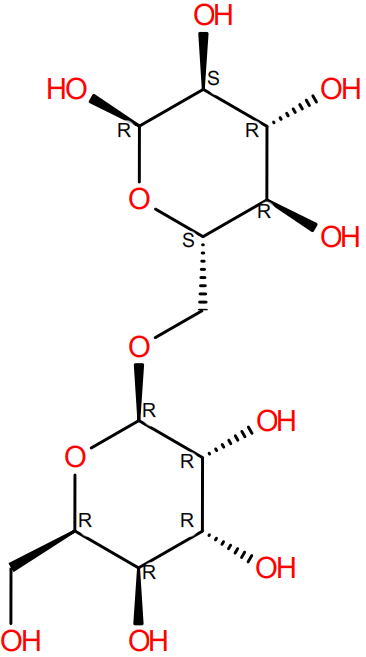 |
| 9-Oxononanoic Acid | -35.96 | 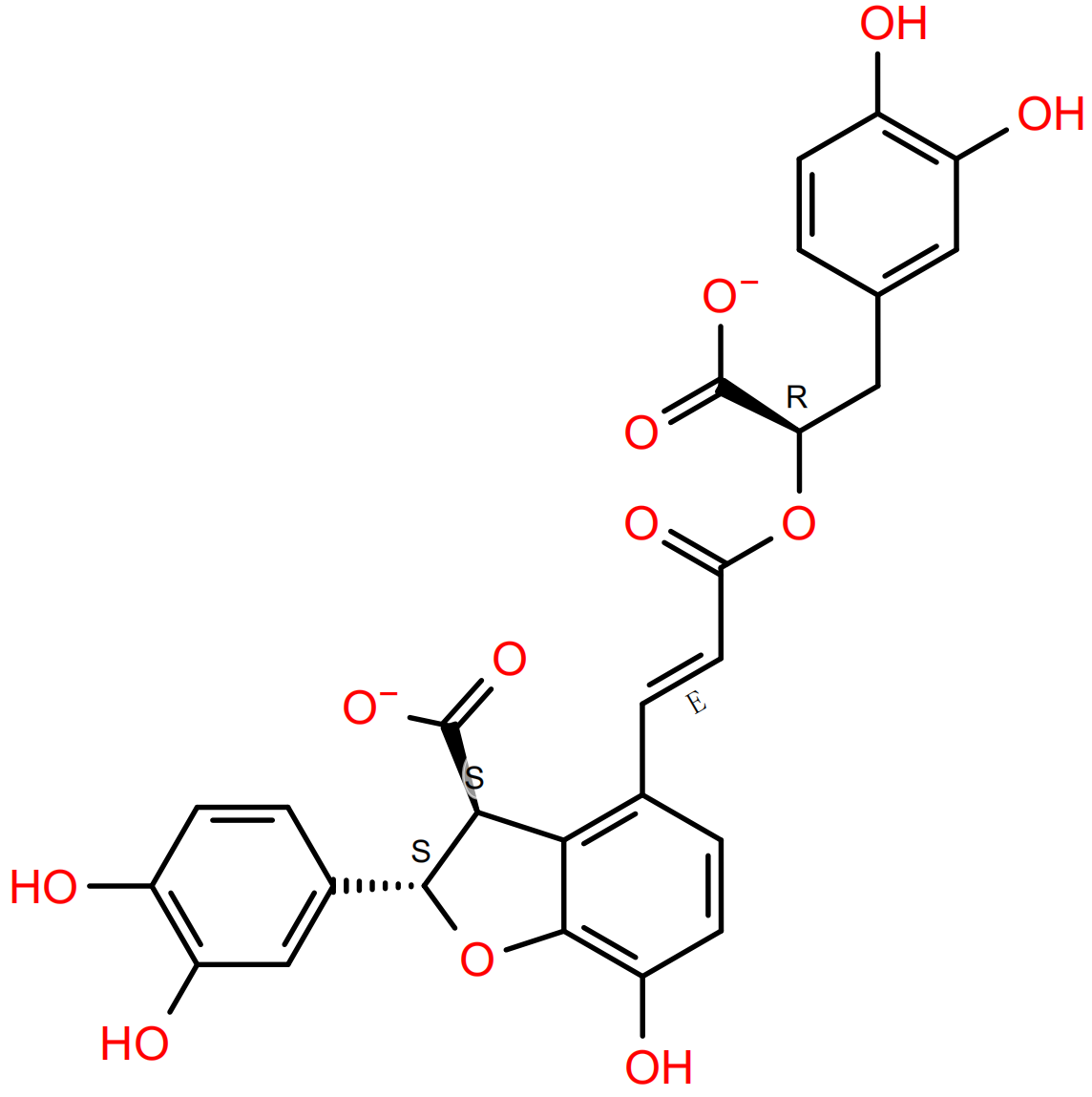 |
| β-Gentiobiose | -34.91 | 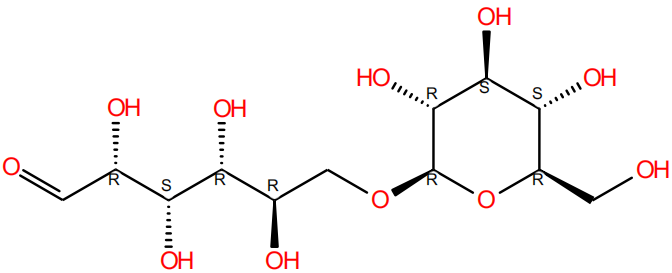 |
| Plantainoside D | -33.94 | 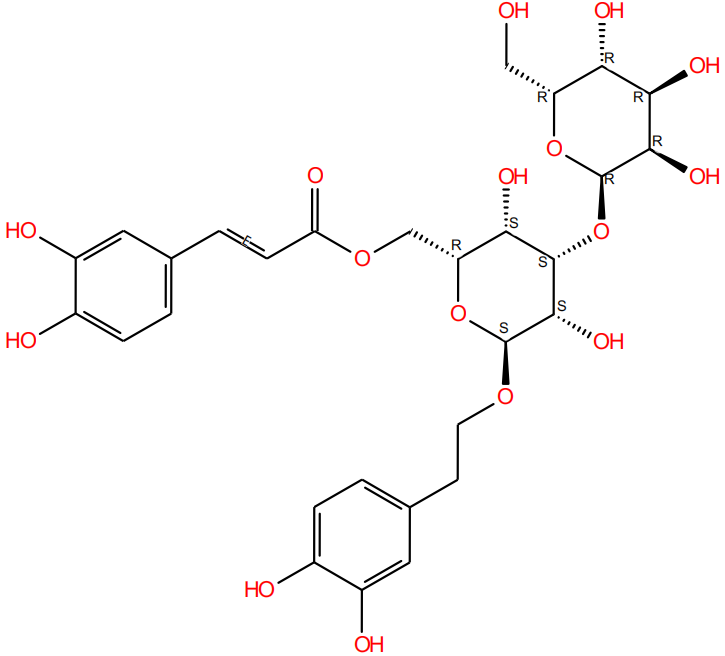 |
| Salvianolic acid B | -32.89 | 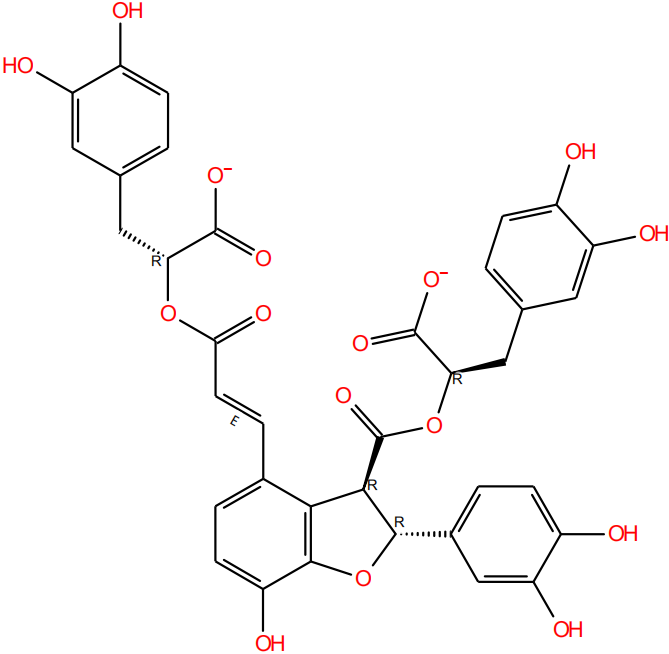 |
| Maltopentaose | -32.4 | 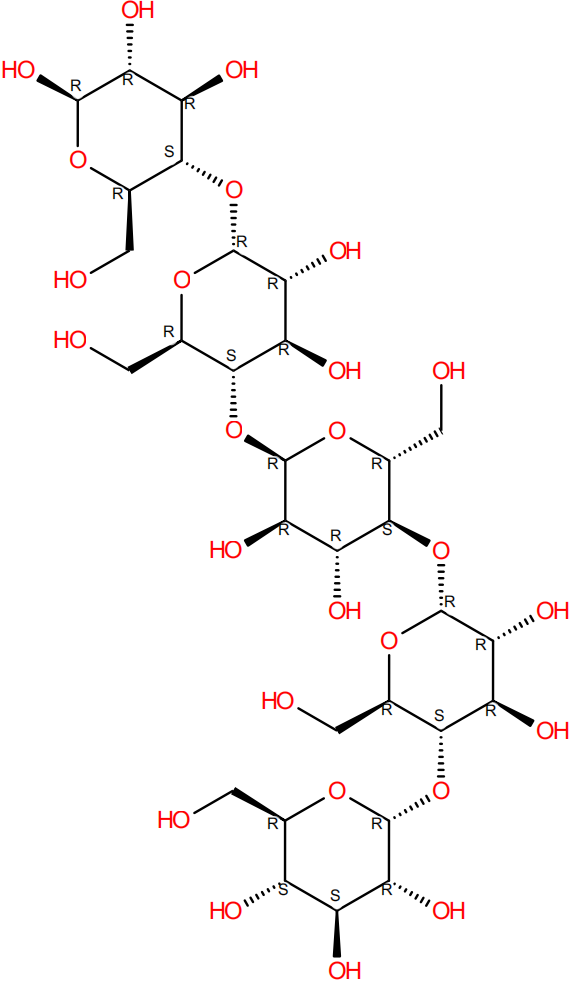 |
| Troxerutin | -31.61 | 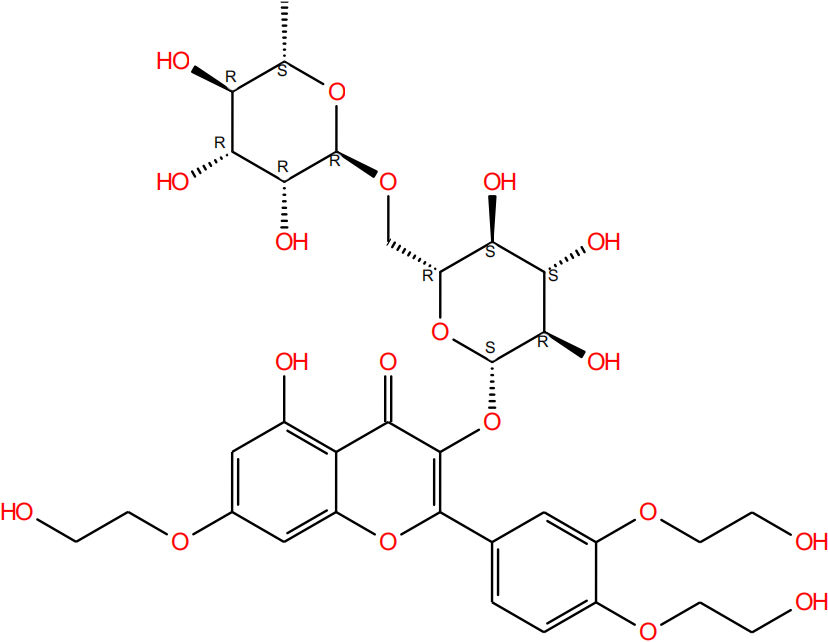 |
| Maltotetraose | -31.46 | 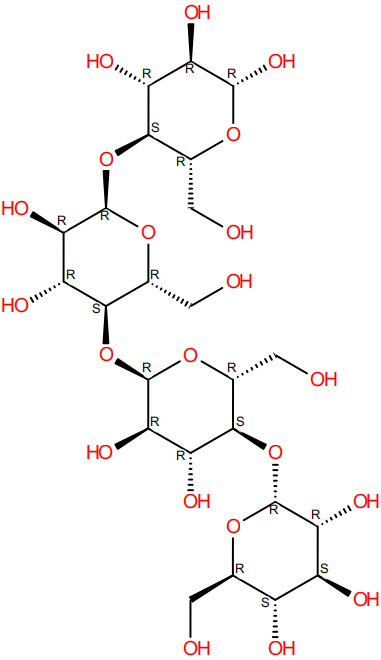 |
| Lactitol | -31.27 | 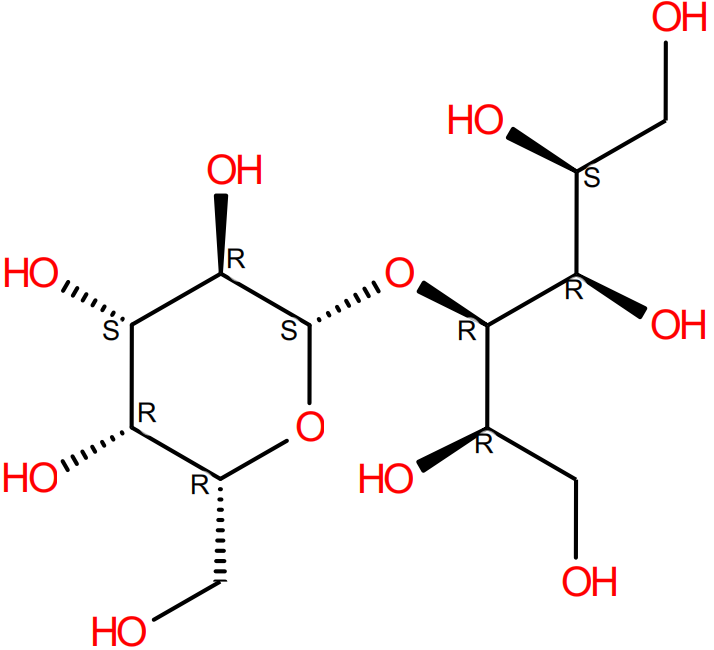 |

**Table S3.** Cox proportional hazards analysis of the effect of different doses of MLB on the survival rate of mice.

| **Comparison** | **HR (95% CI)** | ***P* value** |
| --- | --- | --- |
| Sham vs CLP | 138.577 (2.543-7881.088) | 0.016 |
| CLP vs CLP+MLB-L | 0.715 (0.363-1.407) | 0.331 |
| CLP vs CLP+MLB-H | 0.573 (0.385-0.855) | 0.006 |

**Table S4.** Statistical data of effect sizes with 95% CIs for barrier endpoints in Figure S3.

| **Comparison** | **Mean diff.^1^**  **(95% CI)** | **Cohen's *d***  **(95% CI)** | ***P* value** |
| --- | --- | --- | --- |
| **EB concentration (μg/g)** |  |  |  |
| Sham vs CLP | 4.419  (3.014 to 5.824) | 5.375  (2.945 to 7.805) | <0.0001 |
| CLP vs CLP+MLB-L | -1.398  (-2.803 to 0.007) | -1.344  (-2.597 to -0.091) | 0.0515 |
| CLP vs CLP+MLB-H | -3.451  (-4.857 to -2.046) | -3.854  (-5.766 to -1.941) | <0.0001 |
| CLP+MLB-L vs CLP+MLB-H | -2.054  (-3.459 to -0.649) | -2.688  (-4.249 to -1.127) | 0.0029 |
| **WD ratio** |  |  |  |
| Sham vs CLP | 5.515  (3.934 to 7.095) | 5.497  (3.024 to 7.971) | <0.0001 |
| CLP vs CLP+MLB-L | -1.486  (-3.067 to 0.094) | -1.376  (-2.635 to -0.118) | 0.07 |
| CLP vs CLP+MLB-H | -3.126  （-4.707 to -1.546） | -3.17  (-4.869 to -1.47) | 0.0001 |
| CLP+MLB-L vs CLP+MLB-H | -1.64  (-3.221 to -0.06) | -2.139  (-3.558 to -0.72) | 0.0402 |

¹ Effect size was calculated as the mean of the latter group minus the mean of the former group.

**Table S5.** Statistical data of effect sizes with 95% CIs for barrier endpoints in Figure 4.

| **Comparison** | **Mean diff.^1^**  **(95% CI)** | **Cohen's *d***  **(95% CI)** | ***P* value** |
| --- | --- | --- | --- |
| **EB concentration (μg/g)** |  |  |  |
| *Gpx4^f/f^*+CLP vs *Gpx4^f/f^Cdh5^Cre^*+CLP | 1.706  (0.459 to 2.953) | 2.679  （0.971 to 4.386） | 0.0061 |
| *Gpx4^f/f^*+CLP vs *Gpx4^f/f^*+CLP+MLB | -1.324  (-2.571 to -0.077) | -2.607  (-4.293 to -0.921) | 0.0356 |
| *Gpx4^f/f^Cdh5^Cre^*+CLP vs *Gpx4^f/f^Cdh5^Cre^*+CLP+MLB | -1.722  (-2.969 to -0.475) | -2.430  (-4.064 to -0.795) | 0.0057 |
| *Gpx4^f/f^*+CLP+MLB vs *Gpx4^f/f^Cdh5^Cre^*+CLP+MLB | 1.308  (0.061 to 2.555) | 2.197  (0.627 to 3.766) | 0.0382 |
| **WD ratio** |  |  |  |
| *Gpx4^f/f^*+CLP vs *Gpx4^f/f^Cdh5^Cre^*+CLP | 2.350  (0.8895 to 3.811) | 2.938  (1.150 to 4.725) | 0.0015 |
| *Gpx4^f/f^*+CLP vs *Gpx4^f/f^*+CLP+MLB | -1.816  (-3.277 to -0.356) | -2.475  (-4.122 to -0.828) | 0.0126 |
| *Gpx4^f/f^Cdh5^Cre^*+CLP vs *Gpx4^f/f^Cdh5^Cre^*+CLP+MLB | -1.430  (-2.891 to -0.031) | -2.014  (-3.536 to -0.492) | 0.056 |
| *Gpx4^f/f^*+CLP+MLB vs *Gpx4^f/f^Cdh5^Cre^*+CLP+MLB | 2.736  (1.275 to 4.197) | 4.313  (2.053 to 6.574) | 0.0003 |

¹ Effect size was calculated as the mean of the latter group minus the mean of the former group.

**Table S6.** Statistical data of effect sizes with 95% CIs for barrier endpoints in Figure S4.

| **Comparison** | **Mean diff.^1^**  **(95% CI)** | **Cohen's d**  **(95% CI)** | ***P* value** |
| --- | --- | --- | --- |
| **EB concentration (μg/g)** |  |  |  |
| *Gpx4^f/f^*+LPS vs *Gpx4^f/f^Cdh5^Cre^*+LPS | 3.582  (2.306 to 4.858) | 6.525  (3.408 to 9.642) | <0.0001 |
| *Gpx4^f/f^*+LPS vs *Gpx4^f/f^*+LPS+MLB | -1.392  (-2.668 to -0.116) | -2.992  (-4.796 to -1.187) | 0.0302 |
| *Gpx4^f/f^Cdh5^Cre^*+LPS vs *Gpx4^f/f^Cdh5^Cre^*+LPS+MLB | -1.124  (-2.400 to 0.152) | -1.477  (-2.875 to -0.078) | 0.0946 |
| *Gpx4^f/f^*+LPS+MLB vs *Gpx4^f/f^Cdh5^Cre^*+LPS+MLB | 3.850  (2.574 to 5.126) | 5.474  (2.774 to 8.175) | <0.0001 |
| **WD ratio** |  |  |  |
| *Gpx4^f/f^*+LPS vs *Gpx4^f/f^Cdh5^Cre^*+LPS | 2.680  (1.569 to 3.79) | 5.652  (2.882 to 8.423) | <0.0001 |
| *Gpx4^f/f^*+LPS vs *Gpx4^f/f^*+LPS+MLB | -2.050  (-3.161 to -0.939) | -5.224  (-7.827 to -2.620) | 0.0004 |
| *Gpx4^f/f^Cdh5^Cre^*+LPS vs *Gpx4^f/f^Cdh5^Cre^*+LPS+MLB | -0.550  (-1.661 to 0.561) | -0.820  (-2.111 to 0.470) | 0.508 |
| *Gpx4^f/f^*+LPS+MLB vs *Gpx4^f/f^Cdh5^Cre^*+LPS+MLB | 4.180  (3.069 to 5.291) | 6.792  (3.567 to 10.016) | <0.0001 |

¹ Effect size was calculated as the mean of the latter group minus the mean of the former group.

**Table S7.** Statistical data of effect sizes with 95% CIs for EB concentration in Figure 7.

| **Comparison** | **Mean diff.^1^**  **(95% CI)** | **Cohen's *d***  **(95% CI)** | ***P* value** |
| --- | --- | --- | --- |
| GPX4-WT vs GPX4-WT+CLP | 2.358  (1.496 to 3.22) | 6.215  (3.222 to 9.207) | <0.0001 |
| GPX4-WT+CLP vs GPX4-WT+CLP+MLB | -1.641  (-2.502 to -0.779) | -4.23  (-6.46 to -2) | 0.0003 |
| GPX4-WT+CLP+MLB vs GPX4-G79S+CLP+MLB | 1.664  (0.802 to 2.525) | 3.558  (1.566 to 5.55) | 0.0002 |

¹ Effect size was calculated as the mean of the latter group minus the mean of the former group.

**Table S8.** Cox regression analysis of the effects of MLB, MitoQ alone and in combination on the survival rate of CLP mice.

| **Comparison** | **HR (95% CI)** | ***P* value** |
| --- | --- | --- |
| Sham vs CLP | 138.577 (2.543-7881.088) | 0.016 |
| CLP vs CLP+MLB | 0.573 (0.385-0.855) | 0.006 |
| CLP vs CLP+MitoQ | 0.685 (0.473-0.992) | 0.045 |
| CLP+MLB vs CLP+MitoQ | 1.154 (0.519-2.567) | 0.726 |
| CLP+MLB vs CLP+MLB +MitoQ | 0.557 (0.312-0.997) | 0.049 |
| CLP+ MitoQ vs CLP+MLB +MitoQ | 0.229 (0.073-0.714) | 0.011 |

**Table S9.** Statistical data of effect sizes with 95% CIs for barrier endpoints in Figure 8.

| **Comparison** | **Mean diff.^1^**  **(95% CI)** | **Cohen's *d***  **(95% CI)** | ***P* value** |
| --- | --- | --- | --- |
| **EB concentration (μg/g)** |  |  |  |
| Sham vs CLP | 4.194  (2.952 to 5.436) | 7.124  (3.765 to 10.483) | <0.0001 |
| CLP vs CLP+MLB | -1.404  (-2.646 to -0.162) | -2.06  (-3.594 to -0.527) | 0.0221 |
| CLP vs CLP+MitoQ | -1.538  (-2.78 to -0.296) | -2.004  (-3.523 to -0.485) | 0.0109 |
| CLP vs CLP+MLB+MitoQ | -2.924  (-4.166 to -1.682) | -4.219  (-6.446 to -1.993) | <0.0001 |
| CLP+MLB vs CLP+MLB+MitoQ | -1.52  (-2.762 to -0.278) | -2.949  (-4.739 to -1.158) | 0.012 |
| CLP+MitoQ vs CLP+MLB+MitoQ | -1.386  (-2.628 to -0.144) | -2.218  (-3.793 to -0.643) | 0.0242 |
| **WD ratio** |  |  |  |
| Sham vs CLP | 4.976  (3.621 to 6.331) | 8.097  (4.338 to 11.856) | <0.0001 |
| CLP vs CLP+MLB | -1.486  (-2.841 to -0.131) | -2.689  (-4.4 to -0.979) | 0.0273 |
| CLP vs CLP+MitoQ | -1.53  (-2.885 to -0.175) | -2.254  (-3.839 to -0.669) | 0.0222 |
| CLP vs CLP+MLB+MitoQ | -3.296  (-4.651 to -1.941) | -4.921  (-7.408 to -2.433) | <0.0001 |
| CLP+MLB vs CLP+MLB+MitoQ | -1.81  (-3.165 to -0.455) | -2.953  (-4.745 to -1.161) | 0.0057 |
| CLP+MitoQ vs CLP+MLB+MitoQ | -1.766  (-3.121 to -0.411) | -2.423  (-4.055 to -0.791) | 0.007 |

¹ Effect size was calculated as the mean of the latter group minus the mean of the former group.

**Table S10.** Statistical data of effect sizes with 95% CIs for EB concentration in Figure 10.

| **Comparison** | **Mean diff.^1^**  **(95% CI)** | **Cohen's *d***  **(95% CI)** | ***P* value** |
| --- | --- | --- | --- |
| CLP+MLB vs CLP+MLB@ADSC-Evs | -2.154  (-2.773 to -1.534) | -7.526  (-11.05 to -4.003) | <0.0001 |
| CLP+MLB vs CLP+MLB@PBP-ADSC-Evs | -2.99  (-3.609 to -2.371) | -13.332  (-19.305 to -7.359) | <0.0001 |
| CLP+MLB@ADSC-Evs vs CLP+MLB@PBP-ADSC-Evs | -0.836  (-1.455 to -0.217) | -4.28  (-6.529 to -2.032) | 0.0068 |

¹ Effect size was calculated as the mean of the latter group minus the mean of the former group.

**Table S11.** Primer pairs for Quantitative real-time PCR.

| Gene | Primer Sequence (5’-3’) |
| --- | --- |
| Human *GPX4-F* | GAGGCAAGACCGAAGTAAACTAC |
| Human *GPX4-R* | CCGAACTGGTTACACGGGAA |
| Human *FUNDC1-F* | CCTCCCCAAGACTATGAAAGTGA |
| Human *FUNDC1-R* | AAACACTCGATTCCACCACTG |
| Human *GAPDH-F* | GTGCCGCCTGGAGAAAC |
| Human *GAPDH-R* | AAGGTGGAAGAGTGGGAGT |
