# Supplementary material for: Targeting the GPX4–FUNDC1 Interaction with Magnesium Lithospermate B Attenuates Sepsis‐Associated Lung Injury
Source: Adv Sci (Weinh). 2026 Jan 30;13(20):e16488. doi: 10.1002/advs.202516488 (PMC13067869; doi:10.1002/advs.202516488)

Original Images of Western Blots

These are WB band images from this article. We have included the uncropped full membranes.

The red box highlights our target band.

Figure 2

M

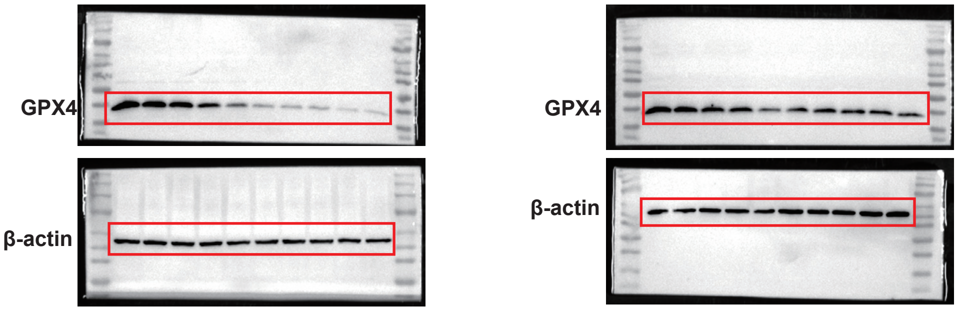

N

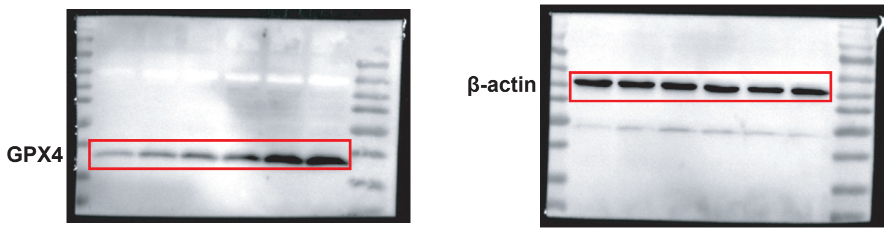

Figure 3

E

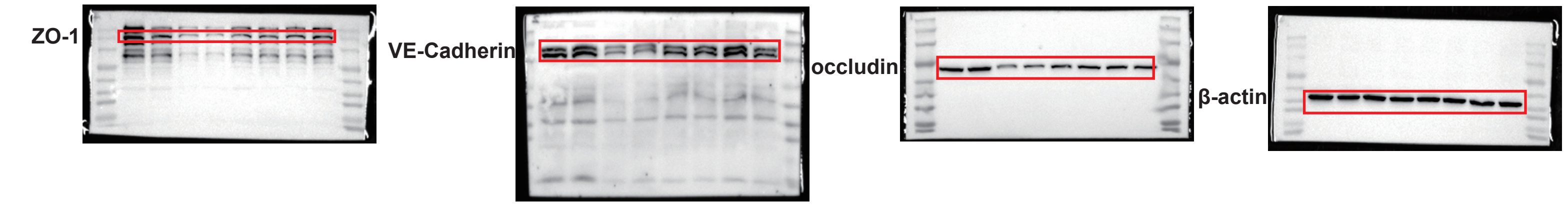

H

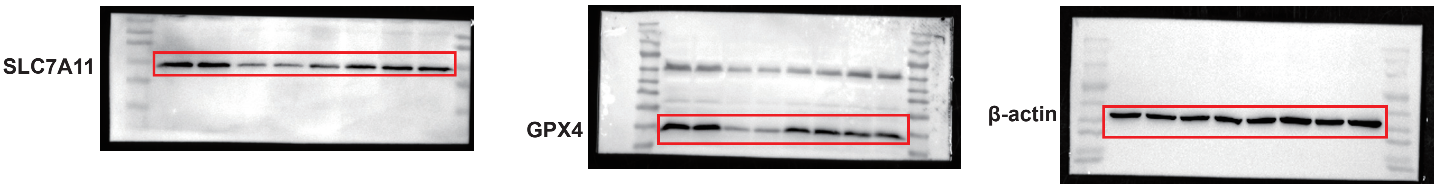

Figure 4

E

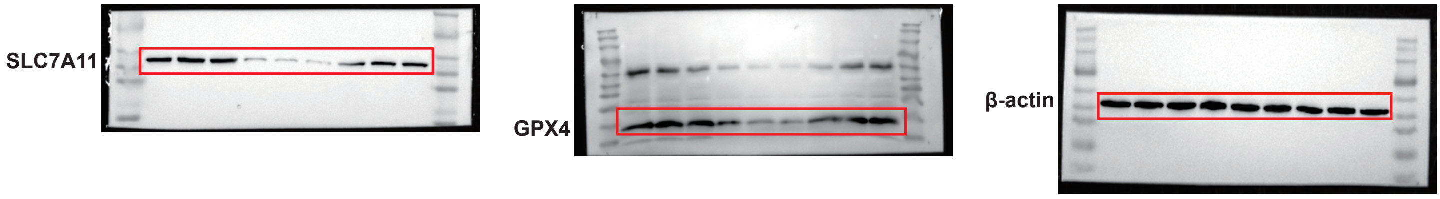

Figure 5

A

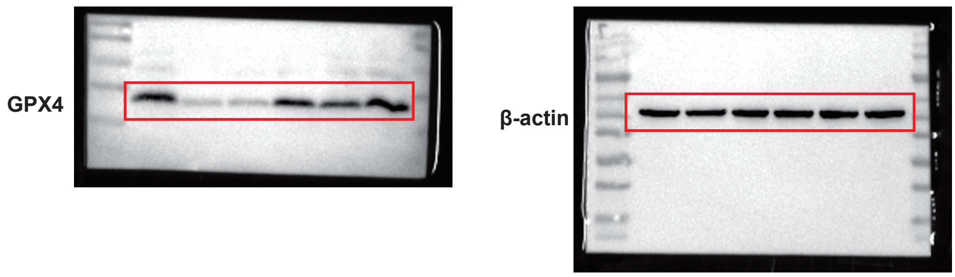

B

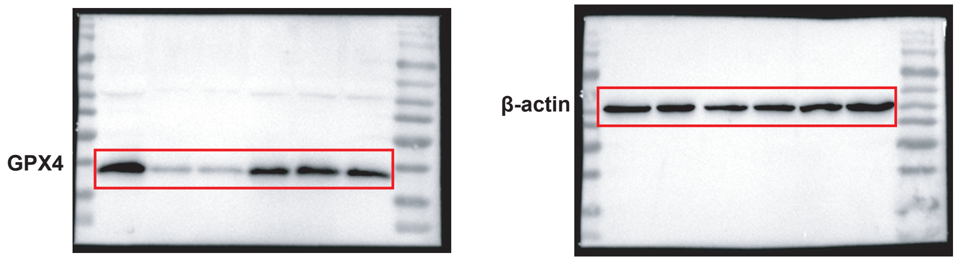

H

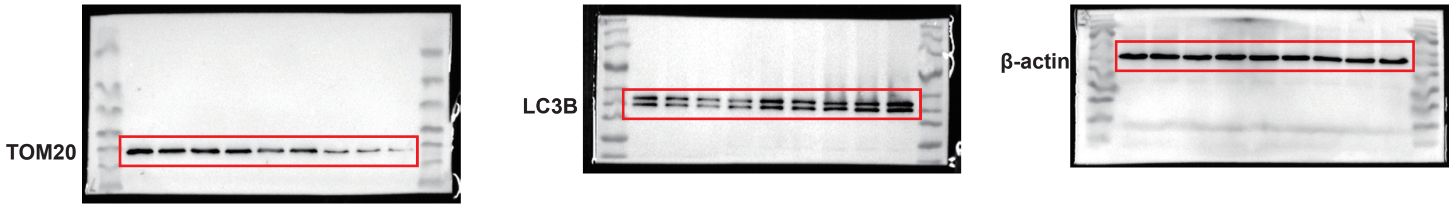

Figure 6

D

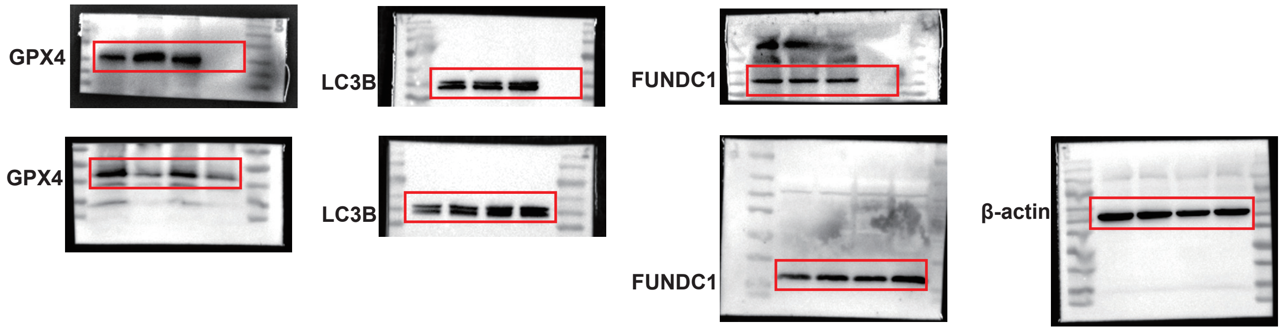

Figure 7

A

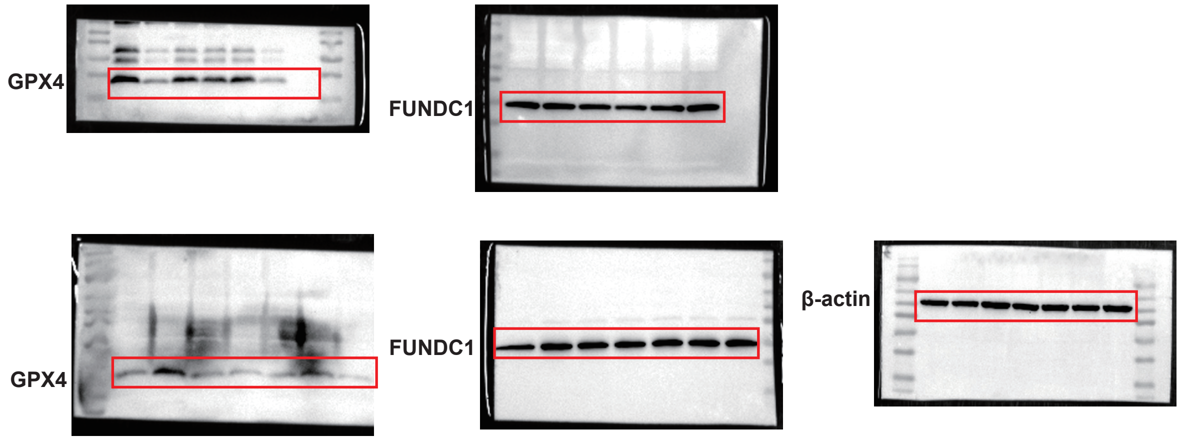

F

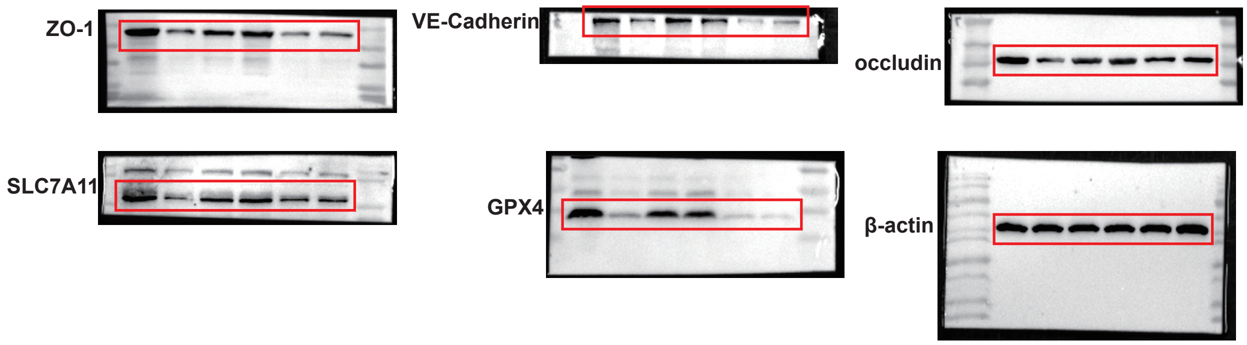

J

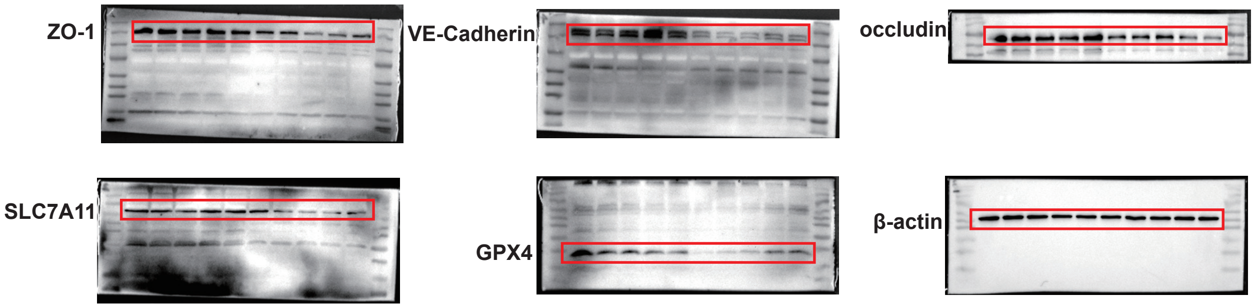

Figure 9

D

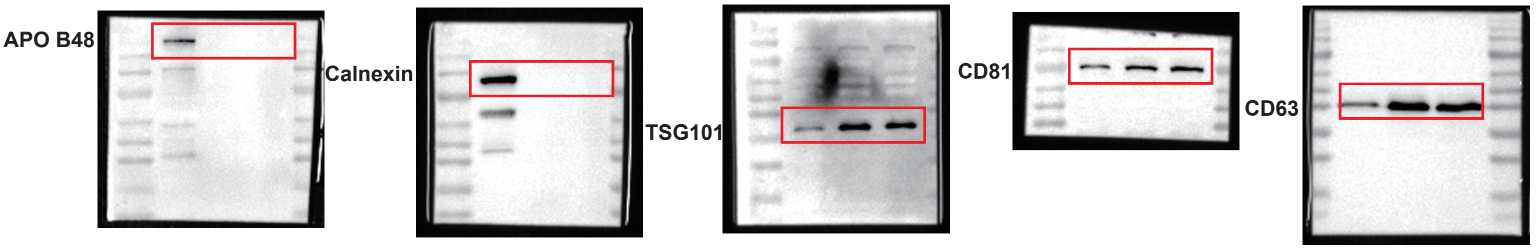

Figure S3

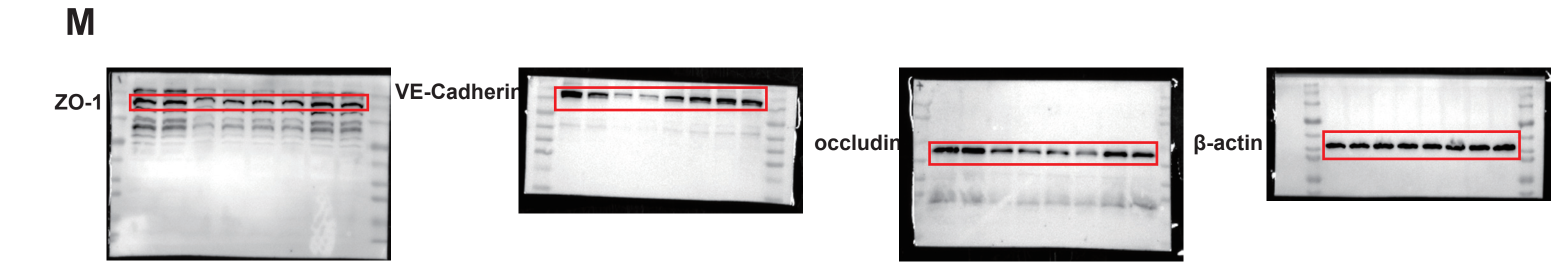

Figure S4

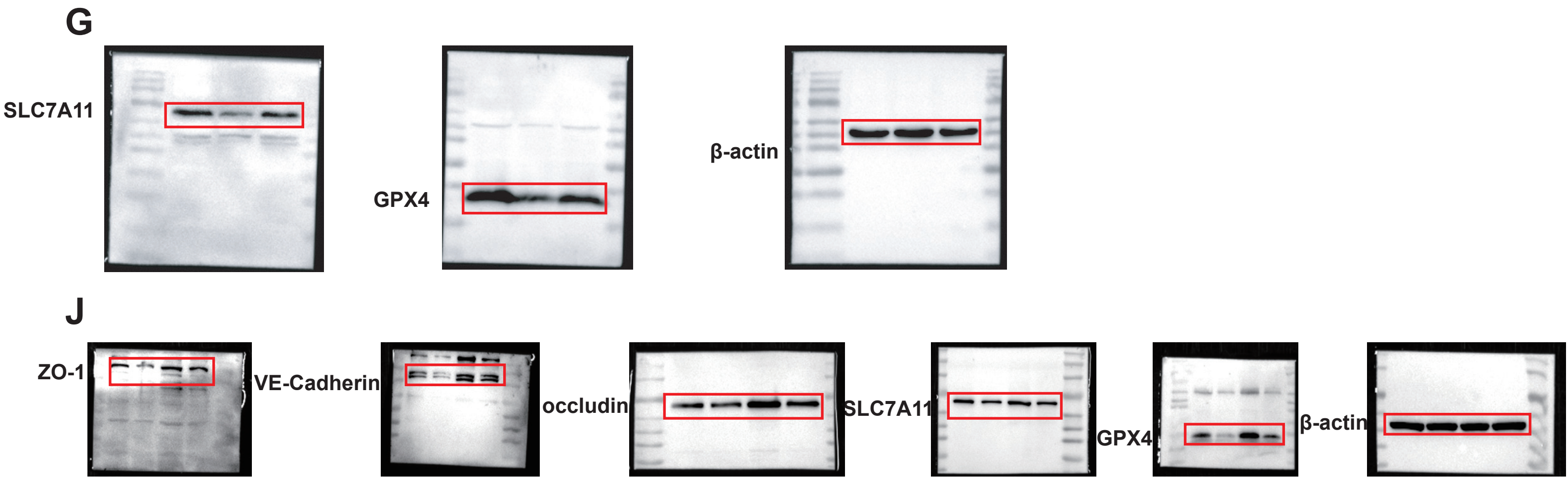

Figure S5

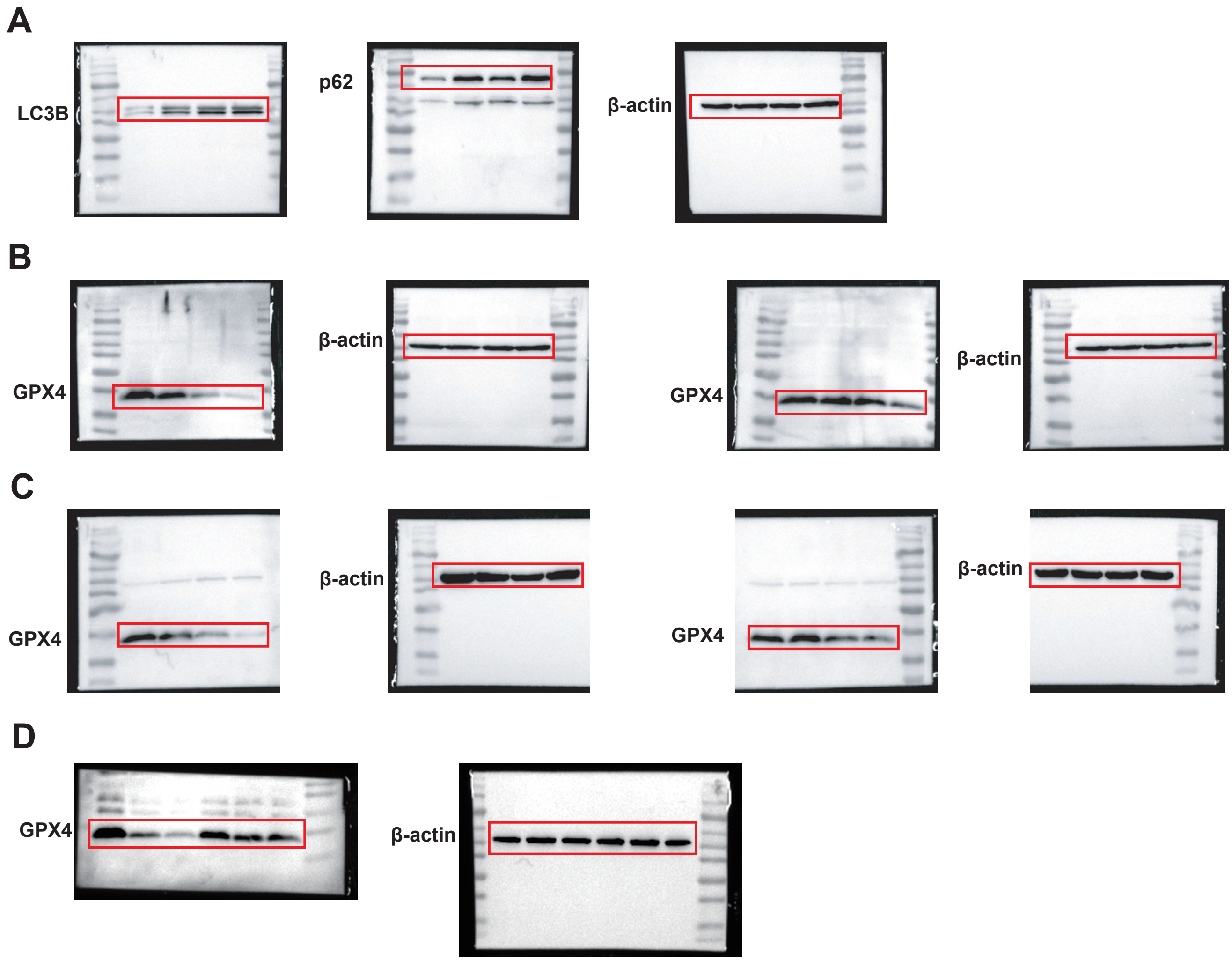

Figure S5

E

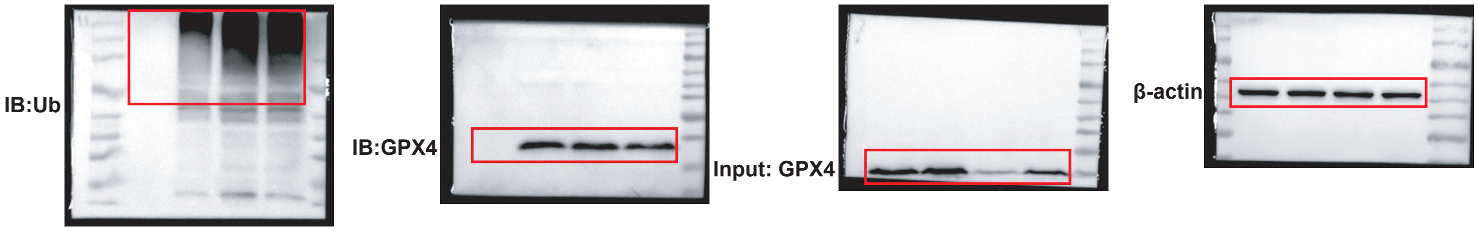

G

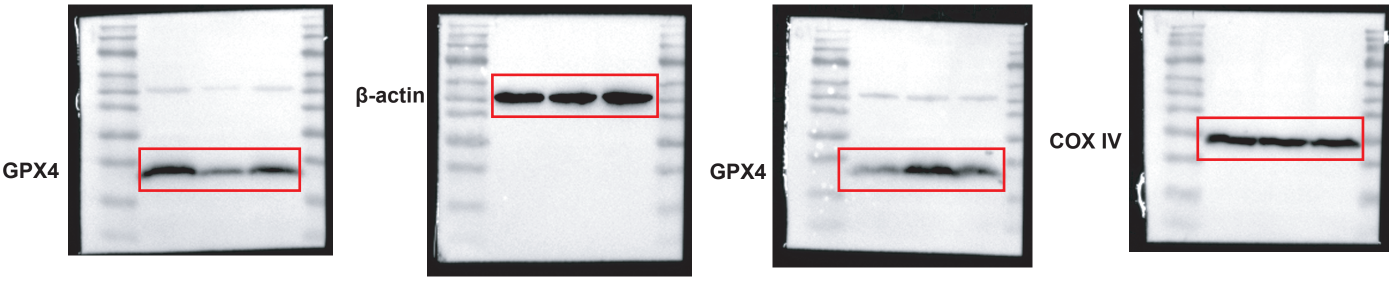

Figure S7

B

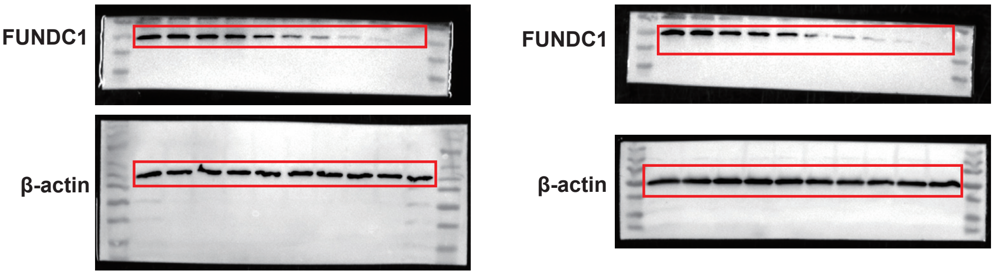

Figure S8

A

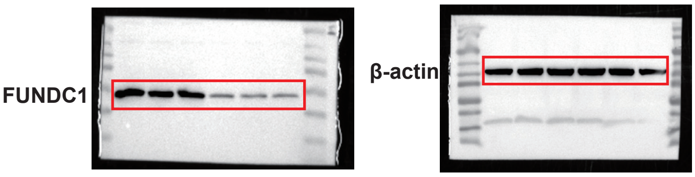

C

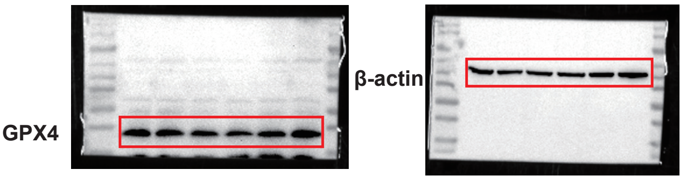

D

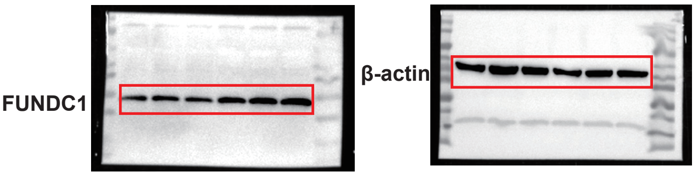

F

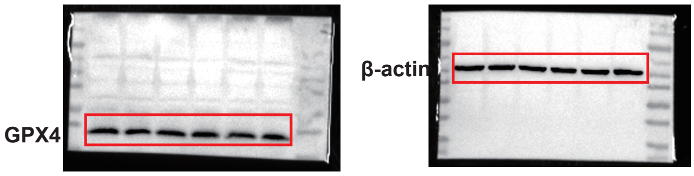

Figure S8

G

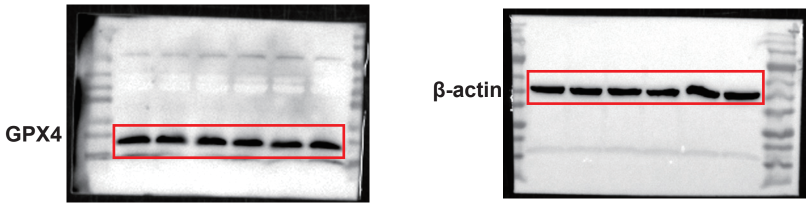

H

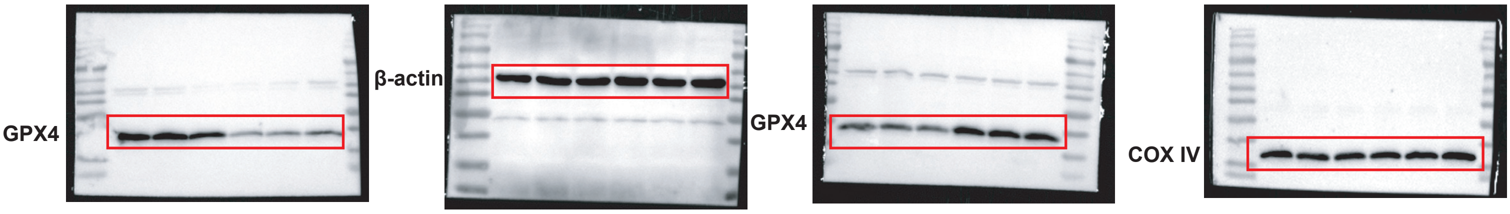

I

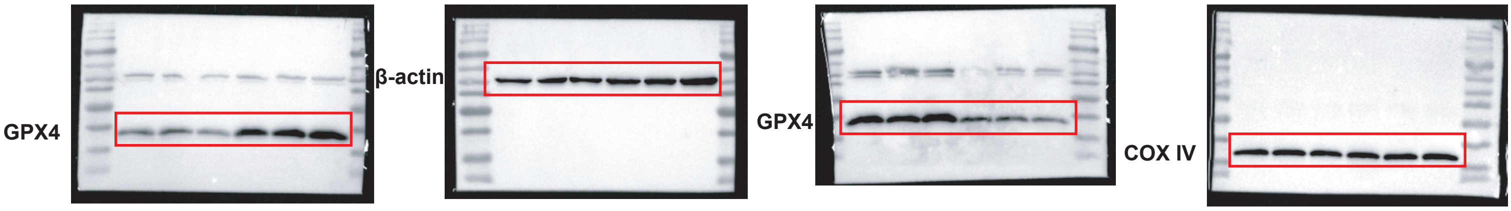

Figure S9

B

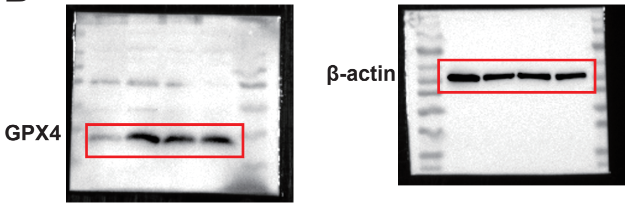

D

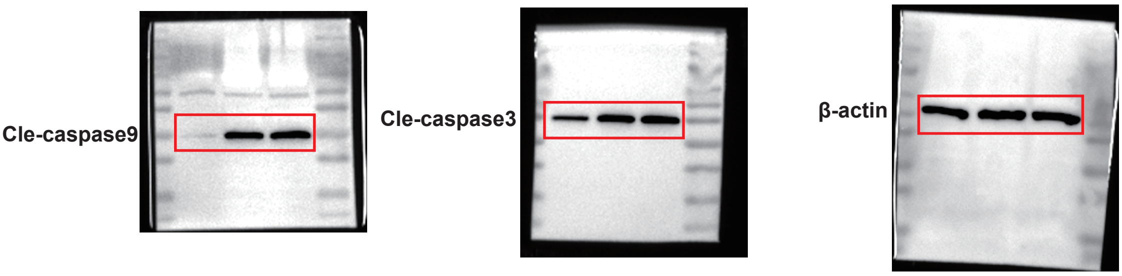

G

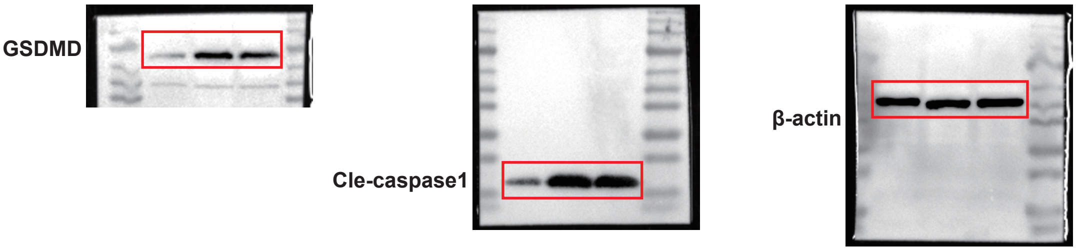

Figure S10

A

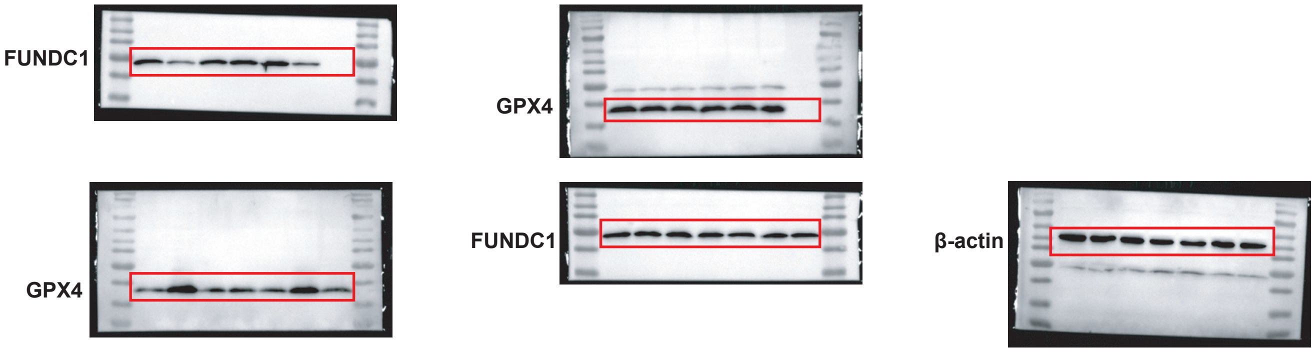

C

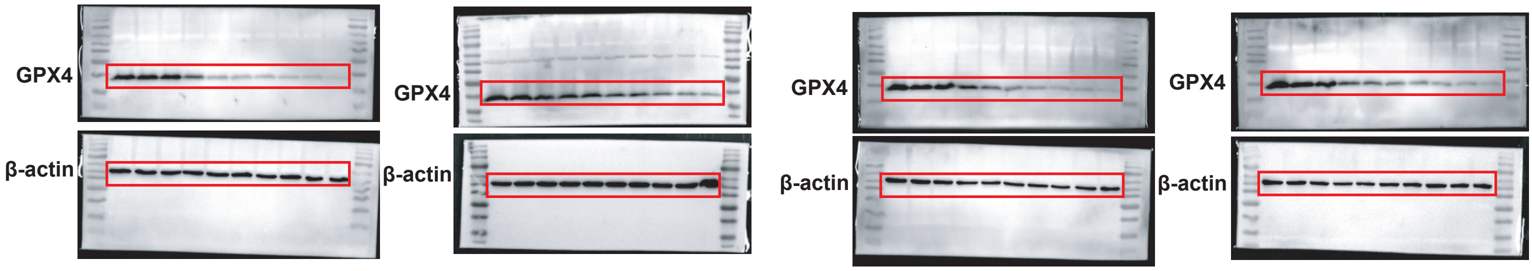

Supplement: Supplementary file 4 — Supporting File 4: advs74152‐sup‐0004‐Data.zip. [file ADVS-13-e16488-s003.zip › advs74152-sup-0004-Data/Original Images of Western Blots.pdf]
